# Evolution, biomechanics, and neurobiology converge to explain selective finger motor control

**DOI:** 10.1152/physrev.00030.2023

**Published:** 2024-02-22

**Authors:** Jing Xu, Firas Mawase, Marc H. Schieber

**Affiliations:** ^1^Department of Kinesiology, University of Georgia, Athens, Georgia, United States; ^2^Department of Biomedical Engineering, Israel Institute of Technology, Haifa, Israel; ^3^Departments of Neurology and Neuroscience, University of Rochester, Rochester, New York, United States

**Keywords:** dexterity, enslaving, hand function, individuation, nervous system

## Abstract

Humans use their fingers to perform a variety of tasks, from simple grasping to manipulating objects, to typing and playing musical instruments, a variety wider than any other species. The more sophisticated the task, the more it involves individuated finger movements, those in which one or more selected fingers perform an intended action while the motion of other digits is constrained. Here we review the neurobiology of such individuated finger movements. We consider their evolutionary origins, the extent to which finger movements are in fact individuated, and the evolved features of neuromuscular control that both enable and limit individuation. We go on to discuss other features of motor control that combine with individuation to create dexterity, the impairment of individuation by disease, and the broad extent of capabilities that individuation confers on humans. We comment on the challenges facing the development of a truly dexterous bionic hand. We conclude by identifying topics for future investigation that will advance our understanding of how neural networks interact across multiple regions of the central nervous system to create individuated movements for the skills humans use to express their cognitive activity.

## Graphical abstract

**Figure:**
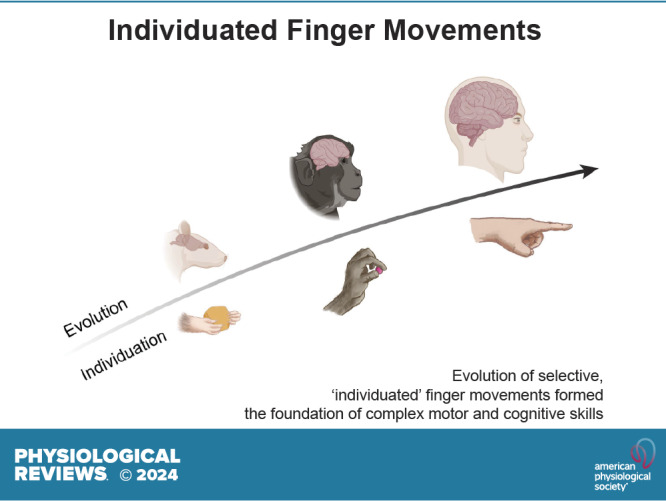

CLINICAL HIGHLIGHTS

The ability to move the fingers relatively selectively has increased along the evolutionary scale. Yet even in humans, whenever one digit moves, other digits move as well. Movement of unintended digits results in part from the biomechanics of the hand and its muscles and in part from the neural systems that control the fingers. These neural systems each include many single neurons with outputs that diverge to synapse in the spinal motoneuron pools of multiple muscles. Because these factors cause motion in unintended digits, the contraction of agonists to move any given digit is accompanied by the contraction of additional muscles to stabilize other digits and the wrist.

The primary motor cortex (M1) dominates control of voluntary movement in humans, acting in concert with other descending systems to sculpt the coordinated action of agonist, antagonist, and stabilizing muscles. During any finger movement, neural activity is distributed over a wide M1 territory that overlaps extensively with the territory active during the movement of other fingers. Consequently, cortical lesions never impair the function of only one digit. Lesions of M1 or the corticospinal tract impair relatively selective, or “individuated,” extension finger movements more than flexions. Separate mechanisms may underly recovery of strength versus individuation.

## 1. INTRODUCTION

When you type at a keyboard, you may think you are moving your fingers independently of one another. Yet, careful observers have known for some time that “…the simple movement of alternate flexion and extension of a digit is always accompanied by a similar movement of lesser range by its fellows. In short, a discrete movement of a single digit is a feat none of us is capable of” (1). Different authors have referred to “relatively independent” or “fractionated” finger movements (2), capturing the notion that although movements of one finger can be distinct from the others, no single finger is completely independent of the others.

To understand why this is so, we review the evolution of the fingers and the biomechanical factors that still constrain finger independence in the human hand. The neural control of the hand and fingers, of course, has evolved in parallel with the musculoskeletal apparatus, and we review how the nervous system acts to control multiple fingers simultaneously, even when we intend to move only one finger. We use the term “individuated,” meaning distinguished from others of the same kind, singled out, formed into a distinct entity, to further capture how movements of selected fingers have evolved from grasping with the entire hand.

We go on to examine how individuation contributes to dexterity, how impaired individuation manifests clinically, and how individuation of finger movements, and of other body parts as well, forms the substrate for expression of human cognitive activity. We discuss challenges for the development of bionic hands. Finally, we consider questions open to further investigation, as well as future directions in which an increased understanding of individuated finger movements may be used to improve our lives.

## 2. EVOLUTION OF FINGER MOVEMENTS IN VERTEBRATES

Insight into the evolution of the human capability for individuation can be gained by examining differences in selected living vertebrate species. All the movements we make presumably have evolved from the primordial movements of the oldest vertebrates, fish, and the fingers of our hands have evolved from the bony rays that supported their pectoral fins. The swimming movements of fish are controlled by oscillating central pattern generators (CPGs) in each segment of the spinal cord that alternately contract and relax that segment’s myotome on the right and left sides of the body (3) (see glossary for all definitions). For swimming, these contraction/relaxation oscillations in sequential segments are coordinated from head to tail to produce a traveling wave of body motion. As the pectoral fin and its supporting structures evolved into the forelimb, specialized CPGs that generated the oscillations of the pectoral fin evolved as well. CPG oscillations in the cervical and lumbar segments of the spinal cord initially evolved to drive the rhythmic extension/flexion phases of limb movements used for locomotion in reptiles such as turtles (4). Further evolution provided movements that are not necessarily oscillatory, the forelimb reach/return movements seen in rodents and carnivores and eventually the open/grasp movements of the primate hand, all of which are under increasingly direct control from the brainstem and, in mammals, the cerebral cortex (5–8).

The manner in which the fin/forepaw/hand can be used evolved in parallel. Turtles, for example, have five digits at the distal end of their forelimb that they use in walking, swimming, and digging, but not for grasping and manipulating objects. In contrast, rats use their forepaws to grasp food and bring it to the mouth, adjusting all the digits to the object either concurrently or in rapid succession, and sometimes grasping a small item between two adjacent digits as all the digits flex (9–11). Likewise, cats can shape their paw to retrieve food items they might otherwise drop (5, 12).

Among nonhuman primates, aye-ayes (native to Madagascar) have a specialized long, thin middle finger, which they tap relatively selectively and then probe into small holes to detect and capture prey (13). New world marmosets, which lack opposable thumbs, and squirrel monkeys, which have pseudo-opposable thumbs, both use only power grasps, closing all five digits around objects (14–17). New world capuchin monkeys, which also have pseudo-opposable thumbs, not only use power grasps but also use precision grips between the thumb and other digits, most often the index finger (14). Old-world macaque monkeys use an even more dexterous precision pinch, opposing the tip of the thumb to the tip of the index finger while keeping the middle, ring, and little fingers out of the way, which enables them to grasp objects as small as a louse egg (18). The great apes (chimpanzees, orangutans, and gorillas) also use such a precision pinch for picking up small objects, as do humans (19). The kinematics of grasping in humans is similar to that in macaques, but the postures of the human hand are more object specific, and the human joint angles are less correlated with one another (20), i.e., more individuated.

Macaques use a wide variety of grasps to handle and manipulate objects of various shapes and sizes, some of which involve differential activity in the radial versus ulnar digits (19, 21). Macaques also have been observed to hold one item by flexing the ring and little fingers against the palm while manipulating another item with the thumb, index, and middle fingers (21). Furthermore, macaques, apes, and humans can transfer an item from the initial grasp made with the radial digits to storage with the ulnar digits, a within-hand manipulation (19). In picking up a series of coins, for example, you may initially grasp each coin with your thumb and index finger and then transfer it to be held against the palm with your middle, ring, and little fingers while you pick up the next coin with your thumb and index finger. We view precision pinch and within-hand manipulations as movements that evolution has individuated from whole hand power grasps, which involve parallel flexion of all five digits.

## 3. QUANTIFYING THE BEHAVIORAL CAPACITY FOR INDIVIDUATION OF EACH DIGIT

Individuated finger movements, the muscles producing them, and their neural control, all have been studied most extensively in macaque monkeys and in humans. The ability to individuate the movement of each digit has been quantified only in rhesus macaques and in humans.

### 3.1. Macaque Monkeys

After extensive training to flex or extend one digit (or the wrist) at a time, each of the two rhesus macaques was able to move the instructed digit more than, but not without, the motion of other digits (22). Thumb flexion and wrist flexion or extension were each accompanied by relatively little motion of other digits, but all other instructed flexion or extension movements involved simultaneous movement of noninstructed digits. An “individuation index” quantifying the degree to which noninstructed digits moved during the movement of the instructed digit showed that in general rhesus flexions were more individuated than extensions. Flexion of digits 1, 2, and 5 (the thumb, index, and little finger, respectively, with individuation indexes ranking in this order) tended to be more highly individuated than flexion of digits 3 and 4 (the middle and ring finger, respectively). Macaques thus can individuate finger movements to some extent but cannot move their fingers independently.

### 3.2. Human Individuation: Isotonic Movement

**FIGURE 1.**
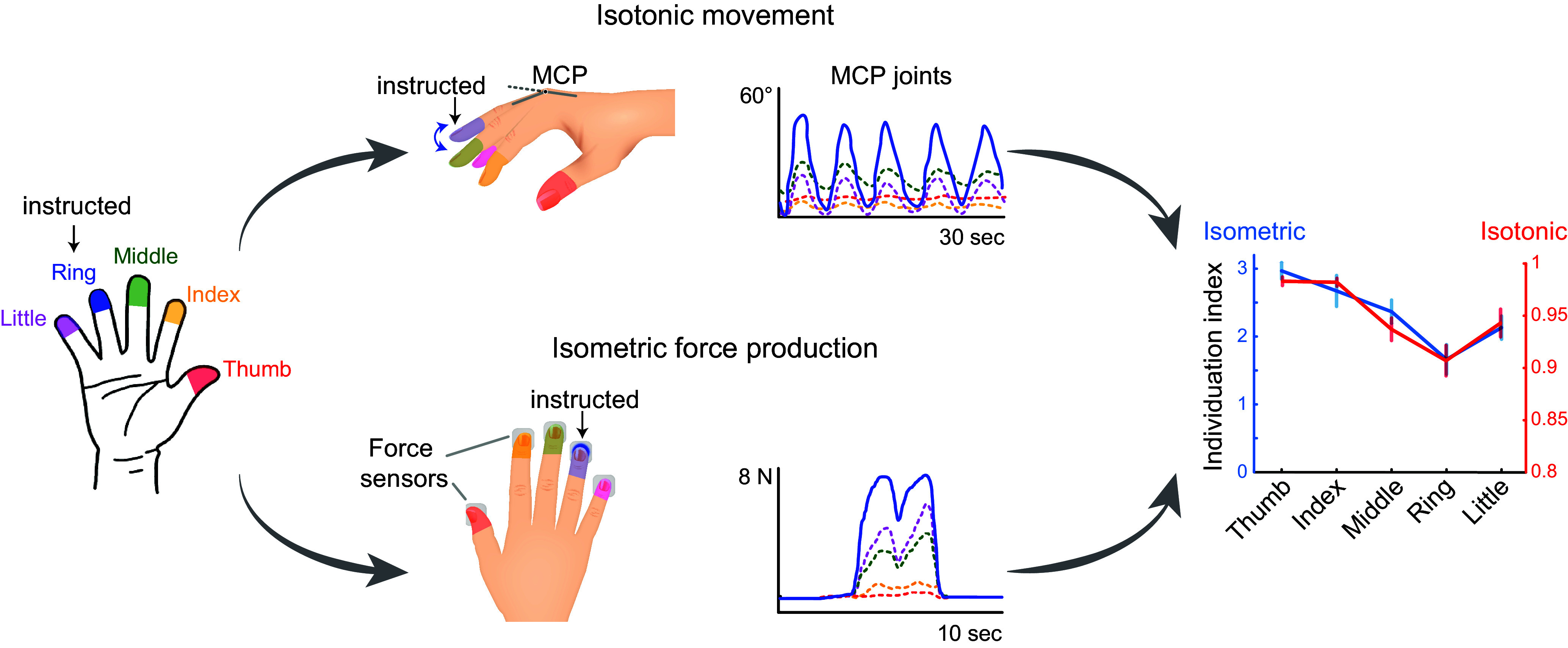
Quantifying individuation in the human hand. Examples are shown of isotonic movements (*top*) and isometric force production (*bottom*) by the human ring finger (blue), along with the simultaneous movement or force production in each of the other, noninstructed digits, color coded as shown by the hand diagram at the *far left*. The *far right* shows the individuation indexes derived when each digit is the instructed digit using isotonic data (red) or isometric data (blue). MCP, metacarpophalangeal. Isotonic data are reproduced from Ref. 23, with permission from *Journal of Neuroscience*; isometric data are from Ref. 24, with permission from *Cell Reports*.

Recordings of human finger kinematics during isotonic movements (joint rotation against little or no external load) likewise show that humans move other digits to some extent while intending to move only a single finger (FIGURE 1, *top*). When asked to make self-paced, oscillatory flexion/extension movements of a single digit, intended movement of the thumb was accompanied by little if any movement of the other digits, but such was not the case for the fingers (23, 25). Although human individuation indexes were higher than those of macaques, like macaques, human flexion/extension movements on average were most highly individuated for the thumb and least individuated for the ring finger. Interestingly, human flexion/extension individuation indexes are not particularly different for the right (dominant) versus left hand but are marginally higher in females than in males (26).

Flexion and extension movements of the fingers are powered largely by the extrinsic finger muscles that have bellies in the forearm and give off tendons to multiple digits: the flexor digitorum superficialis (FDS), flexor digitorum profundus (FDP), and extensor digitorum communis (EDC). Each of these multitendoned muscles might contribute to flexion/extension motion of unintended digits (see sect. 4.3). In contrast, abduction and adduction movements of the fingers are driven primarily by intrinsic hand muscles, the palmar and dorsal interossei, which have bellies in the palm. These muscles each act nominally on only a single digit. Nevertheless, when human subjects were asked to produce abduction/adduction movements of one digit, lesser movement occurred in other digits as well (27). Although human finger movements are more highly individuated than those of macaques, when instructed to move a given digit, either in flexion/extension or abduction/adduction, humans produce some lesser motion in other digits at the same time.

### 3.3. Human Finger Enslaving: Isometric Force Production

When fingers come into contact with an object, particularly a hard object like a golf ball, conditions change from isotonic to isometric (force production against an unyielding load with little or no joint rotation). Such a transition in mechanical constraints is accompanied by a change in the neural strategy for controlling the digits (28). Considering this change in neural control together with the potentially reduced effect of biomechanical coupling among the static digits, one might expect the isometric forces exerted by the different digits to be independent of one another. However, they are not (FIGURE 1, *bottom*).

When a human subject intends to exert force with one or more digits, the tendency of additional digits to unintentionally exert lesser force has been described most often as “enslaving,” i.e., force production by the master digit(s) enslaves unintended force production in other digits (29–31). Enslaving can be viewed as the inverse of individuation: a lower degree of enslaving corresponds to a higher degree of individuation. For example, the thumb and index finger are the most highly individuated and the least enslaving digits. In general, digits adjacent to the master digit produce more unintended force than nonadjacent digits (31, 32), and enslaving of noninstructed digits increases as the master digit produces more force (33). Furthermore, enslaving is higher during the production of extension forces than flexion forces (32, 34). After multiday training of single- and multifinger isometric extension or flexion force production, enslaving decreases (individuation increases), but generalization between flexion and extension is asymmetric: training on extension forces results in less enslaving when producing flexion forces but not vice versa (24).

As with isotonic individuated finger movements, one might think that enslaving results primarily from the action of the multitendoned extrinsic finger muscles. However, whether flexion forces are produced at the distal finger pads where the extrinsic muscles contribute the majority of the flexion forces, or at the proximal interphalangeal joints where the interossei produce most of the force, the patterns of enslaving are similar (31, 35, 36). Furthermore, enslaving also is evident when humans exert abduction or adduction finger forces produced by the intrinsic muscles of the hand that each act on only one digit (37). As with flexion/extension movements (38), abduction/adduction force production by the thumb or index finger shows less enslaving than abduction/adduction of the ring or little finger. Similar patterns of enslaving are found when fingertip forces are measured simultaneously in three Cartesian dimensions (39). These observations indicate that enslaving results largely from the central, neural control of the fingers.

## 4. FACTORS CONSTRAINING/PERMITTING INDIVIDUATION

Why do other digits move when we intend to move or exert force with only one? Four major factors contribute to the unintended motion of other digits. First, the digits are passively coupled by the soft tissues of the hand. Second, active contraction of the muscles that move one digit results in biomechanical interactions among the digits and wrist that produce unintended motion in other digits. Third, multitendoned muscles, their neuromuscular compartments (see BOX 1 and Refs. 40 and 41), and their motor units do not necessarily put tension solely on one finger tendon at a time. Fourth, many of the last-order central inputs to the spinal motoneuron pools innervating muscles that act on a given digit diverge to simultaneously facilitate activity in motoneuron pools acting on other digits. We review these factors, proceeding from peripheral to central. Based on these factors, we then present a theoretical speculation concerning the evolution of the neuromuscular apparatus for individuated finger movements.

### 4.1. Passive Coupling by Soft Tissues

Under isotonic conditions, where motion of the digits is relatively unimpeded by external loads, a major factor limiting digit independence is passive coupling between the digits produced by the soft tissues of the hand and its muscles. When one human digit at a time was strapped to a small paddle that held the digit’s interphalangeal joints fully extended and the metacarpophalangeal (MCP) joint then was rotated passively, almost as much movement occurred in other digits as when a similar MCP joint rotation was produced actively by the subject. Most of the motion of other digits thus was produced passively. Individuation indexes were only slightly lower in the active than in the passive condition (25). Similar results were obtained whether the MCP joint was rotated through almost its entire range of motion or only a small arc. Passive coupling between the digits under these conditions was a substantial factor constraining individuation of human finger movements.

**FIGURE 2.**
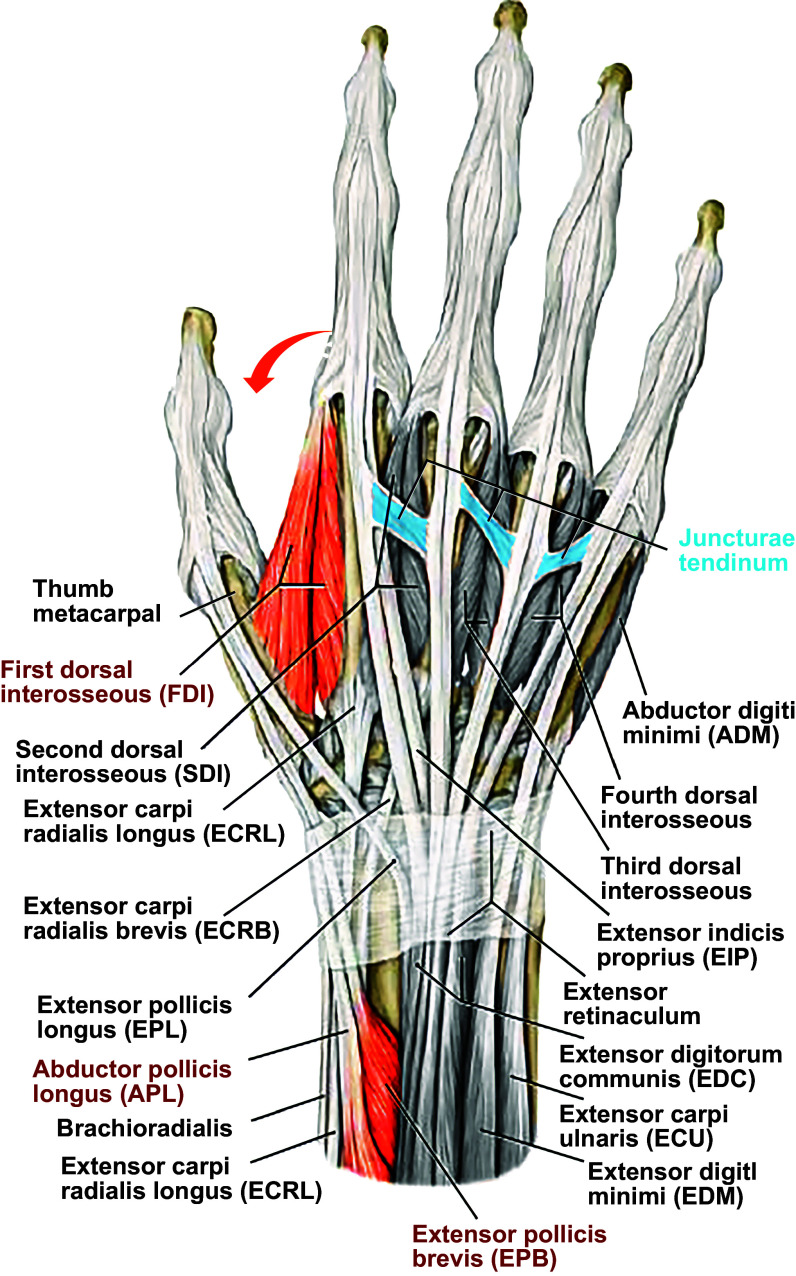
Dorsal view of the muscles and tendons of the human hand. Blue shading marks the juncturae tendinum that passively interconnect adjacent tendons of extensor digitorum communis (EDC) to the index, middle, ring, and little fingers. Red shading indicates that because the lateral head of first dorsal interosseous (FDI) originates from the thumb’s metacarpal bone, active contraction of FDI to abduct the index finger (red arrow) tends to adduct the thumb; abductor pollicis longus (APL) and abductor pollicis brevis (EPB) therefore contract along with FDI to minimize such unintended motion of the thumb. Image modified from Ref. 43, with permission from Thieme Medical Publishers, Inc.

Passive coupling is in itself multifactorial. The tissue of the web space couples adjacent digits to some degree, particularly if the adjacent digits are fully extended (42). Additional coupling results from interconnections between tendons to different fingers. Just proximal to the metacarpal heads, juncturae tendinum link adjacent tendons of EDC to the four fingers (FIGURE 2, blue), putting some degree of tension on adjacent tendons as the neuromuscular compartment of EDC serving one finger contracts or as an adjacent finger is flexed (42, 44). These interconnections tend to be progressively thicker between EDC tendons for the more ulnar digits (45). The tendons of the human FDP likewise are interconnected by tendinous slips in the carpal tunnel and by the bipennate origins of the lumbrical muscles from adjacent FDP tendons in the palm (46, 47). The extent to which such tendon interconnections from one finger exert passive forces on tendons to other fingers is complex and depends on such factors as the slack of the tendons, and the insertion angle and stiffness of the connections (48).

Insight into the origins of these intertendinous connections in humans can be gained by comparison with macaques. Whereas in humans the FDP tendons to the four fingers are distinct in the forearm, in rhesus macaques the FDP muscle mass inserts onto a single flat sheet of tendon that continues from the forearm through the carpal tunnel and into the palm (49, 50). Only after entering the palm does this tendon sheet give off separate tendons to each of the five digits. Because macaques have no separate muscle that flexes the tip of the thumb, flexing the tip of the macaque thumb inevitably involves flexion of the tip of the index and middle fingers, if not the ring and little fingers as well. In contrast, the human flexor pollicis longus (FPL) has evolved to be completely separate from FDP (51). Hence, most humans can flex the tip of the thumb without flexing the tip of the index finger. In many human hands, however, the FPL tendon nevertheless still sends a connection to the FDP tendon for the index finger, such that flexing the tip of the thumb also flexes the tip of the index finger (52).

### 4.2. Biomechanical Interactions in the Hand and Stabilizing Contractions

Newtonian mechanics dictate that because any given body part is connected to at least one other body part, the muscle contractions that produce motion of one body part will either move another body part by generating interaction torques at a connecting joint or will require stabilizing activity in additional muscles to check the other part’s motion. This principle was recognized by Bernstein, who stated, “…it is impossible for the internal muscles of a system to displace the center of gravity of the system. Because of this, if the internal muscles communicate an acceleration to a single link in one direction, another [attached] link will undergo compensatory acceleration in the opposite direction in a mechanically reactive manner.” (Ref. 53, p. 99). The notion that one part of the body, such as a single finger, can be moved without simultaneous active neural control of other body parts is a fallacy. The following three examples, described more than a century ago by simply palpating human muscles and tendons during active movements, illustrate such phenomena in the digits and wrist (54). In each example, note that the muscle contractions that move the intended digit result in biomechanical effects on another digit or the wrist as well, which are met by the nervous system with active contraction of additional muscles that check unwanted motion and stabilize those body parts. These stabilizing contractions occur concurrently with contraction of the prime mover and therefore presumably are based on the brain’s internal models of the hand’s biomechanics.

First, abduction of the index finger is produced by the first dorsal interosseous muscle (FDI). The lateral head of FDI originates from the thumb’s first metacarpal bone. Therefore, to prevent the thumb from moving toward the abducting index finger, muscles that act on the thumb, the abductor pollicis longus (APL) and extensor pollicis brevis (EPB), contract to stabilize the thumb (FIGURE 2, red arrow and red muscle shading). Individuated abduction of the index finger thus requires not only contraction of a muscle that acts on the index finger but also stabilizing contractions of muscles that act on the thumb.

Second, flexion of the tip of any finger can be produced only by contraction of that finger’s compartment of FDP, the belly of which lies in the forearm. The FDP tendons cross the wrist to reach the fingers, so contraction of any FDP compartment to flex a fingertip will tend to flex the wrist along with the finger. If the wrist is not to flex at the same time, extensors must contract to check flexion of the wrist.

In addition, third, humans abduct the little finger by contracting the muscle abductor digiti minimi (ADM). However, because ADM originates from the pisiform bone of the wrist, a wrist muscle, flexor carpi unlaris (FCU), also contracts to stabilize the pisiform. Contraction of FCU tends to produce ulnar deviation and flexion of the wrist; however, APL, a thumb muscle on the opposite side of the wrist, contracts as well to stabilize the wrist. Abducting the little finger thus engages stabilizing contractions in a wrist muscle and in a thumb muscle, which in turn causes some degree of movement in the thumb.

Because of biomechanical interactions such as these, no movement of any single digit produced by its prime movers occurs without concurrent contraction of additional muscles that check unintended motion of other digits and/or the wrist. In macaque monkeys, such stabilizing contractions have been recorded during individuated flexion and extension movements of the digits (55). In humans, stabilizing contractions in FDP have been recorded during isometric production of finger extension forces (56), as well as in EDC during intended contraction of FDP (57). These stabilizing contractions nevertheless result in some lesser degree of unintended motion or force production in noninstructed digits. Conversely, active contraction of muscles beyond the prime movers acting on a particular digit is required to achieve a high degree of individuation for that digit.

### 4.3. Multitendoned Muscles, Neuromuscular Compartments, and Motor Units

**FIGURE 3.**
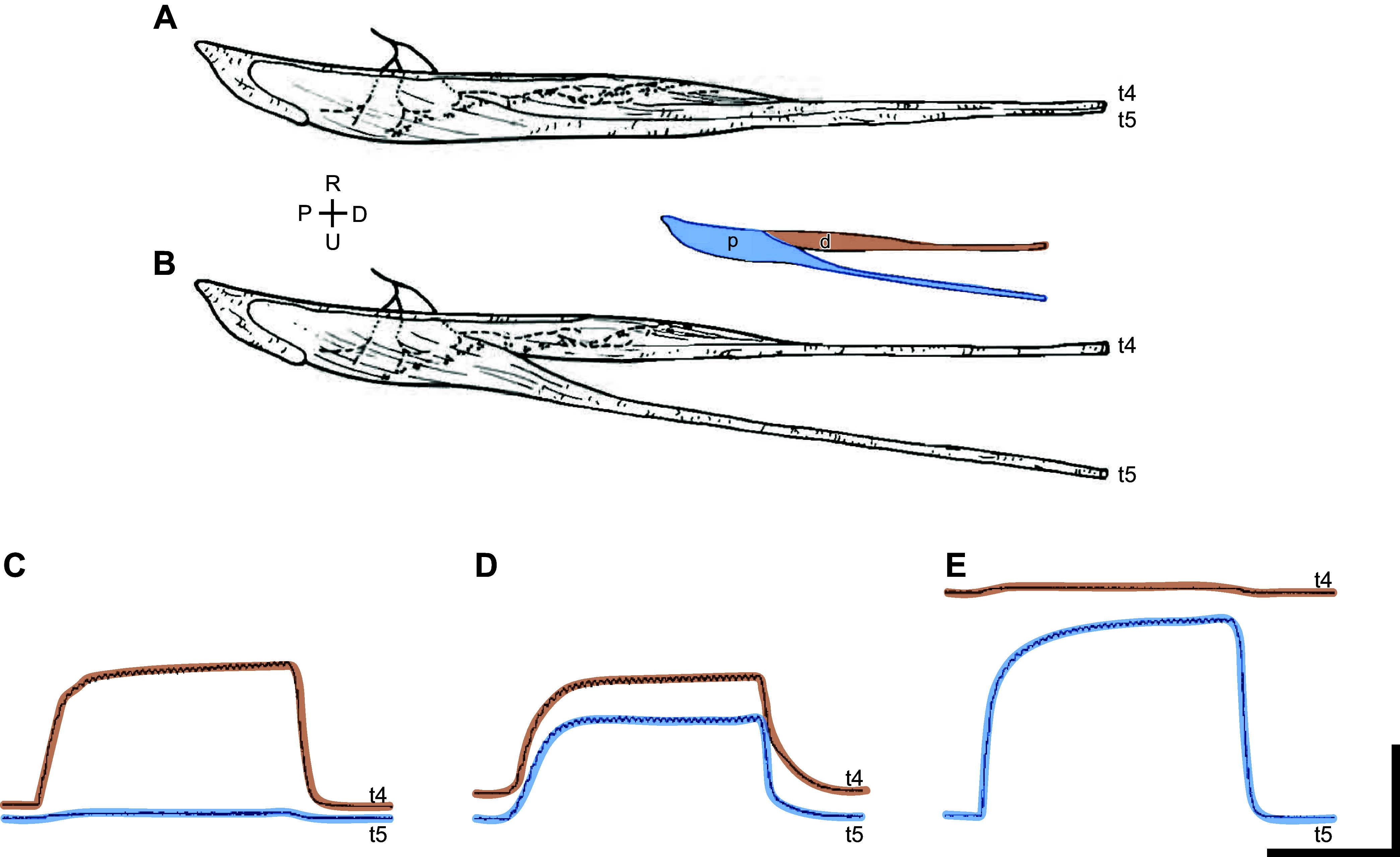
A multitendoned muscle and its motor units. *A*: the macaque ED45 sends tendons to the ring and little fingers, t4 and t5, respectively. In the forearm the 2 tendons lie adjacent to one another. *B*: the 2 tendons can be easily dissected from one another with no interconnections, suggesting that the muscle has 2 distinct compartments (*inset*), a distal compartment (d) acting on t4 and a proximal compartment (p) acting on t5. *C* and *E*: tetanic stimulation (40 Hz) of single motor units reveals that some SMUs (*C*) put tension selectively on t4, while others (*E*) put tension selectively on t5. *D*: still other SMUs, however, put tension simultaneously on both t4 and t5, indicating that the same motoneuron innervates some muscle fibers that insert on t4 and others that insert on t5. P, proximal; D, distal; R, radial; U, ulnar. Calibration bars: horizontal, 0.5 s; vertical, 0.2 g in *C*, 0.5 g in *D*, and 20 g in *E*. *A* and *B* were modified from Ref. 50, with permission from S. Karger AG, Basel; *C*–*E* were modified from Ref. 58, with permission from *Journal of Neuroscience*.

Many of the extrinsic finger muscles that flex and extend the digits send tendons to more than one digit. Comparing the gross anatomy of such muscles in macaque monkeys and humans provides additional insight into the greater human capacity for individuated finger movements. Rhesus monkeys and humans have a similar EDC that sends tendons to each of the four fingers with juncturae tendinum interconnecting the four tendons. In addition, both species have a separate extensor pollicis longus (EPL) that extends the thumb. Monkeys also have a multitendoned muscle that extends the index and middle fingers (extensor digitorum secundi et tertii, ED23), but in humans the homologous muscle (extensor indicis proprius, EIP) has lost its tendon to the middle finger, and extends only the index finger. Likewise, monkeys have a multitendoned muscle that extends the ring and little fingers (extensor digitorum quarti et quinti, ED45, FIGURE 3), whereas in humans the homolog [extensor digiti minimi (EDM)] has lost its tendon to the ring finger and extends only the little finger. These evolutionary differences enable humans to produce more highly individuated extension of the index and little fingers.

When a muscle sends tendons to multiple fingers, to what extent does it act as a single muscle that pulls on multiple fingers at the same time versus a number of separate neuromuscular compartments that each pull on a single digit? This depends not only on the structure of the tendons and the interconnections between them but also on the manner in which individual spinal motoneurons innervate the muscle fibers comprising their motor units. Although most motor units in a multitendoned muscle act on only the tendon to one digit, such is not always the case. For example, although the two tendons of the macaque ED45 are quite distinct (FIGURE 3*B*), many of the single motor units (SMUs) in ED45 put tension on both tendons simultaneously (FIGURE 3*D*) (58). When these motor units are recruited, they act to extend both digits 4 and 5. In contrast, in the human EDC, SMUs act relatively selectively on a single digit, indicating that substantially separate compartments of motor units are devoted to each finger (59).

Similar differences between macaques and humans are present in FDP. In macaques, FDP has two major compartments. Contraction of the radial compartment puts more tension on the index and middle fingers than on the ring and little fingers; the inverse is true of the ulnar compartment (60). Consistent with this pattern of tension distribution, monkeys voluntarily activate the radial compartment more when making an individuated flexion movement of the index or middle finger, and the ulnar compartment more when flexing the little or ring finger (55, 61). In comparison, the human FDP has more distinct compartments for each of the four different fingers. SMUs in the index, middle, ring, or little compartments generate force relatively selectively on the index, middle, ring, or little fingertip, respectively, although low amplitude forces often do appear on adjacent fingers (62). In particular, SMUs that generate force primarily on the little fingertip additionally generate about half as much force on the ring fingertip at the same time. (This is one reason humans have difficulty flexing the little fingertip without simultaneous flexion of the ring fingertip.) Although humans activate the four compartments of FDP relatively selectively during voluntary flexions of the different fingers, low-grade activation often occurs in a given compartment during flexion of adjacent digits as well (56). Nevertheless, humans have the ability to activate different compartments of FDP for each of the four fingers more selectively than macaques.

### 4.4. Divergent Last-Order Inputs to Multiple Spinal Motoneuron Pools

**FIGURE 4.**
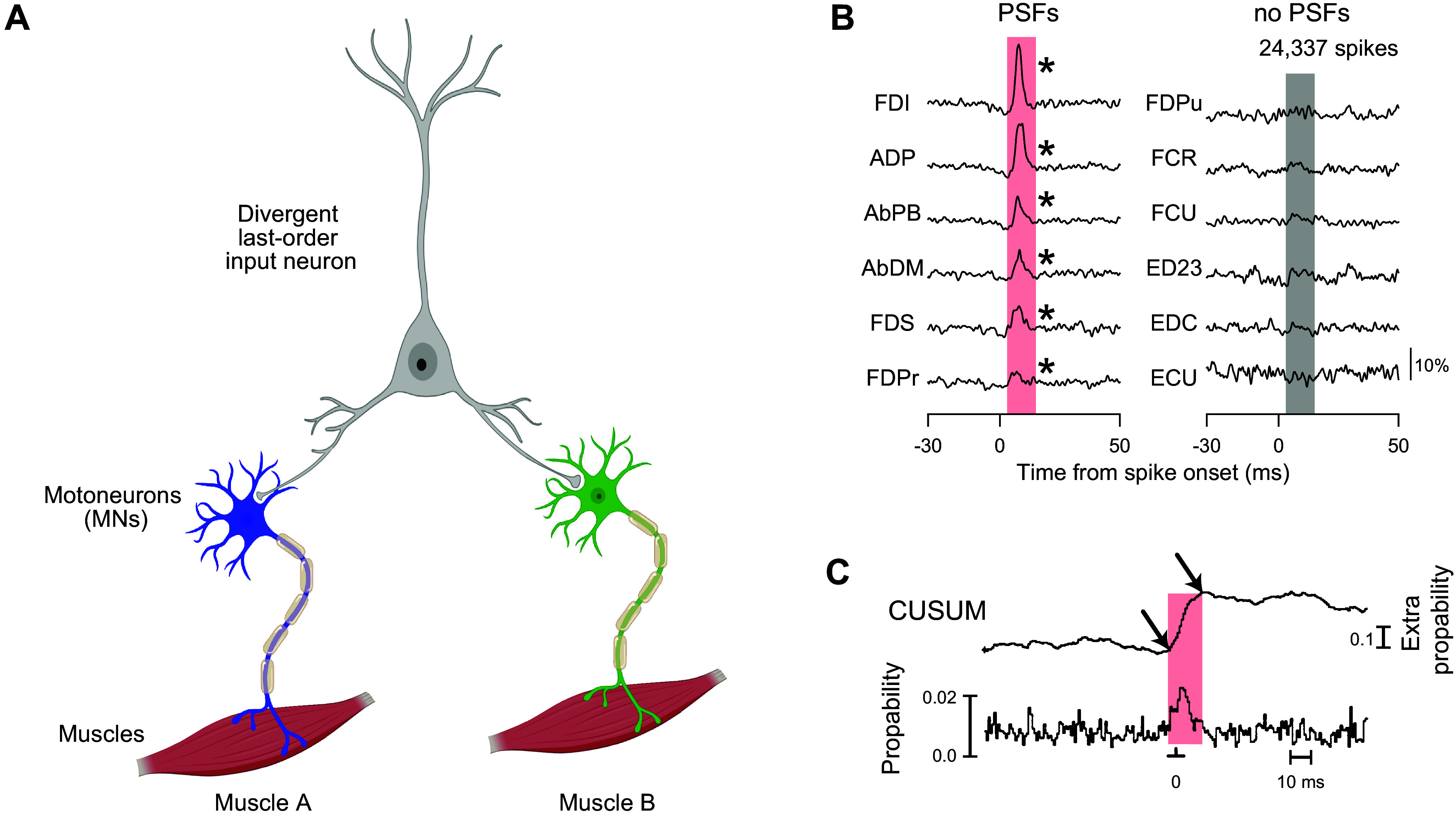
Two methods for identifying divergent last-order inputs to spinal motoneuron pools. *A*: diagram illustrating a last-order neuron that provides input to 2 motoneuron pools, each represented here as a single neuron, 1 blue and 1 green. *B*: spike-triggered averages (SpTAs) of the EMG activity of 12 macaque muscles that act on the fingers compiled with 24,337 segmental interneuron spike triggers aligned at time 0 ms. The 12 SpTAs have been segregated into 2 columns: those with PSEs [all postspike facilitations (PSFs*)] on the *left*, and those without postspike effects (PSEs) on the *right*. Image reproduced from Ref. 63, with permission from *Proceedings of the National Academy of Sciences U S A*. *C*: an example of short-term synchronization (STS) of 2 human motor units: one in the 3^rd^ and the other in the 4th dorsal interosseous muscles. The cumulative sum (CUSUM) helps identify the onset and offset of the peak in the correlogram below. Image reproduced from Ref. 64, with permission from *Journal of Physiology*. Transparent light-red rectangles in *B* and *C* emphasize significant effects. See glossary for additional definitions.

The spinal motoneuron pool driving any given muscle or neuromuscular compartment receives synaptic inputs from a number of sources in the central nervous system (CNS). In both monkeys and humans, many single neurons from any given CNS source provide last-order inputs that diverge to more than one muscle (FIGURE 4*A*). When such neurons discharge, they facilitate (and/or suppress) multiple muscles simultaneously.

In macaques, the muscles that receive last-order inputs from a single central neuron can be identified with an approach referred to as spike-triggered averaging of EMG activity (SpTA), a physiological tool for identifying the motoneuron pools of muscles and/or neuromuscular compartments that receive last-order inputs from a single central neuron (FIGURE 4*B*). The spikes discharged by the neuron are recorded simultaneously with EMG activity from multiple muscles. For each muscle, a snippet of EMG activity is clipped for tens of milliseconds before and after each spike discharged by the neuron; the snippets are then full-wave rectified, aligned at the time of the neuron’s spike (the trigger), and averaged. If the neuron has no synaptic input to the motoneuron pool, then the rectified, averaged EMG will show uniform variability. If the neuron produces excitatory postsynaptic potentials (EPSPs) in the motoneuron pool, however, the average will show a peak significantly larger than the preceding and following variability, reflecting the increased probability of motor unit action potentials occurring in the EMG at an appropriate latency following the neuron’s spikes (65–67). A narrow peak with appropriate latency indicates that the recorded neuron provided monosynaptic facilitation to the muscle’s motoneuron pool [postspike facilitation (PSF)], and a narrow trough indicates disynaptic inhibition through a segmental inhibitory interneuron [postspike suppression (PSS)]. Considered together, PSFs and PSSs may be termed postspike effects (PSEs). Wider peaks and troughs, often with an early onset latency, are referred to as synchrony facilitation or suppression. Synchrony effects indicate that the recorded neuron discharged many spikes nearly simultaneously with other neurons that provided input to the motoneuron pool, and the recorded neuron itself may not have provided any direct synaptic input to the motoneuron pool (68–70).

SpTA has demonstrated that last-order inputs to motoneurons arise from spinal segmental interneurons (71, 72), from the pontomedullary reticular formation (73), from the magnocellular red nucleus (74), and from the primary motor cortex (M1) (65). As discussed in detail for each of these centers in sects. 5 and 6, SpTA has shown that the outputs from single last-order input neurons often diverge to multiple muscles and/or neuromuscular compartments that act on different fingers. In the spinal cord, for example, single segmental interneurons active during precision pinch may provide inputs not only to thenar muscles (acting on the thumb) and the FDI (acting on the index finger) but also to both the radial and ulnar divisions of FDP (acting on all the digits) and the ADM (acting on the little finger) (63). Other illustrated examples also show that during voluntary wrist movements, some single neurons in the red nucleus (74) or in M1 (65) provide input to both ED23 and ED45.

In humans, recording single central neurons simultaneously with EMG activity from multiple muscles has yet to be performed. However, similar divergence to multiple muscles from single last-order input neurons has been examined by assessing short-term synchronization (STS) between pairs of SMUs (FIGURE 4*C*). Cross correlations between the discharge times of motor unit action potentials from two simultaneously recorded SMUs sometimes show peaks centered near time zero, when the trigger SMU fired its action potentials. Such STS between two SMUs indicates that the two spinal motoneurons both received synaptic input from the same, last-order, central neuron (75, 76). Although the exact location of that neuron cannot be identified in humans, studies in patients with CNS lesions suggest that most STS is produced by corticospinal neurons (77).

As one might expect, the strength and prevalence of STS are consistently greatest between motor units in the same muscle. However, such studies also provide evidence of last-order inputs that diverge to innervate the motoneuron pools of different muscles and/or neuromuscular compartments that act on different fingers. In humans, the dorsal interosseous muscles that abduct different fingers, the palmar interossei that adduct different fingers (78), the compartments of EDC that extend different fingers (79), the compartments of FDP (80) together with FPL (81), and the compartments of FDS (82) that flex different fingers, all have been found to receive divergent last-order inputs. Furthermore, last-order inputs diverge not only to innervate muscles of different groups acting on the same finger (such as the FDI and the EDC compartment acting on the index finger) but also to innervate muscles of different groups acting on different fingers, such as FDI and thumb extensors (78) or FDI and adductor pollicis (ADP) (83). The firing of central neurons that provide such divergent last-order inputs facilitates movement of multiple digits even though the human subject may intend to move only one.

Comparing the strength of STS among various human muscle pairings reveals three generalities relevant to the degree of individuation of the different digits (78). First, the greater the physical separation between two digits, the less STS is found between motor units acting on the two digits. Second, muscles acting on the more radial digits (i.e., the thumb and index finger) have less shared last-order input than muscles acting on the more ulnar digits (i.e., the middle, ring, and little fingers). Third, the finger flexors have less shared last-order input acting on different fingers than do the extensors or dorsal interossei. These three observations concerning STS parallel the behavioral observations described above in sect. 3 that *1*) during intended movement of any given digit more unintended motion or force tends to occur in adjacent digits than in nonadjacent digits; *2*) the thumb and index finger show a higher degree of individuation than the middle, ring, and little fingers; and *3*) the degree of individuation tends to be higher for flexion than for extension of any given digit (see, however, Ref. 82).

The presence of STS between motor units acting on different digits does not necessarily mean that the divergent inputs are numerous and powerful enough to recruit motor units acting on other fingers when a human subject intends to exert force on only one digit. Yet, such is the case. When human subjects exert a ramp of increasing force with one digit, motor units acting on that digit begin to discharge with little concurrent activity in motor units acting on other digits. However, as the force exerted by the intended digit increases, motor units acting on adjacent digits and then even on nonadjacent digits also begin to discharge. Recruitment of motor units acting on other digits occurs most readily in FPL and FDP, intermediate in EDC, and least readily in FDS (84–86). These findings provide direct evidence that enslaving results in part from central recruitment of motor units that act on unintended digits.

### 4.5. A Theoretical Speculation on Evolution of the Neuromuscular Apparatus for Individuation

**FIGURE 5.**
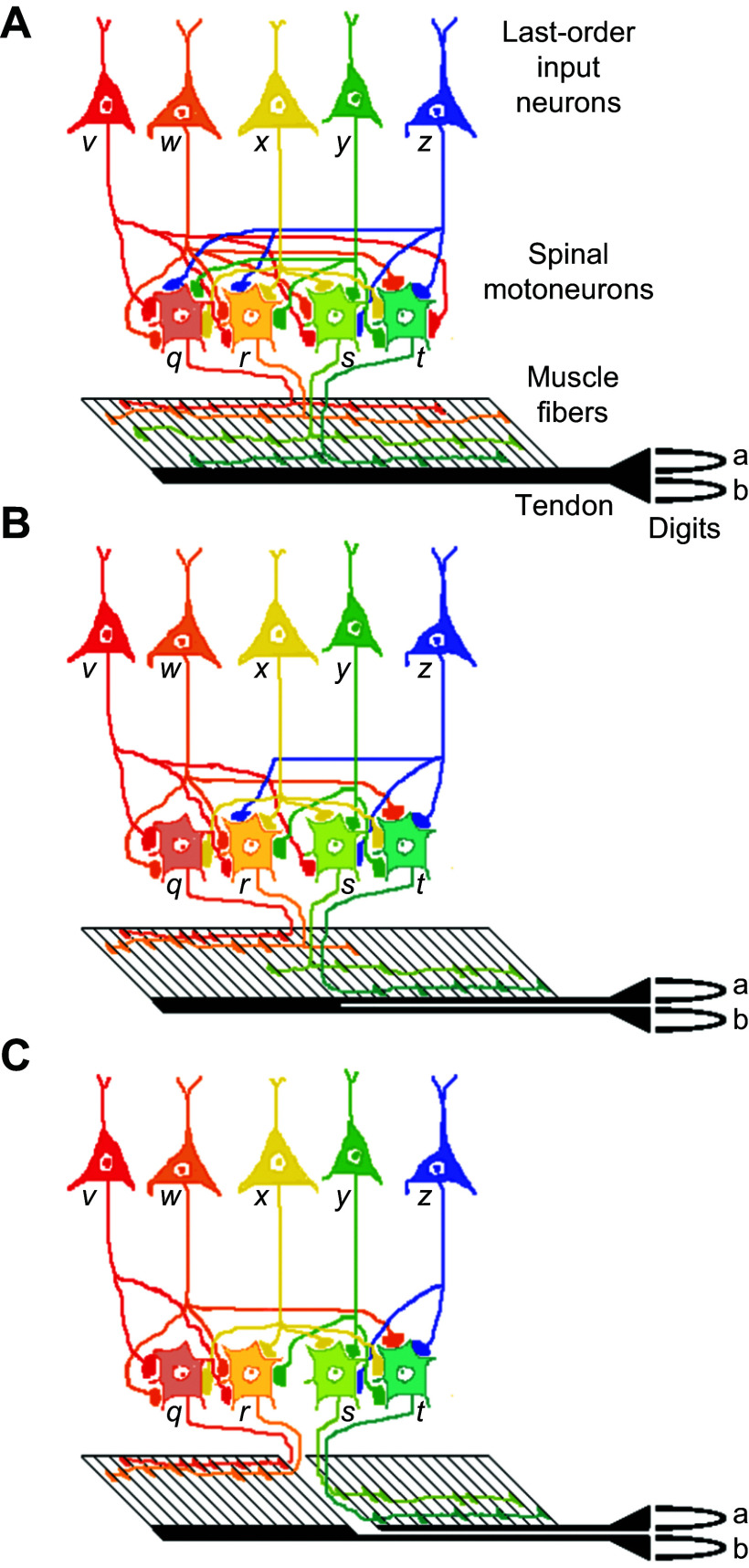
Theoretical neuromuscular evolution. *A*: a primitive muscle acted on 2 digits (*a* and *b*) equally. The muscle consisted of motor units distributed evenly through the muscle belly, all inserting on a single tendon. The spinal motoneurons (*q*, *r*, *s*, *t*) likewise were driven uniformly by last-order input neurons (*v*, *w*, *x*, *y*, *z*). *B*: muscle acting differentially on 2 digits. The single tendon has divided into a separate tendon for each digit. Some motor units act preferentially on one tendon or the other (*r*, *s*), while others act exclusively on a single tendon (*q*, *t*). Although some last-order inputs (*x*) still drive all four motoneurons, the others (*v*, *w*, *y*, *z*) drive motor units acting on only some of the motoneurons and thereby act preferentially (but not exclusively) on one digit or the other. *C*: 2 separate muscles or neuromuscular compartments. Motor units now exclusively innervate the portion of the muscle belly acting on the tendon to one digit or the other. Although some last-order inputs drive one group of motor units or the other exclusively (*v → q*, *r*; *z → s*, *t*), other last-order inputs drive more motor units on one side than on the other (*w*, *y*), and still others drive motor units on both sides equally (*x*). See the text for additional description. Image modified from Ref. 87, with permission from CRC Press.

Based on these observable differences in the gross anatomy of muscles in extant species, in the differential actions of the neuromuscular compartments of certain muscles, and in the divergence of last-order inputs to multiple spinal motoneuron pools, including descending inputs from M1, we can construct a theoretical picture of the neuromuscular evolution that created the capacity for individuated finger movements. For simplicity, we deal with only two digits rather than five. In our theoretical picture, a primitive muscle (FIGURE 5*A*) had a single tendon that acted on both digits (*a*, *b*). Individual spinal motoneurons (*q*, *r*, *s*, *t*) innervating the muscle each had endplates on muscle fibers spread through much of the muscle, and last-order input neurons (*v*, *w*, *x*, *y*, *z*) likewise made synapses on neurons throughout the spinal motoneuron pool. As these last-order input neurons became active, they facilitated the motoneuron pool relatively homogenously, and as the recruited motor units discharged, they exerted tension on the single tendon, moving the two digits in parallel. Although our theoretical primitive muscle acted on two digits, its peripheral innervation and descending control were that of a single muscle. When activated, this muscle, its spinal motoneuron pool, and its descending control could only move the two digits in parallel. In modern macaques, the ulnar compartment of FDP is such a muscle, acting to flex the ring and little fingers in parallel (60).

We then propose that natural selection favored the ability to move the two digits differentially, causing the system to evolve to the degree illustrated in FIGURE 5*B*. The tendon has become divided into two tendons that act on the two digits separately. The endplates of individual motoneurons are no longer spread so extensively and evenly through the muscle, such that now motor unit *q* exerts tension only on the tendon to digit *b*, and motor unit *t* exerts tension only on the tendon to digit *a*, but motor units *r* and *s* still exert some tension on both tendons. In modern macaques, ED45 (FIGURE 3) is such a muscle (58). Changes have occurred as well in the distribution of last-order inputs to the spinal motoneurons. Although neuron *x* still synapses on all four motoneurons, neurons *v* and *w* now each synapse on motoneurons *q* and *r*, which innervate the portion of the muscle acting primarily on digit *b*, but only on one of the two motoneurons (*s* or *t*) that innervate the portion acting primarily on digit *a*. The converse is the case for neurons *y* and *z*. Concurrent activity in all five last-order neurons still will move the two digits in parallel, but selective activity in neurons *v* and *w* will move digit *b* more than *a*, whereas selective activity in neurons *y* and *z* will move digit *a* more than *b*. This neuromuscular system now has the ability to act on digits *a* and *b* differentially, but not independently.

Finally, consider that evolution has favored still more highly individuated control of digits *a* and *b*, leading to the state illustrated in FIGURE 5*C*. Now, in addition to separate tendons, motoneurons *q* and *r* innervate only muscle fibers acting on the tendon to digit *b* whereas motoneurons *s* and *t* innervate only muscle fibers acting on digit *a*. By definition, these are now two separate muscles or distinct neuromuscular compartments within an anatomical muscle. Moreover, last-order input neuron *v* synapses only on motoneurons *q* and *r* acting on digit *b*, while neuron *z* synapses only on motoneurons *s* and *t* acting on digit *a*. Last-order input neurons that synapse on motoneurons serving only one muscle provide some ability for the system to act on the two digits independently. However, neuron *w*, while synapsing on both of the motoneurons acting on digit *b*, still also synapses on motoneuron *t*, which acts on digit *a*. The converse is true for neuron *y*. In addition, neuron *x* still synapses on all four motoneurons. These three neurons still provide last-order inputs that diverge to both motoneuron pools of what otherwise are two separate muscles or compartments. Activity of neurons *w*, *x*, and/or *y* will facilitate both muscles concurrently. As detailed in sects. 5 and 6, below, such shared last-order inputs to different muscles are common among macaque spinal interneurons, reticulospinal neurons, rubrospinal neurons, and even corticospinal neurons. Unless descending neuron *v* or *z* can be activated in isolation, some movement of digit *a* will occur when the subject intends to move digit *b* alone and vice versa. We view this as the state of FPL and FDP in modern humans: the FPL tendon has fully separated from FDP, and the two muscles can be activated relatively independently, but some shared last-order inputs to the two muscles still remain, as evidenced by STS between motor units in the two muscles (81).

## 5. SUBCORTICAL INPUTS TO SPINAL MOTONEURONS

**FIGURE 6.**
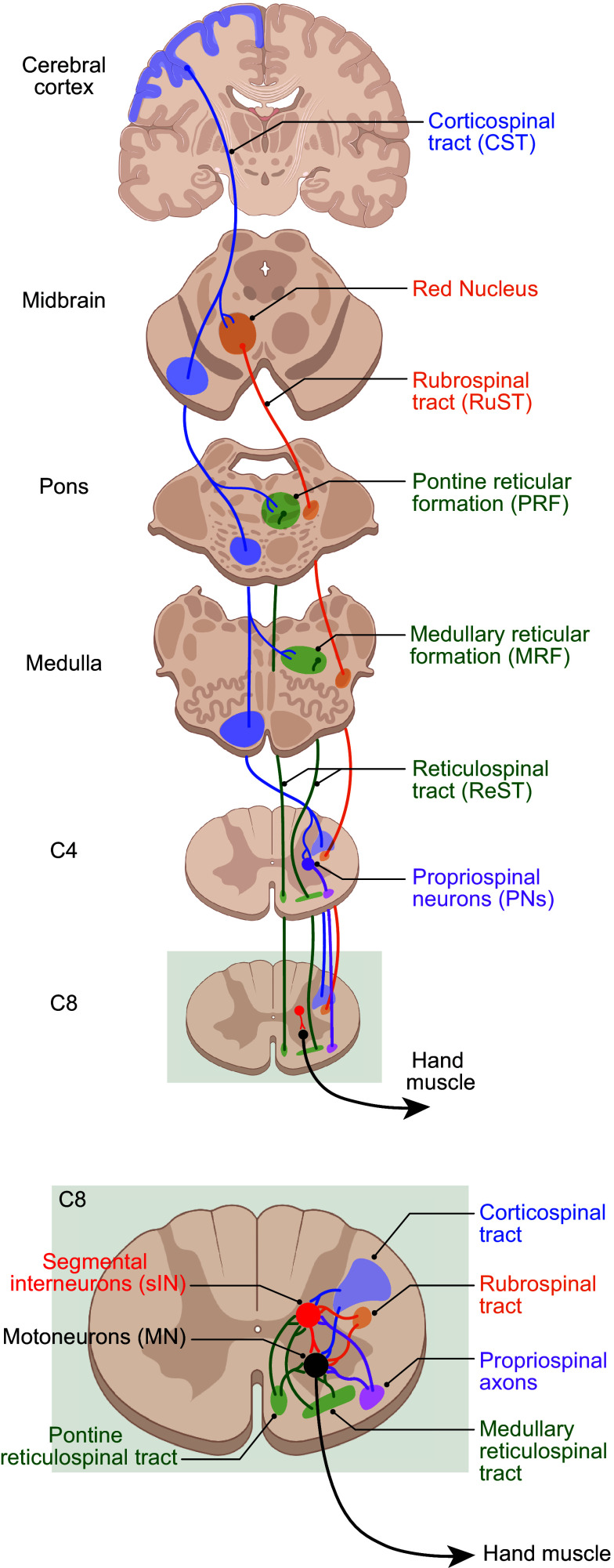
Descending pathways that provide synaptic input to spinal hand motoneurons. *A*: overview of descending pathways and their synaptic connections based on studies in macaque monkeys. (In humans the rubrospinal tract is thought not to descend past the C3-C4 level (90).) As each tract descends from its origin, it provides synaptic inputs to lower subcortical centers, creating multiple di- and oligosynaptic routes from the cerebral cortex to the spinal motoneurons that innervate hand muscles. Connections from the red nucleus to the reticular formation (91) and to propriospinal neurons (92), as well as from the reticular formation to propriospinal neurons (93), are not illustrated here, as their involvement in individuated finger movements is unknown. *B*: a zoomed-in view of the C8 level illustrating that these descending pathways all provide monosynaptic input not only to segmental interneurons (red) but also to the motoneurons (black) themselves.

Last-order inputs to spinal motoneurons come from a variety of sources in the central nervous system (2, 88, 89). These inputs have arisen as the vertebrate nervous system evolved from fish to mammals, to macaques, to humans. Inputs from spinal segmental interneurons (sINs), propriospinal neurons (PNs), neurons in the pontomedullary reticular formation (PMRF), and in the red nucleus, all converge on spinal motoneurons. In mammals, last-order inputs to spinal motoneurons in each of these subcortical regions in turn receive input from the cerebral cortex, providing multiple disynaptic and oligosynaptic pathways from the cortex to spinal motoneurons (FIGURE 6). In lower mammals including rodents, cats, aye-ayes, marmosets, and squirrel monkeys, these disynaptic pathways control the paw or hand movements used in behaviors such as grooming and food handling (9, 10, 12, 15, 94–98). In higher nonhuman primates, including capuchins, macaques, and apes, as well as in humans, these subcortical pathways also participate, together with monosynaptic inputs to spinal motoneurons that arise from the primary motor cortex, in generating more highly individuated finger movements, including precision pinch and within-hand manipulations.

### 5.1. Spinal Segmental Interneurons

Evolutionarily, the earliest inputs to spinal motoneurons were those from interneurons in the gray matter at every segmental level of the spinal cord. Spinal interneurons comprise the central pattern generators that produce swimming movements in fish, which evolved to be used for quadruped locomotion and to mediate a variety of spinal reflexes such as scratching (3, 99, 100). Some of these interneurons evolved further to participate in voluntary limb movements.

In macaque monkeys, segmental interneurons produce PSEs in the wrist, extrinsic finger, and/or intrinsic hand muscles. During isometric flexion/extension force production at the wrist, segmental interneurons produce PSFs in extrinsic finger muscles, as well as wrist muscles, and have a larger proportion of PSEs in flexor (58%) than extensor (29%) forearm muscles as compared to other sources of input (71). Segmental interneurons are active during the grasp phase of reach-to-grasp movements (101). During precision grip, segmental interneurons produce PSEs (by and large PSFs) more commonly in intrinsic and extrinsic finger muscles than in wrist or elbow muscles, with individual interneurons producing PSEs in 2.5 finger muscles on average (72, 102). Groups of segmental interneurons that produce PSF of a similar set of muscles may constitute specialized synergies for performing precision grip (63).

### 5.2. C3-C4 Propriospinal Neurons

A group of neurons with somata at the C3-C4 levels of the spinal cord also plays a role in precision grip. In cats, these C3-C4 propriospinal neurons have been shown to receive descending input from the motor cortex, red nucleus, and PMRF and project their axons down to spinal segments C5 through T1, where the motoneurons of the forelimb muscles are located (5). C3-C4 propriospinal interneurons influence motoneurons through both excitatory and inhibitory monosynaptic connections. In cats, this system participates more in reaching a target than in taking the object with the paw. In macaques, however, a lesion of the lateral columns at the C2 level, which eliminates corticospinal (and rubrospinal) input to both the C3-C4 propriospinal neurons and lower cervical levels, abolishes precision grip (103). However, following a similar lesion at the C4/C5 border, which eliminates corticospinal (and rubrospinal) input to the lower cervical cord but not to the C3-C4 propriospinal neurons, macaques with training may recover some ability to retrieve food morsels with an altered grasp between the index finger and thumb (104, 105) (see sect. 8). Moreover, selective reversible inactivation of these propriospinal neurons transiently impairs precision pinch (106). C3-C4 propriospinal neurons thus normally participate in generating precision pinch.

In humans, putative propriospinal neurons likewise transmit corticospinal excitation to hand and finger motoneurons, while receiving both proprioceptive afferent input from other muscles and cutaneous afferent input. The propriospinal system thus may play a role in rapidly updating the descending input to motoneurons based on incoming somatosensory feedback, for example, arresting movement when the limb contacts an object (107). Interestingly, however, although the human C3-C4 propriospinal system projects to motoneurons of the extrinsic finger muscles such as FDS and EDC, these propriospinal connections do not appear to reach intrinsic hand muscles such as FDI, abductor pollicis brevis (APB), or opponens pollicis (OP) (108), which may limit their role in individuated finger movements.

### 5.3. Reticulospinal Neurons

Neurons of the PMRF project to spinal motoneurons through the reticulospinal tract (ReST). ReST neurons are present in the most primitive fish where they mediate rapid responses to external stimuli. In mammals ReST neurons traditionally have been thought to participate primarily in control of posture and locomotion (109). In primates, the PMRF receives input descending from the premotor, supplementary motor, and primary motor cortex, including collaterals from descending corticospinal axons (110, 111). The ReST then descends in the ventral (or anterior) column of the spinal cord to terminate in both the cervical and lumbar enlargements (112, 113). Recently these projections in macaques have been shown to include monosynaptic excitatory inputs not only to segmental interneurons (101) but also to motoneurons innervating forearm and intrinsic hand muscles (114), converging with corticospinal inputs in both types of neurons. Moreover, reticulospinal neurons have been found to be active both during arm movements (115) and during index finger movements (116). In contradistinction to the rubrospinal and corticospinal projections discussed below, many reticulospinal neurons project to both sides of the spinal cord, with some axons crossing in the brainstem and others sending axon collaterals across the midline in the commissural spinal gray matter (117–119). Electrical microstimulation in the reticular formation evokes contractions in both contralateral and ipsilateral arm muscles (120). In addition, individual ReST neurons produce PSEs bilaterally, tending to be reciprocal across the midline: facilitating ipsilateral flexors and contralateral extensors, while suppressing ipsilateral extensors and contralateral flexors (73). Moreover, most ReST neurons receive cortico-reticular inputs converging from multiple sites in M1 and from the supplementary motor area bilaterally (121). Nevertheless, microstimulation at multiple sites in the reticular formation evokes patterns of muscle activity, which, while potentially accounting for much of natural muscle activity, accounts less well for nuanced, finely fractionated movements than patterns of muscle activity evoked by microstimulation at multiple sites in M1 (122).

In humans, the reticulospinal system is thought to be activated by sudden loud sounds, thereby mediating the acoustic startle response (123). Although acoustic startle can shorten the reaction time of planned voluntary movements, the effect appears primarily in power grasp rather than precision grip or other highly individuated finger movements (124, 125).

### 5.4. Rubrospinal Neurons

The magnocellular red nucleus in the midbrain and its descending rubrospinal tract (RuST) evolved in parallel with the emergence of lateral appendages, such as the wings of rays or the fore- and hind-limbs of quadrupeds (126). In macaques, the red nucleus receives input from the primary motor cortex (127). The descending RuST crosses the midline in the brainstem, runs in the ventrolateral part of the medulla, and mingles with the descending corticospinal tract in the lateral column of the spinal cord before terminating in ventral horn of the spinal cord on segmental interneurons and motoneurons. Many neurons in the magnocellular red nucleus produce PSEs in the wrist and extrinsic finger muscles of the contralateral upper extremity (74). On average, the outputs of such rubromotoneuronal (RM) cells diverge to three of six muscles tested, with more than twice as many RM cells facilitating forearm extensors as flexors (a stronger extensor predominance than that found for cortico-motoneuronal cells described in sect. 6). A few RM cells also produce disynaptic PSS, more often in flexors than extensors. In humans, however, the magnocellular red nucleus is comparatively small, and the RuST does not descend below the upper cervical segments (90). One might speculate that in humans the RuST terminates on C3-C4 propriospinal neurons. The activity of RM cells, however, has yet to be examined during individuated finger movements.

## 6. THE PRIMARY MOTOR CORTEX HAND REPRESENTATION: ENGINE OF INDIVIDUATION

### 6.1. The Role of the Primary Motor Cortex in Individuated Movements

**FIGURE 7.**
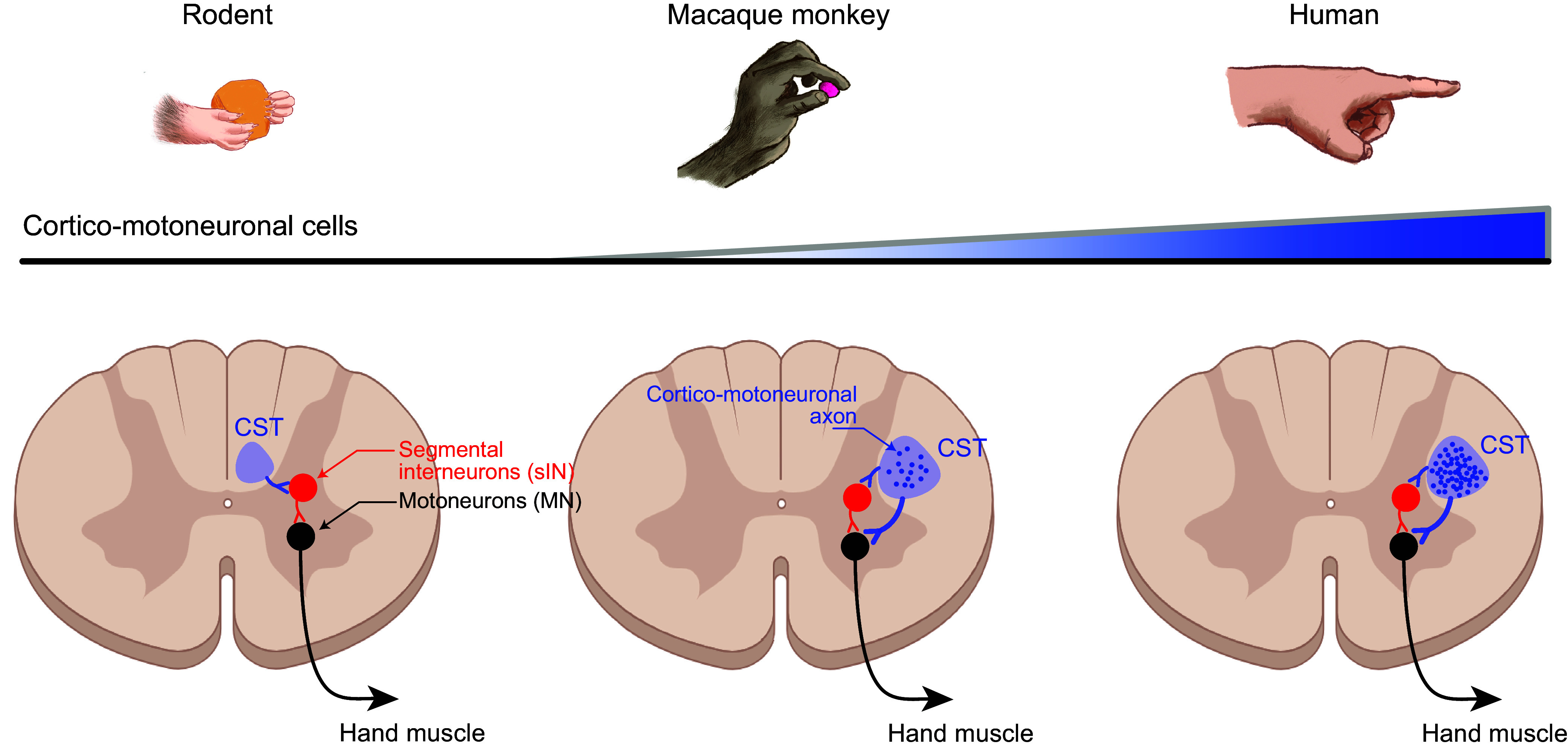
Species differences in the corticospinal tract and number of cortico-motoneuronal cells (CM cells). The evolutionary progression of individuated finger movements from rodents to macaques to humans correlates with changes in the corticospinal tract (CST). The position of the CST in the spinal cord shifts from the ventralmost portion of the dorsal column in rodents to the dorsal portion of the lateral column in carnivores and primates. The number of descending corticospinal axons increases. Rather than synapsing only on segmental interneurons (red), in macaques and then humans increasing numbers of CST axons synapse directly on spinal motoneurons (black), forming monosynaptic CM connections.

Whereas all the subcortical centers that provide inputs to spinal motoneurons are present in submammalian species, the cerebral cortex evolved only in mammals. In rodents such as mice and rats, the projection from the cerebral cortex to the spinal cord, or corticospinal tract (CST), descends largely in the dorsal column contralateral to the hemisphere of origin and terminates largely in the dorsal horn as well as the intermediate layers of the spinal gray matter (128, 129) (FIGURE 7). However, in carnivores, macaques, apes, and humans, the CST descends largely in the dorsolateral column and terminates heavily in the ventral horn (88). The number of corticospinal axons increases from rodents to carnivores, to monkeys, to apes, to humans (Ref. 130, p. 63). Along the mammalian evolutionary scale, the proximity of CST terminations to spinal motoneurons correlates roughly with dexterity (94, 131). The deficits that result from damage to M1 or the CST further attest to the primacy of this system in generating individuated movements (see sect. 8).

**FIGURE 8.**
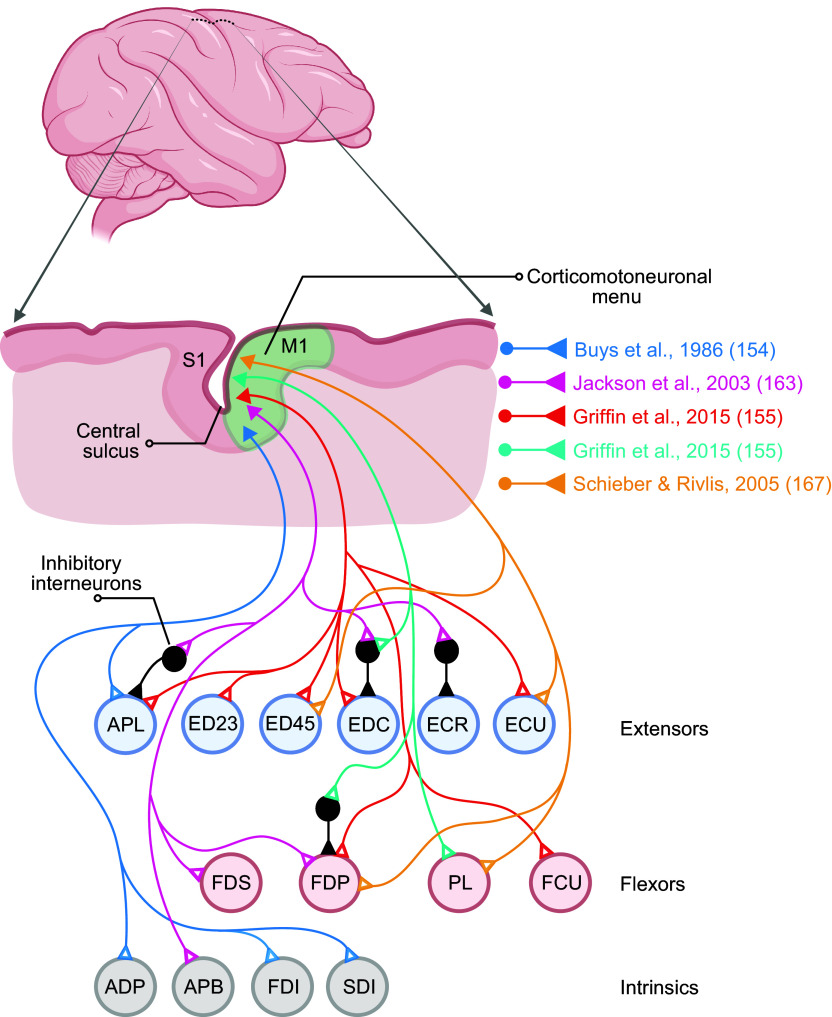
Divergent last-order inputs from cortico-motoneuronal (CM) cells to spinal motoneuron pools. Neurons in macaque M1 are illustrated sending monosynaptic excitatory connections that diverge to the spinal motoneuron pools of multiple finger muscles, as well as to segmental inhibitory interneurons (black) that produce disynaptic inhibition in motoneuron pools. Image adapted from Ref. 139, with permission from *Journal of Neurophysiology*, using specific examples of CM cells and their connections to spinal motoneuron pools documented with spike-triggered averaging in the publications cited in the color legend. See glossary for additional definitions.

Although ∼35% of the macaque CST arises from M1 (Brodmann’s area 4), another ∼35% of CST axons arise from the more anterior cortical motor areas that are subdivisions of area 6, 23, and 24, including the dorsal and ventral premotor cortex, supplementary motor area, and the cingulate motor areas in the frontal lobe, with the remaining ∼30% coming from areas 3a, 3 b, 1, 2, 5, and SII in the parietal lobe and insula (132–138). Macaque M1, at the mediolateral level of the upper extremity representation, occupies the anterior bank of the central sulcus and the posterior half of the precentral gyrus (FIGURE 8), with area 6 lying immediately anterior. In humans, Brodmann’s original map showed a similar location of the boundary between areas 4 and 6. Importantly, however, modern reappraisal found that at the level of the hand knob (140), where the medial and lateral limbs of the central sulcus meet, the human area 4/6 boundary lies entirely within the anterior bank of the central sulcus, such that the surface of the human precentral gyrus at this mediolateral level is entirely area 6 (141).

Cortical neurons that make monosynaptic connections to spinal motoneurons, corticomotoneuronal (CM) cells, are absent in carnivores and sparse in new-world monkeys, with the exception of capuchins (94). However, in macaque monkeys, CM cells constitute as much as 55% of the CST arising from M1 (67, 142). This fraction presumably is higher in apes and higher still in humans (143, 144). In macaques, the somata of CM cells are localized almost entirely in an evolutionarily “new,” caudal portion of M1, lying in the anterior bank of the central sulcus (Ref. 145; see, however, Ref. 146). Although the exact location of CM-cell somata in humans is unknown, humans also have distinguishable anterior and posterior divisions of M1 (147). Via CM cells, M1 directly influences spinal motoneurons to individuate more sophisticated movements from older, less varied, relatively stereotyped, synergies.

The corticofugal output of macaque M1 involves not only CM-cell projections to spinal motoneurons and other CST projections to spinal segmental interneurons but also descending projections to the C3-C4 propriospinal neurons, pontomedullary reticular formation, and red nucleus, each of which in turn provides a descending projection to spinal segmental interneurons and to spinal motoneurons (89) (FIGURE 6). In our evolutionary view, the newer descending input from M1, especially the CM-cell input from new M1, has been added on top of these older descending systems to enhance control of voluntary movements. In addition to providing another level of input to spinal motoneurons, this newer control from M1 facilitates and suppresses selected outputs from the older descending systems, sculpting the total output to individuate movement (122, 148, 149). The primary motor cortex acting via the CST, especially via CM cells, thus might be considered the “engine of individuation.”

### 6.2. Distributed Organization Within the M1 Hand Representation

At the microscopic level, M1 individuates movement through a distributed organization. Studies in macaques have shown that the cortical projection to the spinal motoneuron pool of any given muscle is distributed over a relatively wide territory in Brodmann’s area 4 (150, 151). The M1 territory projecting to any given muscle therefore necessarily overlaps extensively with the territories projecting to multiple other neighboring and related muscles. Moreover, horizontal axon collaterals from any given locus within the area 4 upper extremity representation project throughout the upper extremity representation (152). Any given finger muscle thus receives inputs converging from much if not all of the upper extremity representation.

Conversely, single CM cells in layer V often project to multiple muscles, which has been shown both anatomically and physiologically. In cynomologous monkeys, collaterals from a single, horseradish peroxidase-filled, M1 corticospinal axon have been traced into the spinal motoneuron pools of up to 4 different muscles (153). In rhesus monkeys, ∼60% of precision-pinch-related pyramidal tract neurons produce PSF (55%) and/or PSS (7%) in at least one forearm or intrinsic hand muscle (67). These CM cells produced PSEs in an average of 1.4 muscles when 5 muscles were sampled, but in an average of 2.0 muscles when 10 muscles were sampled (154). About 27% of wrist-related M1 neurons produced PSEs in at least one of five to six sampled forearm muscles, on average in more than two muscles, with more of these CM cells facilitating the extrinsic finger extensors than flexors (65). In addition, when 22–24 muscles throughout the upper extremity were sampled during a food retrieval task, ∼71% of CM cells projected to 2 or more muscles, an average of ∼3 muscles receiving input from a single CM cell (69). Many single CM cells thus have outputs that diverge to multiple muscles.^1^

Two points regarding the divergence of single CM-cell output to particular muscles are especially relevant to individuated finger movements. First, single CM cells that innervate the spinal motoneuron pools of one or more finger muscles may also innervate muscles acting across more proximal upper extremity joints, including the wrist, elbow, and/or shoulder (65, 69, 70, 155). Second, as illustrated in FIGURE 8, single CM cells can innervate the spinal motoneuron pools of muscles acting on more than one digit. Single CM cells have been illustrated that produce PSF in APB and FDI (acting on digits 1 and 2) (69); in APB, FDI, and the second dorsal interosseous (SDI) (digits 1–3) (154); in ED23, ED45, and EDC (digits 2–5) (65); and in APL, FDP, ED23, ED45, and EDC (digits 1–5) (155). When such CM cells discharge, they facilitate movement of (and/or force development in) multiple digits simultaneously.

Because of *1*) the convergence of inputs to any given muscle from a wide territory in M1, *2*) the horizontal intracortical interconnections throughout the M1 upper extremity representation, and *3*) the divergence of outputs from single CM cells to multiple muscles (distal and proximal, flexors and extensors, acting on multiple digits), neurons throughout the M1 hand representation are active during any given individuated finger or wrist movement. In rhesus monkeys, single neuron activity during individuated flexion or extension of each of the five digits or the wrist is distributed throughout the same territory in M1, with little if any spatial segregation of the activity center of mass for movements of different digits (156). In humans, functional magnetic resonance imaging (fMRI) shows activity throughout the same territory during movement of any given digit (157, 158), though contrasting the activity during movements of different fingers does show a mediolateral somatotopic progression of more intense activation from little finger to thumb movements (159, 160).

In addition to the wide spatial distribution of activity in M1 during any given individuated finger movement, single macaque M1 neurons in general and single CM cells in particular discharge for a variety of individuated movements of different digits (70, 161). This may include discharge during both flexion and extension movements of the same digit, as well as discharge during movements of nonadjacent digits (e.g., flexion of the thumb or middle finger but not the index finger). Moreover, although small groups of neurons especially active for one particular finger or wrist movement may be present, such groups are found for only a minority of movements and not for each movement, and those groups that are present vary from monkey to monkey.

### 6.3. Complexities of CM-Cell Activity

CM cells might seem most likely to discharge when all their target muscles need to be activated concurrently. Activity of a group of CM cells each with outputs diverging to the same set of muscles then could be viewed as a synergy (162). Indeed in monkeys performing precision pinch, the presence of synchronizing inputs to CM cells with shared target muscles was more prevalent than synchronizing inputs to CM cells with different target muscles (163). This indicates that CM cells with shared sets of target muscles tend to be activated concurrently during precision pinch. In addition, in monkeys generating isometric ramp-and-hold wrist torques, synchrony indicative of common inputs was stronger between CM cells that were activated concurrently (164, 165). These findings might suggest that synchronization of large numbers of CM cells creates a synergy of their shared target muscles. However, the PSEs produced by each of a pair of synchronized CM cells showed little evidence of synchronization among large numbers of last-order inputs to the spinal motoneuron pools, i.e., synchrony PSEs. Moreover, the PSE produced by each member of a synchronized CM-cell pair was found to be independent of the PSE produced by the other. Together these observations suggest that large numbers of CM cells do not tend to become synchronized. The prevalence of synchronization between M1 neurons that do produce synchrony PSEs in muscle activity has yet to be quantified, but available evidence thus suggests that fixed synergies of multiple muscles are not often created by large pools of CM cells.

Increasing the variety of movements reveals more about the complexity of CM-cell activity. For example, during wrist movements in eight different directions made with the forearm pronated, supinated, or in between, different CM cells facilitated a given wrist muscle depending on whether the mechanical situation required that muscle to function as an agonist, an antagonist, or a stabilizer (155). However, a given CM cell did not necessarily discharge for only one of these functions, indicating that individual CM cells can serve different functions during different movements. Likewise during a reach-to-grasp food-retrieval task, although the varied activity patterns of three to five CM cells could be combined to predict much of the EMG activity in one shared target muscle, the activity of a given CM cell was not necessarily highly correlated with the activity of each target muscle it facilitated (166).

**FIGURE 9.**
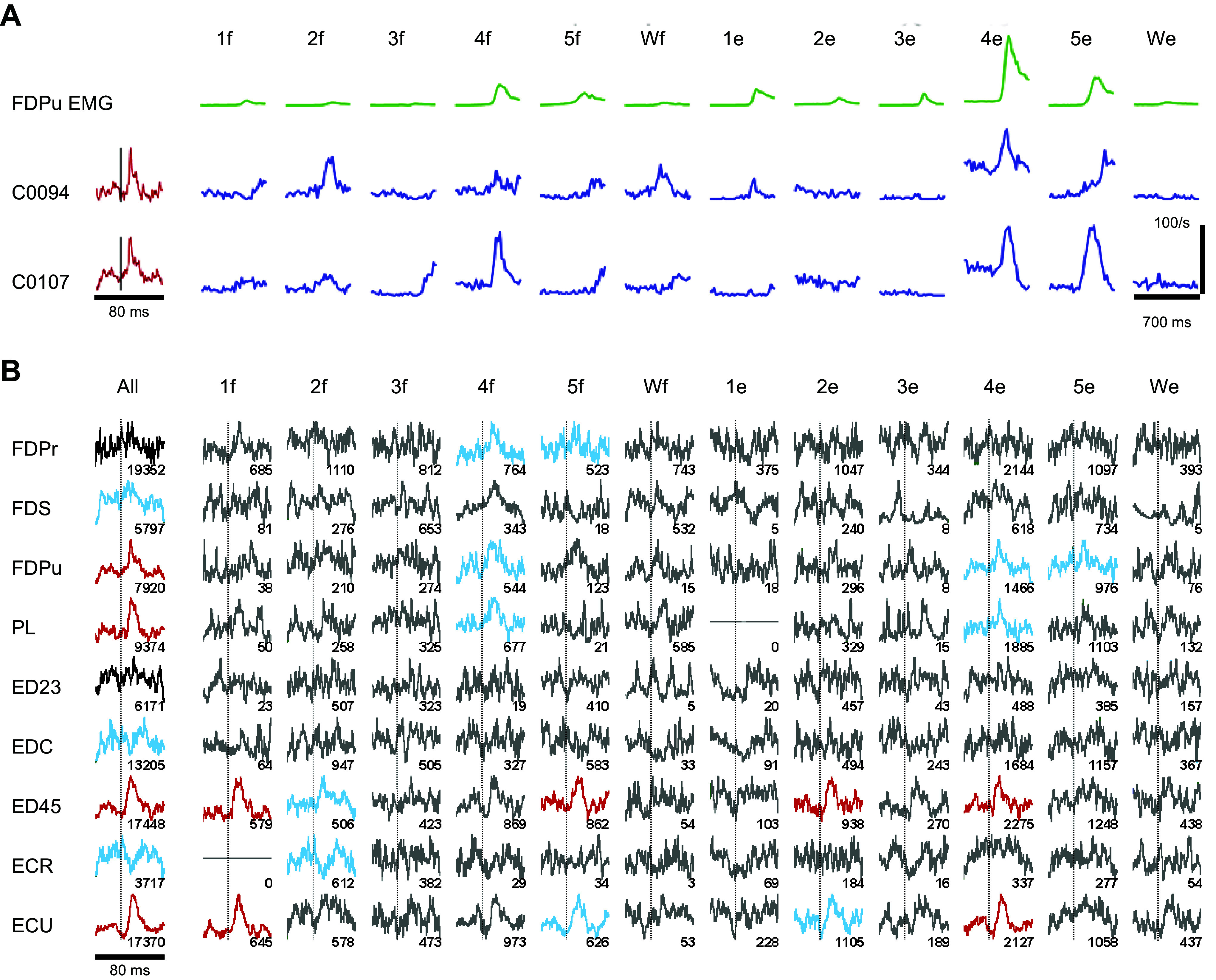
Cortico-motoneuronal (CM) cells and target muscles. *A*, *column 1*: spike-triggered averages (SpTAs; red) produced by 2 CM cells (C0094 and C0107) in the EMG activity of FDPu. *A*, *columns 2–13*: multiple-trial averages of rectified EMG activity in FDPu (green), and of firing rate in each of the 2 CM cells (blue), during each of 12 individuated finger and wrist movements. Different movements are designated by the number of the digit (1 = thumb through 5 = little finger; W = wrist) and the direction of movement (f = flexion; e = extension). Note that the time base of the SpTAs is 80 ms, 30 ms before and 50 ms after the aligned spike times (vertical line); whereas the time base of the averaged EMG and firing rate traces is 700 ms, 500 ms before, and 200 ms after the end of each movement. Vertical scale of 100 spikes/s applies to the averaged CM-cell firing rates during all 12 movements for both CM cells. Vertical scale of FDPu EMG and SpTAs is in arbitrary units. Image adapted from Ref. 167, with permission from *Journal of Neurophysiology*. *B*: each column shows SpTAs of rectified EMG from each of 9 muscles (rows) triggered from spikes discharged by CM cell C0107. SpTAs in *column 1* (All) incorporate spikes discharged throughout the recording session. SpTAs in the remaining columns incorporate spikes discharged only during trials of a particular movement. Snippets of EMG activity were included in each SpTA only if some EMG activity was present above the noise level in the 80 ms around the spike time. Numbers below each trace indicate the number of snippets included in that SpTA. All SpTAs have been scaled to fill the same vertical height from their minimal to maximal values. The time base of each SpTA is 80 ms, with the trigger time indicated by a vertical line in each column. SpTAs with highly significant effects (*P* < 0.0001) are shown in red; those with effects of intermediate significance (0.0001 ≤ *P* < 0.05) in cyan; those with no significant effect (0.05 ≤ *P*) are black if ≥4,000 snippets with EMG activity were available, grey if <4,000. Image reproduced from Ref. 168, with permission from *Journal of Physiology*. See glossary for additional definitions.

Similar observations have been made in monkeys performing 12 individuated finger and wrist movements (167). FIGURE 9*A* illustrates two CM cells, C0094 and C0107, each of which produced a strong PSF in a shared target muscle, the ulnar compartment of FDP (FDPu, red traces at *left*). Although FDPu and both CM cells all discharged most intensely during 4e (when FDPu acted as an antagonist), across the 12 movements the activity pattern of neither CM cell (blue traces) closely matched that of FDPu (green traces). Nor did the activity of one CM cell match that of the other. C0107 discharged more intensely than C0094 during 4f and 5e, when FDPu was moderately active, whereas C0094’s discharge was stronger during 2f and Wf, when FDPu was inactive. We can infer that the two CM cells were activated to varying degrees with different sets of other neurons for performing these different individuated finger and wrist movements.

FIGURE 9*B* further illustrates the complexities of C0107’s activity (168). Here, spike-triggered averages for each of 9 simultaneously recorded muscles were formed first using the spikes recorded during all 12 individuated finger and wrist movements together (All) and then using spikes from each of the 12 movements separately (1f through We). Using the spikes from all the movements showed that C0107 produced highly significant (red) PSFs not only in FDPu, but also in PL, ED45, and extensor carpi radialis (ECU), as well as moderately significant (cyan) PSFs in FDS and PSSs in EDC and ECR. Breaking out these effects by movement showed that during 4e, when C0107 was most active (FIGURE 9*A*), it facilitated FDPu, PL, ED45, and ECU. However, during 5e, when C0107 was also quite active, it produced only a moderate PSF in FDPu. Such observations indicate that during individuated finger movements a given CM cell cannot be characterized as controlling any single muscle, synergistic group of muscles, movement, or performing any fixed function. Rather, CM cells function collectively as a network.

How can a CM cell facilitate (or suppress) activity of a muscle at some times and not at others? A spinal motoneuron receives an estimated 50,000 synapses (169). In a hypothetical pool of 100 motoneurons, a single CM cell making a hypothetical 200 monosynaptic connections would provide only 0.004% of the 5,000,000 synapses. Consequently, EPSPs from that CM cell can only increase the probability of discharge in any given motoneuron if that motoneuron’s membrane potential already is close to threshold for the 5- to 10-ms duration of the CM-cell’s EPSP (65, 76).^2^ If the motoneuron’s membrane potential is not close to threshold, because of either insufficient excitation or sufficient inhibition from other inputs, then that CM-cell EPSP will have no effect on the motoneuron’s discharge. The throughput of CM-cell activity to EMG activity in any given target muscle therefore can change dramatically depending on the activity of other last-order inputs to the motoneuron pool. For example, the strength of a CM cells’ effect on a given muscle can change between the movement versus hold phase of a precision pinch (67), between a power grip versus a precision pinch (154), or depending on whether the monkey is rewarded for activating one muscle versus another concurrently with the CM cell (170).

### 6.4. Intracortical Inhibition Minimizing Unintended Finger Movements

In producing a particular individuated finger movement, besides the activation of selected CM cells and other CST neurons that facilitate the necessary muscle activity, inhibitory interneurons within M1 play a role in focusing M1 activity to minimize unintended movements. When human subjects flex the index finger, for example, the muscle FDI contracts as an agonist. Other muscles not involved in flexing the index finger, such as APB or ADM, may contract as well. A single pulse of transcranial magnetic stimulation (TMS) delivered at the start of the FDI contraction produces a larger motor-evoked potential (MEP) in FDI than the same test pulse delivered at rest, reflecting the increased cortical facilitation of FDI that contributes to index finger flexion. The same TMS pulse may produce an MEP in APB or ADM smaller than that obtained at rest, indicating enhanced intracortical inhibition of these muscles that do not act on the index finger, a mechanism sometimes referred to as “surround” inhibition (171, 172).

Such intracortical inhibition of activity in uninvolved muscles begins prior to the contraction of FDI and can be stronger and begin earlier when the subject has to choose between flexing the right versus left index finger (173). The phenomenon may be more prominent in the dominant left hemisphere of right-handed subjects than in the right hemisphere (174). Note, however, that intracortical inhibition of activity in uninvolved muscles appears in some normal human subjects but not in others. The presence or absence of intracortical suppression is associated with differences in the distribution of cortical potentials evoked by the TMS pulses (175). Understanding the conditions under which intracortical inhibition contributes to individuation of finger movements in humans requires further study. We speculate that differences among healthy subjects may reflect differences in their prior experience and training.

### 6.5. A Schematic Model of M1: The Cortical Hyper-Piano

**FIGURE 10.**
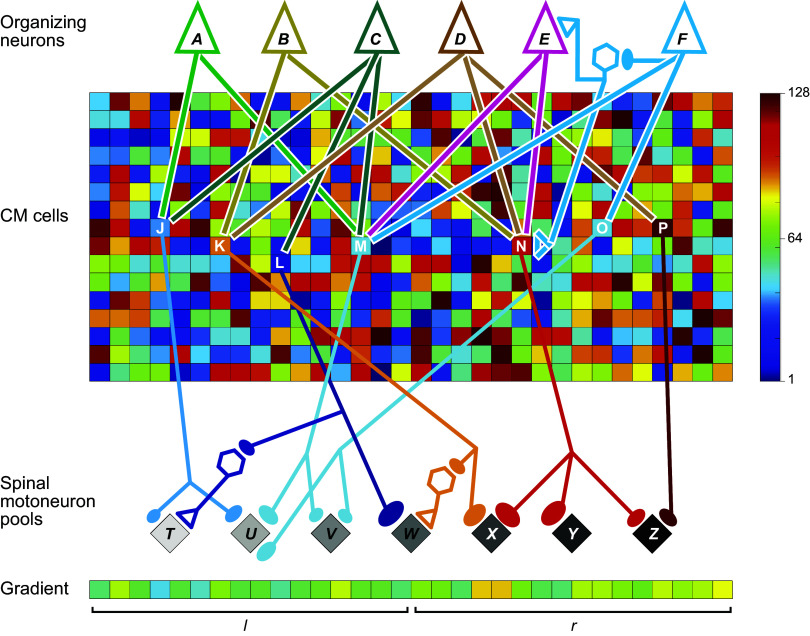
The cortical hyper-piano. Cortico-motoneuronal (CM) cells in the output layer of new M1 are depicted as an array of 512 colored cells. The 128 different colors represent 128 different patterns of divergence from single CM cells to spinal motoneuron pools, which are represented by the gray diamonds below. Most CM cells (e.g., *J*, *M*, *N*, *O*) have outputs that monosynaptically facilitate more than one muscle (filled ellipses), while others (e.g., *K*, *L*) also suppress particular muscles through segmental inhibitory interneurons (open hexagons and triangles), and still others facilitate only a single muscle (e.g., *P*). Hypothetical organizing neurons (ONs) facilitate various combinations of CM cells to produce different individuated movements. In addition to facilitation of CM cells, for some movements, the ONs may suppress unintended muscle activity through intracortical inhibitory interneurons (open hexagon and triangles). Although the intermixed distribution of the various CM cell types (colors) may appear random, column-wise averaging of the color values (1 to 128) reveals a subtle gradient (*bottom*) from left (*l*) with more pale blue cells, to right (*r*) with more yellow cells.

Classically, M1 was likened to a piano keyboard: a well-ordered, sequential representation of different muscles or elemental movements upon which the rest of the brain could play sophisticated melodies of movement. However, such a well-ordered, sequential representation of muscles already is present in the arrangement of motoneuron pools in the spinal cord (176). Considering this fact together with the features of the distributed organization of M1 and the complexities of CM-cell activity described above, we propose that to individuate movements from evolutionarily older synergies, the projection from M1 to spinal motoneuron pools acts instead as a “hyper-piano,” a distributed network of diverse CM-cell elements, illustrated schematically in FIGURE 10. Here, the M1 output “layer” of CM cells is depicted as a 512 element array of 128 different types of CM cell, each type represented by a different color. Although each of the 128 types is present ∼4 times, the different types are intermingled spatially, without overt segregation of single or closely related types. The lower row of gray diamonds represents the spinal motoneuron pools (SMPs) of seven different muscles. The top row of triangles represents hypothetical “organizing” neurons (ONs) that recruit CM cells in different combinations to individuate different movements. Each ON provides concurrent input to selected CM cells. Although the nature and location of these ONs are as yet unknown, CM cells in caudal, “new” M1 receive monosynaptic input from layer III neurons (145). However, our hypothetical organizing neurons might also represent a more extensive network of neurons in M1 that receive inputs from other cortical motor areas (177) and/or from the cerebellum and basal ganglia via the thalamus (178, 179).

Two distinct movements are generated by ONs *A* and *B*, which activate distinct sets of spatially intermingled CM cells (*J*, *M* vs. *K*, *N*) that nevertheless facilitate separate groups of spinal motoneuron pools (*T*, *U*, *V* vs. *X*, *Y*, *Z*). Variations on each of these movements are created by other ONs. Compared to *A*, for example, ON *C*, activates an additional CM cell (*L*), thereby facilitating an additional motoneuron pool (*W*), while suppressing another (*T*). Likewise, compared to ON *B*, ON *D* activates an additional CM cell (*P*), intensifying the facilitation of one of the three motoneuron pools (*Z*) otherwise facilitated by ON *B* via CM cell *N*. Whereas these four movements may be used frequently, uncommon movements can be generated as well. For example, ON *E* creates a substantially different movement by activating CM cells (*M*, *N*) that facilitate a larger, mixed set of motoneuron pools (*U*, *V*, *X*, *Y*, *Z*).

ON *F* illustrates two additional points. First, in addition to facilitating two CM cells (*M*, *O*), ON *F* facilitates an intracortical inhibitory neuron that suppresses both another ON (*E*) and a CM cell (*N*), thereby minimizing any contraction in unintended muscles (*X*, *Y*, *Z*). Second, the two CM cells facilitated by ON *F*, CM cells *M* and *O*, share target muscles (*U*, *V*). The common input from ON *F* will increase the frequency of synchronized spikes in these two CM cells that share two target muscles.

The distribution of different CM-cell types (represented by the 128 different colors) in our 512-element array might appear to be entirely random, but closer inspection reveals that dark blue hues are more common on the left whereas yellows, oranges, and dark reds are more common on the right. The row of cells labeled “Gradient” in FIGURE 10, *bottom*, shows the average of numbers represented by the colors of the cells in each column of the 512-element array. These averaged values all tend to be closer to the central color of the 1 to 128 range (green, numerically 64) than many of the individual 512 values, which vary from dark blue to dark red. However, the averaged values nevertheless reveal a subtle gradient. Those in the left half (*l*) tend to be somewhat smaller (more pale blue cells, numerically <64), whereas those in the right half (*r*) tend to be somewhat larger (more yellow cells, numerically >64). Although not obvious when looking at the overall 512-element array, an underlying gradient is present.

Threshold electrical stimulation on the left side of the array, therefore, might be more likely to activate one group of motoneuron pools (*T*, *U*, *V*), whereas threshold electrical stimulation on the right might be more likely to activate a different group (*X*, *Y*, *Z*). Bold fMRI activation during the movements created by ONs *A* or *C* would be slightly stronger on the left, but slightly stronger on the right during the movements created by ONs *B* or *D*. Small laterally situated lesions in human M1 would impair the thumb and index more than the middle, ring, and little fingers, or vice versa for small medially situated lesions, but could not paralyze just one digit (180–183). Although all 128 CM-cell types are found throughout the 512-element array at the microscopic scale, the distributed organization within the M1 hand representation on the microscopic scale thus is not incompatible with the degree of somatotopic gradient found on the meso- or macroscopic scale with electrical stimulation, fMRI, or cortical lesions (184, 185).

### 6.6. Advantages of Distributed Organization within the M1 Hand Representation

We suggest that the distributed organization of M1 is not simply a vestige of evolution but rather has been preserved because of its functional importance. The primary utility of distributed organization arises from the passive coupling between the digits and the active and reactive biomechanical interactions in the hand, discussed in sect. 4. Even if entirely independent muscles moved each digit, these two factors would require the nervous system to activate additional muscles stabilizing other digits and the wrist in performing an individuated movement of any given digit.

Thus normal use of the hand always involves controlling all the digits at the same time, even though control may differ among the digits. During a precision pinch to pick a raspberry, for example, the middle, ring, and little fingers must be held actively in a posture that prevents them from interfering with the action of the thumb and index finger needed to grasp the berry. Even during skilled performances in which single digits commonly are thought to strike different keys at different times, such as typing on a keyboard or playing a piano, multiple digits actually are in active motion at the same time. As one digit strikes a key, other digits are held away, while still others are moving into position for the following keystroke (186–188) (see sects. 7.3. and 7.4). Spatially segregating the cortical elements that facilitate either concurrent activity in different muscles or movements of different digits offers no advantage if any individuated movement requires simultaneous control of multiple digits, especially in view of the fact that a well-ordered central representation of different muscles exists in the spinal motoneuron pools. Rather, the distributed organization of M1, in which representations of various combinations of muscles or movement components are intermingled, may provide more efficient neural processing.

We note that the different parts of the face, as well as the different parts of the foot, each probably have passive coupling and mechanical interactions comparable to that within the hand. However, the between-group coupling of face, hand, and foot is substantially less. Spatial segregation of face, hand, and foot representations in M1 may be advantageous in view of their relative mechanical independence from one another (189).

A second advantage of distributed organization is the substrate it creates for rapid, flexible, recombination of motor outputs. In FIGURE 10, ON *E* illustrates that novel combinations of CM cells may create novel movements by recombining CM cells typically used for more common movements. Evidence of such rapid recombination comes from a study in which monkeys were rewarded for coactivating a CM cell with different muscles at different times (170). When a monkey was rewarded for coactivating a particular CM cell with the radial compartment of FDP (FDPr), for example, PSFs appeared in FDPr and extensor carpi radialis brevis (ECRB), but no effect occurred in palmaris longus (PL). However, when the monkey was rewarded for coactivating the same CM cell with PL, the PSF in FDPr remained unchanged, the PSF in ECRB diminished, and a new PSS appeared in PL. These observations indicate that the recorded CM cell could be activated with different sets of other last-order inputs to the spinal motoneuron pools of FDPr, ECRB, and PL, each set presumably including many other CM cells. These shifts from one set to another occurred within a few minutes.

**FIGURE 11.**
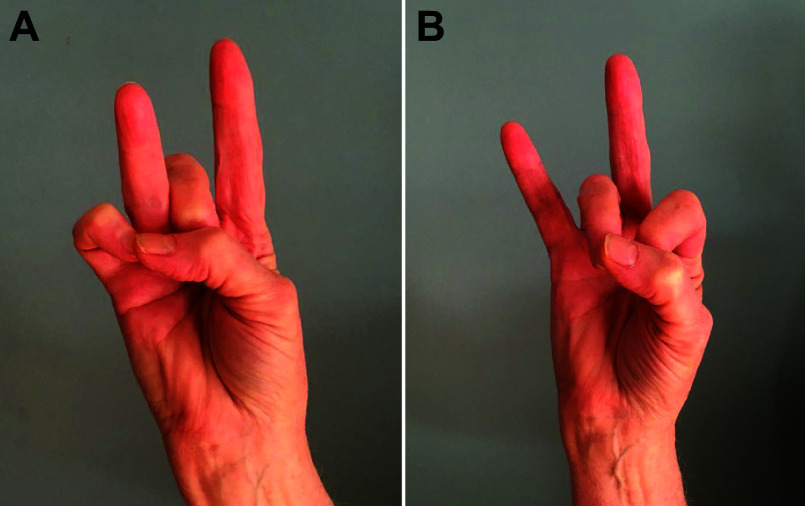
Novel hand postures. *A*: the index and ring fingers are extended, while the middle and little fingers are held flexed with the thumb. *B*: the middle and little fingers are extended, while the index and ring fingers are held flexed with the thumb.

We suggest that even more so than monkeys, humans can rapidly generate novel movements. For example, you may have never before put your hand in either of the postures shown in FIGURE 11. However, in less than a minute you probably can produce one posture and then the other (without using your other hand). With a bit of practice, you can alternate between them. We suggest that this ability to generate novel movements so rapidly reflects the ease with which new combinations of CM cells can be activated, a capability provided by the distributed, intermingled organization of M1. We speculate further that the extensive diversity of CM cells with outputs distributed to different sets of target muscles, together with the ability to recombine activation of various CM cells through organizing neurons, provides humans with an extraordinarily wide repertoire of potential individuated movements that can be produced rapidly.

## 7. INDIVIDUATION AND DEXTERITY

Individuation is only one facet of the enhanced human capability referred to as dexterity. To achieve dexterity, individuation must be combined with additional features, a complete list of which has yet to be delineated. We discuss four features that become integrated with individuation in the emergence of dexterity: the capability for precise control of position, timing, and force; the coordination of forces among the digits; the ability to withhold and release; and the ability to vary a given movement depending on the preceding or following movement.

### 7.1. Precision Control

Precise control of position, force, and timing combines with individuation in a number of dexterous skills. Pointing, for example, is a common action that requires precise control of position. As the arm moves the fingertip toward the target, an appropriate hand posture is produced with individuated extension of the index finger and concurrent flexion of the other digits. If the fingertip is to point accurately at the target, the entire chain of joints from the shoulder to the distal interphalangeal joint of the index finger must be controlled precisely (190, 191). At a smaller scale, the “micromovements” of the digits used in microsurgery or microelectronic assembly are characterized by precise control of low force (∼0.2–1.0 N), low speed, and high spatial precision (∼150–200 µm) simultaneously in multiple digits (192, 193). In tapping out Morse code, typing, or playing the piano, skilled individuals perform individuated finger movements with precise timing that can involve keystroke intervals as short as 52 ms (194).

As might be expected, skilled pianists have higher levels of finger individuation than nonmusicians (195, 196), especially with their middle and ring fingers which show levels of individuation similar to the thumb and index finger in nonmusicians. Indeed, the gray matter volume of the hand area in the precentral gyrus correlates with the level of keyboard skill (197). These differences indicate that individuation can be increased through extensive training (198, 199).

However, beyond individuation, skilled pianists show a higher precision of force production and timing than unskilled individuals. When playing, skilled pianists use lower impulse and force to achieve the same loudness (indicating higher energy efficiency), and at slow tempi their application of key-depressing force is more consistent than that of novices (200). In a finger-tapping task, pianists also showed lower variance in intertap intervals (195). Although individuation and keystroke timing accuracy in skilled pianists are relatively invariant across a wide range of tempi (201), more variability in timing (rhythmicity), but not force, occurs when less individuated digits play notes (202). Musical performance thus combines high levels of individuation with precise control of force and timing, not only in producing single notes but also in producing precisely controlled sequences of notes.

### 7.2. Coordination among the Digits

Performing any manual task requires controlling multiple digits at the same time. The required coordination among the digits brings out features not necessarily evident in single-finger tasks. Such coordination has been examined in terms of force sharing, multidigit grasping, and novel posture production.

When forces are produced simultaneously in multiple digits, the nervous system generates specialized patterns of coordination that may play important roles in dexterous grasping and manipulation. For example, when subjects are asked to press simultaneously with multiple fingers, generating a maximal total force across the fingers, the variance of the maximal total force output from trial to trial is smaller than the sum of variances of the maximal individual finger forces (35, 203). This indicates a pattern of coordination in which small decreases in the force exerted by some fingers unconsciously compensate for small increases in the force exerted by other fingers and vice versa. Note that such a “force-stabilizing synergy” constitutes a reduction of enslaving. As the total force is increased, however, the force-stabilizing synergy decreases, the forces exerted by individual fingers become more synchronized, and hence the variability of total force increases (204).

The force-stabilizing synergy also decreases in anticipation of a change in the total force, decreases further as the total force changes, and then returns as the total force stabilizes at a new level. These changes occur whether the subject exerts a brief pulse of total force or a gradual ramp or if one finger is suddenly unloaded (35, 205, 206). Interestingly, this decrease in the force-stabilizing synergy is less in the right hand than in the left hand of right-handed subjects, suggesting that neural control of the dominant hand may be specialized for dealing with dynamic force adjustments in multidigit tasks (207).

A drop in the force-stabilizing synergy means that the forces exerted by individual fingers tend to increase or decrease in parallel. In addition to changing the total force being exerted, such parallel changes in force across the digits tend to stabilize the moment of rotation around the proximodistal axis of the hand and forearm (203, 208, 209). Such moment stabilization is critical when multiple fingers hold an object against the thumb (210). Without moment stabilization the object would rotate and wobble around the thumb.

Other special patterns of coordination appear when humans oppose the thumb to other fingers. The human thumb is the most highly individuated of the five digits. The muscles, tendons, and ligaments of the thumb, and especially the structure of its carpo-metacarpal joint, have evolved such that the human thumb can be rotated out of the plane of the palm, bringing the tip of the thumb opposite that of any of the fingers (211–213). When humans oppose the thumb to the index fingertip in isotonic movements, the degree of flexion of the thumb varies from trial to trial, but the angles of the index finger MCP and proximal interphalangeal (PIP) joints are coordinated with those of the thumb such that the two digits meet consistently at the distal ends of their distal pads (214).

When the thumb opposes any one of the fingers under isometric conditions, another coordination pattern appears. If isometric flexion forces are produced with two digits simultaneously, less unintended force is produced by other digits when one of the two instructed digits is the thumb than if both instructed digits are fingers, and this phenomenon is more marked in the dominant right hand than in the left (38). Enslaving thus is reduced when the thumb acts in opposition to one of the fingers, particularly in the dominant hand, again suggesting that the dominant hand may be specialized for dealing with multidigit force coordination.

In human multidigit grasping the digits are shaped to suit the target object before contact (215, 216). Once contact occurs the force vectors produced by the digits must sum to zero if the object is not to move in either translation or rotation (217). When grasping a familiar object, then, the locations at which the digits will contact the object are selected before contact, and to minimize tilting, the forces exerted by each digit are adjusted rapidly through the sensorimotor cortex using online feedback to compensate for variability in contact locations (218–220). Particularly if the object is compliant (soft, e.g., an overripe tomato) rather than rigid (hard, e.g., a jar), voluntary changes in the direction of force exerted by the thumb are compensated by changes in the forces exerted by the opposing fingers, along with an increase in the grasp force exerted by each digit normal to the object’s surface (221).^3^

Once grasped, motion of the arm can translate the object through space. Such translation is accompanied by cocontraction of muscles acting on all the engaged digits, increasing the grasp forces and stiffening the hand (222). In contrast, within-hand rotation of a grasped object is achieved by reciprocal comodulation of muscles to produce the necessary rotational moment while also maintaining grasp, all as the center of pressure at each finger pad progressively shifts (223). The complexities of within-hand manipulations that require releasing one or more digits, exerting the dynamic forces needed to move the object within the hand, and then reestablishing a stable grasp, such as flipping your pencil to bring the eraser end to the paper, have yet to be investigated.

Humans show a remarkable ability to coordinate the digits in novel postures as well (e.g., FIGURE 11). With only a few days of practice, human subjects learned to control a 2-dimensional cursor through an arbitrary geometric mapping of 19 finger joint angles (224–226). Improvement in performance involved reduction of motion in the task-irrelevant dimensions (null-space) as well as reduction of variability in both task-relevant and task-irrelevant dimensions. Ultimately, the motion of the cursor was controlled smoothly by coordinating the movement of the digits through quite unusual postures. We suggest that learning these arbitrary mappings reflects rapid selection of new combinations of CM cells in M1 (e.g., FIGURE 10).

### 7.3. Withholding and Releasing

In using most tools and instruments, humans flex selected digits to hold the tool or to depress keys or levers, possibly reflecting the fact that individuation is higher and enslaving lower for finger flexion than for extension. As some fingers flex to grasp an object or play a note, others are held actively in a posture that avoids contact. Actively holding noninstructed digits in an extended posture can provide a certain range through which instructed digits can flex with little to no motion of noninstructed digits (227, 228). Such active withholding may involve both intracortical inhibition of flexor muscle activation and active contraction of extensors to check unintended flexion (see sects. 4.2 and 6.4).

Beyond active withholding of certain digits, dexterity also involves individuated extension to release selected digits from contact with an object. In playing the piano, for example, extending the finger to release a key determines the time at which the note ends. The manner of key release is critical for the quality of the tone. Interestingly, in playing a descending scale on the same string of a violin, the finger that determines the pitch of a note often flexes to depress the string tens of milliseconds before the onset of its note. The timing of onset then is determined by the release of the finger controlling the pitch of the preceding note (229).

Although digit release might seem to require only precise control of timing, precise control of position and force also can be critical in releasing. An example is found in the act of throwing a ball. In accurate overarm throwing by recreational athletes, the fingers are extended such that the ball is released within a 10-ms window around the moment the palm is vertical (230). Errors hitting a target in the vertical dimension occur if the fingers extend too early (ball released too high) or too late (ball released too low) relative to the angle at the wrist (231). Skilled throwers can release the ball with a timing precision as low as 1 ms (232). However, this release is not simply a matter of the timing of finger extension. Newtonian physics dictates that the forces exerted by the fingers acting to accelerate the ball oppose forces from the mass of the ball acting on the fingers. Although the finger joints are gradually extending in the 50 ms before release, the fingers continue to exert gradually decreasing flexion force with a brief increase as the ball rolls up off the fingertips, breaking contact (233). The fingers then actually flex briefly before resuming extension. The greater the mass of the ball, the greater the flexion force the fingertips must exert until contact is broken. Skilled throwers therefore most likely use a strategy that regulates the stiffness of the fingers rather than the force exerted, automatically compensating for variation in the mass or acceleration of the ball (232). Many within-hand manipulations, such as rotating a pencil to bring the eraser end down to the paper, likewise require releasing the pressure of certain digits as the force exerted by others changes direction and increases.

### 7.4. Coarticulation

The term “coarticulation,” as originally applied in studies of speech sounds to describe kinematic variations in the production of one phoneme depending on the preceding and following phonemes (234), has been used to describe a similar aspect of dexterous finger movements. We often think of sequential individuated finger movements as occurring one at a time. However, kinematic examination has shown that before pianists strike a particular note using one digit, the motion of that digit and of the other digits, as well as the underlying finger muscle EMG activity, already may differ depending on how the preceding and subsequent notes are played (187, 188, 235). These differences are particularly dramatic, for example, when the right thumb must swing rightward versus leftward under the palm to play a note higher versus lower than the preceding note. Coarticulation also occurs in fluent finger spelling of American Sign Language, where thumb and wrist angles tend to minimize differences between sequential hand shapes while index and middle finger PIP angles emphasize the differences (236). Coarticulation thus is an important aspect of fluent performance.

## 8. IMPAIRED INDIVIDUATION AND RECOVERY

Lesions of many different parts of the nervous system can impair individuated finger movements. Most such impairments, however, result from damage to the primary motor cortex and/or the corticospinal tract, or damage to structures such as the cerebellum or basal ganglia that affect individuated movements through transthalamic projections to the motor cortex. We therefore focus on lesions of M1 or the CST, in which impaired individuation is part of the syndrome of hemiparesis.

**FIGURE 12.**
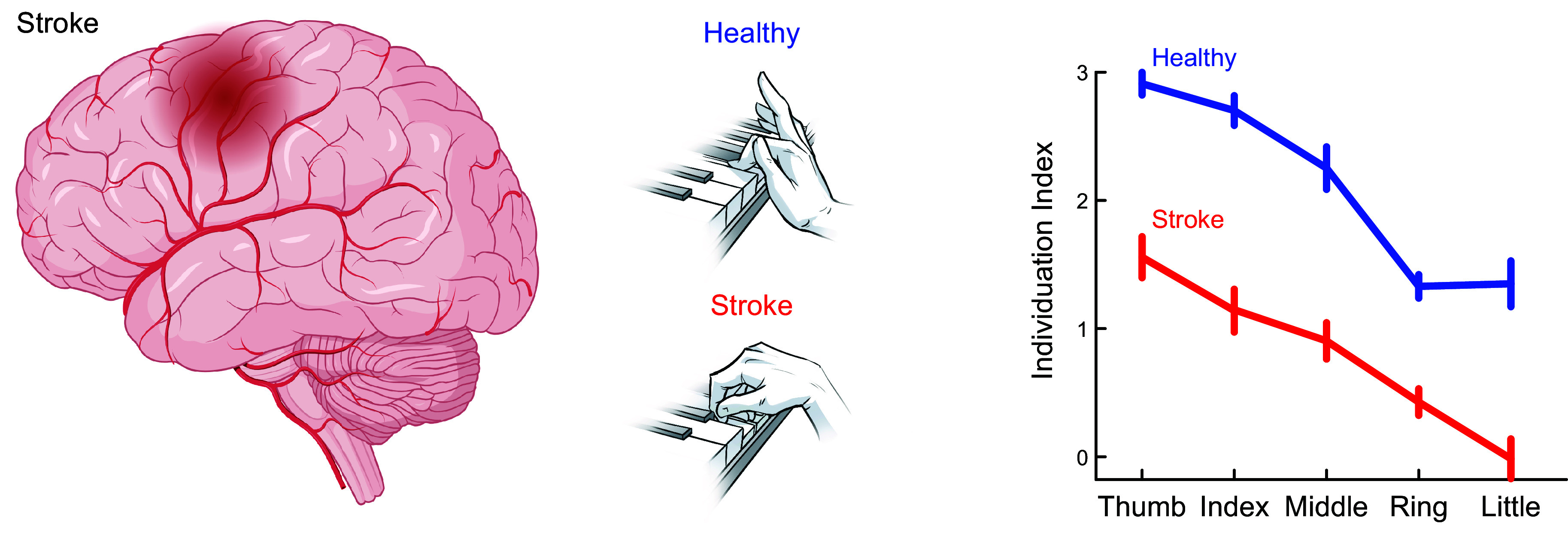
Individuation of finger movements is decreased after stroke involving the hand representation of the motor cortex or corticospinal tract. Data at *right* were reproduced from Ref. 237, with permission from the authors.

Neurologists have long known that when hemiparesis in humans progresses gradually (as may occur with an infiltrating tumor), it is individuated movements of the fingers that are affected earliest (Ref. 1, p. 342–344), indicating that individuation is the function most sensitive to damage of M1 or the CST. Before the patient’s hand and arm become overtly weak or spastic, the difficulty appears in tasks such as buttoning buttons and tying shoelaces. The normal variety of human hand and finger movements becomes more stereotyped. Conversely, when the human M1 or CST is damaged suddenly by stroke (FIGURE 12), diffuse weakness of the arm and hand is prominent initially, any recovery is earlier and more complete proximally than distally, and individuated finger movements typically are the last to recover and most likely to remain permanently impaired (238).

Similar to stroke in humans, experimental ablation of M1 or a section of the CST at the medullary pyramid in monkeys initially produces severe weakness of the contralateral limbs (239, 240). Although ambulation as well as the grasping movements used in climbing recover substantially (further and faster than in humans), other movements of the upper extremity remain comparatively stereotyped. Precision pinch movements may not recover at all (241). Deficits in movement speed and force production may persist (242).

Recovery after experimental lesions provides time for plastic reorganization of the remaining neural systems. In contrast, intracortical injection of the GABA_A_ agonist, muscimol, rapidly but reversibly inhibits cortical activity, providing little to no opportunity for reorganization. When muscimol is injected into the monkey M1 hand representation, movements of the contralateral digits become impaired. Individuation is reduced, along with the strength and speed of finger movements (243–245). Such deficits emphasize the importance of M1 for generating normal individuated finger movements.

After stroke in humans, most recovery of both finger individuation and finger strength occurs in the first few months. Three recent findings provide evidence, however, that different processes underlie recovery of individuation versus strength (246). First, the extent of damage to the M1 hand area and/or the CST correlated more strongly with poorer recovery of individuation than of strength. Second, whereas strength shows substantial recovery in the first month after stroke, individuation shows more recovery in the second and third months. Third, although recovery of strength up to ∼60% of its estimated premorbid baseline was correlated with recovery of individuation, additional recovery of strength was not correlated with additional recovery of individuation. Although substantial recovery may thus occur, compared to healthy controls or to the nonparetic hand, finger individuation in the recovered hand often remains reduced in both flexion/extension and abduction/adduction, and enslaving remains increased (27, 247, 248).

Greater weakness and loss of individuation in finger extension than in flexion (Refs. 39, 237, 249, 250; see, however, Ref. 251) combined with the development of a bias to activate the flexors together in a pathological synergy, constitutes a particular and persistent problem after stroke in humans (238, 252–254). Experimental studies in nonhuman primates have provided insight into the mechanisms underlying these changes (255). First, because CM cells in healthy M1 on average facilitate finger extensors more strongly than flexors (65, 69, 256), the loss of CM cells will result in relatively greater impairment of extension than flexion. Second, reorganization of other descending pathways results in increased facilitation of flexors. Following a CST lesion, rubrospinal outputs lose their normal preferential facilitation of wrist and digit extensor as compared to flexor muscles (257). Likewise, stimulation of reticulospinal axons in the median longitudinal fasciculus produces larger than normal EPSPs in flexor and intrinsic hand muscle motoneurons, but not in extensor motoneurons, with the total synaptic input to intrinsic hand motoneurons increasing 2.5-fold (258). We suggest that these changes combine to produce a bias toward flexor activation by subcortical neural structures, which together with the decreased variability in the direction of three-dimensional force production among noninstructed digits reflecting loss of cortical control, results in the decreased individuation observed in chronic human hemiparesis (39, 239, 259–261).^4^

In monkeys with chronic lesions of M1, practicing a task that requires use of the impaired hand to grasp food can lead to recovery of finger movements, in macaques including an altered precision grip (262, 263). Studies in which the spinal dorsolateral funiculus has been sectioned at the C4/C5 border, eliminating mono- and disynaptic connections to hand motoneurons from M1 (and from the red nucleus) while leaving intact connections to the C3-C4 propriospinal neurons and from these propriospinal neurons to spinal motoneurons, suggest that the C3-C4 propriospinal neurons may play a role in recovery of some ability to retrieve food morsels with a grasp between the index finger and thumb (104, 105, 264, 265). However, the success rate and kinematics of the grip recovered following a C4/5 lesion, as well as the underlying muscular and cortical activity, were not that of normal macaques. Compared to normal, the success rate of true precision pinch between the tip of the index finger and thumb was reduced, and the recovered grip used to retrieve a food morsel from a narrow slot often was not such a precision pinch (105). The recovered grip was produced with more extensive cocontraction of finger and wrist muscles than normal, and the EMG activity of multiple muscles included coherent 30- to 46-Hz oscillations that did not involve the motor cortex (266). The recovered grip also was accompanied by extensive activation of the primary and premotor cortex bilaterally (267).

In humans, practicing a task that requires individuated finger movements even after 6 months or more of recovery can lead not only to improved performance of the trained task but also to improved functional use of the hand (268–271). Such studies have yet to compare the detailed kinematics of task performance and underlying EMG activity of the trained paretic hand with that of the nonparetic hand or normal controls. Although systematic restoration to prestroke levels of functional individuation is not yet generally achievable, various studies employing different combinations of finger motion transducers, visual feedback, virtual reality environments, and exoskeleton support, indicate that focusing on movement quality (i.e., overcoming the flexion bias to individuate finger movements without invoking compensatory movement of other body parts) and high-intensity practice are two important factors in achieving improvement.

## 9. CHALLENGES FOR THE DEVELOPMENT OF BIONIC HANDS

When lesions of the nervous system are so severe that recovery is not possible, or when amputation of the hand has occurred, current efforts to translate our knowledge of finger movements to control a bionic hand through a brain-machine interface (BMI) offer hope for restoration of function (272). However, the development of bionic hands with the full dexterity of the native human hand faces many challenges (272–274). Current efforts by and large focus on the near-term development of a utilitarian hand capable of grasping a reasonable variety of objects, without attempting to emulate the musculoskeletal structure of the native hand or its natural neural control. In the long-term, however, as efforts extend to develop bionic hands capable of the full spectrum of individuated finger movements, epitomized for example by within-hand manipulations, emulation of the native hand’s neuromuscular structure and control may prove valuable. While a number of challenges concern a wide range of neuroprosthetic devices, here we comment on five particular aspects that might be informed by our current knowledge of individuated finger movements.

First, most current bionic hands make little attempt to emulate the biomechanical structure of the native muscles and tendons that move the digits (275, 276), particularly the intrinsic muscles of the hand, which not only abduct and adduct the digits but also control the relative attitude of the proximal and distal interphalangeal joints (277, 278). The few bionic hands that do so suggest that such emulation may provide certain advantages, including more naturalistic patterns of finger stiffness versus compliance that allow the fingers to adapt to the shape of various objects while applying forces adequate for stable handling (279, 280). The approach of functional electrical stimulation (FES) in particular aims to restore movements to paralyzed users by stimulating the intact native nerves and muscles (281–283). While simple grasping by opening and closing the digits has been produced, achieving truly dexterous individuated movements through FES will require the implementation of a more detailed understanding of how the nervous system normally activates multiple muscles for any individuated finger movement.

Second, as we have emphasized, native individuated finger movements entail active motion of, and/or force production by multiple digits, while other digits are stabilized or withdrawn, typically requiring active control of many, if not all, of the 23 mechanical degrees of freedom (DOFs; axes of joint rotation) in the digits and wrist. Current efforts to make functional use of a multifingered bionic hand utilize only a few fixed combinations of these 23 DOFs (284–287). As near-term development proceeds, a greater variety of grasp shapes may be achieved by controlling increasing numbers of “kinematic synergies,” each comprising the simultaneous motion of multiple DOFs in the fixed proportions most common in daily use of the hand (20, 288, 289). While controlling several more kinematic synergies might provide a more useful bionic hand, achieving truly dexterous individuated finger movements is likely to require many more.

Third, a limiting factor in controlling the numerous DOFs needed for dexterous individuated finger movements may be the number and nature of the input signals available for a BMI. Currently, no more than 100–200 channels recorded from high-density EMG, peripheral nerves, electrocorticographic grids, or cortical microelectrode arrays are being utilized, yet thousands of channels may be needed. This will require the development of neural interfaces with an order of magnitude more input channels. Recordings directly from the muscles of the forearm and hand or the nerves that innervate them in theory can provide detailed signals similar to those that control the native hand (290–292). However, current recordings from the cerebral cortex collect signals (whether electrocorticographic or spiking activity) from the surface of the precentral gyrus or posterior parietal cortex, which are less directly related to the signals that naturally drive finger muscles. While the intention to move each of the digits individually can be decoded from these signals (286, 293–295), for a BMI to control dexterous individuated finger movements from thousands of such input channels may require training machine learning/deep neural network/artificial intelligence algorithms (291, 292). Furthermore, in the long-term, neural interfaces capable of recording from human area 4 in the depth of the central sulcus may provide signals optimal for control of dexterous individuated finger movements.

Fourth, the utility of finger movements in handling objects is limited without both tactile and proprioceptive feedback informing the CNS of the location of contact points on the digits and palm, the magnitude and direction of forces being exerted at each point, and their spatial relationship to one another. In the near-term, electrical microstimulation is being developed both in peripheral nerves (290, 296) and in the somatosensory cortex (297, 298) to deliver feedback from sensors embedded in bionic hands. Although the temporal modulation of electrical pulse trains can be made to mimic that of the spike trains evoked by natural stimulation (299), the very nature of electrical stimulation is unnatural, in that numerous neurons are stimulated simultaneously by each pulse, with larger axons or somata being excited more readily than smaller ones (300). In the long-term, methods that can stimulate neurons more selectively may enable the delivery of more detailed feedback. Furthermore, intracortical microstimulation of the human somatosensory cortex currently is limited to Brodmann’s areas 1 on the surface of the precentral gyrus. The ability to deliver feedback in proprioceptive area 3a and tactile area 3b in the depth of the central sulcus may enable more detailed somatosensory feedback on the instantaneous position and contacts of the fingers.

Fifth, we have reviewed the contribution of a number of subcortical centers that normally participate in individuated finger movements, providing multiple disynaptic pathways from the cortex to motoneuron pools. Although their locations deep in the brain and spinal cord render them less accessible than the cerebral cortex, peripheral nerves, or muscles, in the long-term, the ability to add multichannel signals from subcortical centers as part of the input to BMIs may further enhance not only the production of dexterous individuated finger movements but also the necessary sensory feedback.

## 10. WHAT DOES INDIVIDUATION GIVE HUMANS?

Evolution of the ability to individuate finger movements arguably played a major role in the prehistoric evolutionary success of the human species. Monkeys and apes can hold a stone or a stick with a power grasp. However, producing a sharp stone point and lashing it to the shaft of an arrow required more highly individuated finger movements, as has the subsequent development of the large majority of human tools and instruments to the present day. Making skilled use of such tools and instruments likely depended not only on the evolution of monosynaptic connections from new M1 to spinal motoneurons (301) but also on the elaboration of additional cortical motor areas that both communicate with M1 and provide disynaptic inputs to motoneurons via spinal interneurons (302, 303).

While perhaps most apparent in the human hand, we suggest that individuation underlies many of the more sophisticated movements we make with all parts of our body. The movements of the arms, legs, and trunk used in dancing (ballet, flamenco, etc.) have been individuated from those used in reaching and walking. The movements of the mouth, tongue, pharynx, larynx, and diaphragm used in speaking have been individuated from those used for eating and breathing (302, 304, 305). The capability for individuated movement thus provides the infrastructure for the skills humans acquire to express cognitive activity, from manipulating sophisticated tools, to handwriting and drawing, to typing and playing musical instruments, to dancing, to speaking. By vastly expanding the repertoire of nuanced movements available, the evolution of individuation enabled the external expression of coevolving human cognition, leading to the exponential expansion of invention and creativity that characterizes human history.

## 11. OPEN QUESTIONS AND FUTURE DIRECTIONS

Although much has been learned about individuated movement in the past several decades, much has yet to be understood. As technological capabilities continue to expand, in addition to meeting the challenges for the development of a truly dexterous bionic hand (see sect. 9), we look for a number of outstanding issues to be investigated.

Behaviorally, how individuation interacts with other motor control variables (described in sect. 7) to give rise to human dexterity remains to be elucidated. In particular, further studies will be needed to better understand the dynamic control of multiple fingers simultaneously in complex sequential tasks such as within-hand manipulation, typing, and musical performance.

As described in sects. 5 and 6, many single neurons in the CNS centers that provide descending voluntary control of movement send last-order inputs to multiple motoneuron pools (FIGURE 8). However, the extent to which such neurons are activated primarily when all their target muscles are recruited (creating a synergy), or instead are activated as a network that flexibly recruits the necessary muscles at the right time (FIGURE 10), remains a matter of debate. Studies examining the simultaneous activity of many such neurons during substantially wider ranges of movements are needed to address this issue. Moreover, the various centers providing these last-order inputs, segmental, propriospinal, reticulospinal, rubrospinal, and corticospinal, to date have been investigated separately. Studies that examine the activity of neurons in two or more of these centers simultaneously will be needed to understand how their activity combines to sculpt individuated movements. In addition, how our hypothetical “organizing” neurons (sect. 6.5) selectively activate the right combination of descending neurons for the intended movement remains an open question.

The roles of other CNS regions that provide inputs to the descending systems also warrant further investigation. We do not yet understand how inputs from the primary somatosensory cortex influence M1 to enable dexterous multidigit manipulation or how inputs from the premotor and supplementary motor cortex lead to the selection of the desired individuated finger movements at the right time, especially in rapid sequences such as typing. Clinical evidence indicates that subcortical inputs to M1 may also play important roles in individuated movements. Task-specific dystonias suggest contributions from the basal ganglia (306), and increased enslaving seen in cerebellar patients suggests that cerebellar inputs play an important role as well (307).

Finally, does individuation play a role in human concept formation? Could humans have imagined making and playing a flute, for example, if they had lacked the individuated finger movements to do so? The role of individuation in human cognition and creativity remains uncharted territory in evolutionary biology, neuroscience, and cognitive science.

## GLOSSARY

Joints

DIP: Distal interphalangeal joint
MCP: Metacarpophalangeal joint
PIP: Proximal interphanalgeal joint

Digits:

Digit 1: Thumb
Digit 2: Index finger
Digit 3: Middle finger
Digit 4: Ring finger
Digit 5: Little finger

Muscles

ADM: Abductor digiti minimi, abducts the little finger, intrinsic
ADP: Adductor pollicis, adducts the thumb, intrinsic
APB: Abductor pollicis brevis, abducts the thumb, intrinsic
APL: Abductor pollicis longus, abducts the thumb, extrinsic
FDI: First dorsal interosseous, abducts the index finger, intrinsic
ECRB: Extensor carpi radialis brevis, extends the wrist
ECU: Extensor carpi ulnaris, extends the wrist
EDC: Extensor digitorum communis, extends the four fingers, extrinsic
EDM: Extensor digiti minimi, extends the little finger, extrinsic
EIP: Extensor indicis proprius, extends the index finger, extrinsic
ED23: Extensor digiti secundi et tertii (macaque), extends the index and middle fingers, extrinsic
ED45: Extensor digiti quarti et quinti (macaque), extends the ring and little fingers, extrinsic
EPB: Extensor pollicis brevis, extends the thumb, extrinsic
EPL: Extensor pollicis longus, extends the thumb, extrinsic
FCU: Flexor carpi ulnaris, flexes the wrist
FDS: Flexor digitorum superficialis, flexes the four fingers at their PIP joints, extrinsic
FDP: Flexor digitorum profundus, flexes the four fingers at their DIP joints, extrinsic
FDPr: Flexor digitorum profundus, radial compartment, flexes the more radial digits in macaques
FDPu: Flexor digitorum profundus, ulnar compartment, flexes the more ulnar digits in macaques
OP: Opponens pollicis, opposes the thumb to other digits, intrinsic
PL: Palmaris longus, flexes the wrist
SDI: Second dorsal interosseous, abducts the middle finger radially, intrinsic

Other

BMI: Brain-machine interface
CM cell: Cortico-motoneuronal neuron, a cortical neuron that makes monosynaptic connections to spinal motoneurons
CNS: Central nervous system
CPG: Central pattern generator
CST: Corticospinal tract
DOFs: Degrees of freedom
EMG: Electromyographic
FES: Functional electrical stimulation
fMRI: Functional magnetic resonance imaging
M1: Primary motor cortex
MEP: Motor-evoked potential
MN: motoneuron, spinal
ON: Organizing neuron, hypothetical
PMRF: Pontomedullary reticular formation
PSE: Postspike effect
PSF: Postspike facilitation
PSS: Postspike suppression
ReST: Reticulospinal tract
RuST: Rubrospinal tract
sIN: Segmental interneuron
SMU: Single motor unit
STS: Short-term synchronization
TMS: Transcranial magnetic stimulation

## DATA AVAILABILITY

Data will be made available upon reasonable request.

## GRANTS

This work was supported by National Institute of Health (NIH) Grant R01NS130210 (to J.X.), R01-NS27686 (to M.H.S.), and R01-NS102343 (to M.H.S.); NIH CTSA UL1 award 2UL1TR002378 (to J.X.); Israel Science Foundation 2484/23 (to F.M.); and United States-Israel Binational Science Foundation Grant 2021323 (to F.M.).

## AUTHOR CONTRIBUTIONS

J.X., F.M., and M.H.S. conceived and designed research; J.X., F.M., and M.H.S. interpreted results of experiments; F.M. and M.H.S. prepared figures; J.X., F.M., and M.H.S. drafted manuscript; J.X., F.M., and M.H.S. edited and revised manuscript; J.X., F.M., and M.H.S. approved final version of manuscript.

BOX 1. NEUROMUSCULAR COMPARTMENTS

The motor units of any given muscle defined by gross anatomy generally are assumed to be activated by the CNS as a single pool and recruited and derecruited according to Henneman’s size principle (40). Many muscles, however, are comprised of 2 or more “neuromuscular compartments,” which the CNS can activate to some extent separately (41). Neuromuscular compartments generally are collections of motor units confined to a particular subvolume within the muscle belly and innervated by a separate branch of the muscle’s motor nerve. A given spinal motoneuron most commonly innervates muscle fibers within a single neuromuscular compartment of a single muscle. The different origins and/or insertions of a muscle’s neuromuscular compartments provide different mechanical actions. In particular, the separate neuromuscular compartments of the extrinsic multitendoned finger muscles (in humans, FDS, FDP, and EDC) enable the CNS to act on different fingers relatively selectively. (Note that the neuromuscular compartments within a single muscle discussed here are distinct from “muscle compartments” consisting of a few neighboring, anatomically distinct muscles and their neurovascular supply, all bundled together by fascia.)

## Footnotes

1 Note that because sampling EMG simultaneously from all the muscles serving the hand and arm has not been technically possible, the average number of muscles found to receive input from a single CM cell may be underestimated. Conversely, the fraction of M1 neurons that make monosynaptic connections to muscles may be underestimated as well. The same will apply to any of the sources of last-order inputs to motoneurons discussed in sect. 5.

2 The same will be true for last-order inputs to motoneurons from any source. Indeed, the vertebrate nervous system has only a few sites where EPSPs produced by a presynaptic neuron consistently drive action potentials in the postsynaptic element. These include the neuromuscular junction (end plate) and the climbing fiber input to cerebellar Purkinje cells.

3 Interestingly, this increase in grasp force exceeds that required for stable grasp, suggesting the action of a evolutionarily older neural system for maintaining grasp on which a more recently evolved voluntary manipulation is superimposed.

4 Interestingly, following lesions of the CST, monkeys do not develop the pathological flexion synergy/bias seen so commonly in humans. Rather, in monkeys the hand and arm are somewhat hypotonic (239, 259–261). This species difference suggests that in chronic hemiparesis the increase in reticulospinal (or other subcortical) drive to flexor motoneurons may be even stronger in humans than in monkeys.

## References

[1] Walshe FM. On the role of the pyramidal system in willed movements. Brain 70: 329–354, 1947. doi:10.1093/brain/70.3.329. 20273036

[2] Lemon RN. Descending pathways in motor control. Annu Rev Neurosci 31: 195–218, 2008. doi:10.1146/annurev.neuro.31.060407.125547. 18558853

[3] Grillner S. The motor infrastructure: from ion channels to neuronal networks. Nat Rev Neurosci 4: 573–586, 2003. doi:10.1038/nrn1137. 12838332

[4] Stein PS. Central pattern generators in the turtle spinal cord: selection among the forms of motor behaviors. J Neurophysiol 119: 422–440, 2018. doi:10.1152/jn.00602.2017. 29070633 PMC5867383

[5] Alstermark B, Isa T. Circuits for skilled reaching and grasping. Annu Rev Neurosci 35: 559–578, 2012. doi:10.1146/annurev-neuro-062111-150527. 22524789

[6] Georgopoulos AP, Grillner S. Visuomotor coordination in reaching and locomotion. Science 245: 1209–1210, 1989. doi:10.1126/science.2675307. 2675307

[7] Iwaniuk AN, Whishaw IQ. On the origin of skilled forelimb movements. Trends Neurosci 23: 372–376, 2000. doi:10.1016/s0166-2236(00)01618-0. 10906801

[8] Kiehn O. Decoding the organization of spinal circuits that control locomotion. Nat Rev Neurosci 17: 224–238, 2016. doi:10.1038/nrn.2016.9. 26935168 PMC4844028

[9] Whishaw IQ, Coles BL. Varieties of paw and digit movement during spontaneous food handling in rats: postures, bimanual coordination, preferences, and the effect of forelimb cortex lesions. Behav Brain Res 77: 135–148, 1996. doi:10.1016/0166-4328(95)00209-x. 8762164

[10] Whishaw IQ, Gorny B. Arpeggio and fractionated digit movements used in prehension by rats. Behav Brain Res 60: 15–24, 1994. doi:10.1016/0166-4328(94)90058-2. 8185848

[11] Whishaw IQ, Pellis SM. The structure of skilled forelimb reaching in the rat: a proximally driven movement with a single distal rotatory component. Behav Brain Res 41: 49–59, 1990. doi:10.1016/0166-4328(90)90053-h. 2073355

[12] Gorska T, Sybirska E. Effects of pyramidal lesions on forelimb movements in the cat. Acta Neurobiol Exp (Wars) 40: 843–859, 1980. 7234515

[13] Milliken GW, Ward JP, Erickson CJ. Independent digit control in foraging by the aye-aye (Daubentonia madagascariensis). Folia Primatol (Basel) 56: 219–224, 1991. doi:10.1159/000156551. 1937286

[14] Costello MB, Fragaszy DM. Prehension in Cebus and Saimiri: I. Grip type and hand preference. Am J Primatol 15: 235–245, 1988. doi:10.1002/ajp.1350150306. 31968893

[15] Fox DM, Mundinano IC, Bourne JA. Prehensile kinematics of the marmoset monkey: Implications for the evolution of visually-guided behaviors. J Comp Neurol 527: 1495–1507, 2019. doi:10.1002/cne.24639. 30680739

[16] Fragaszy DM. Preliminary quantitative studies of prehension in squirrel monkeys (Saimiri sciureus). Brain Behav Evol 23: 81–92, 1983. doi:10.1159/000121499. 6667371

[17] Garcia-Pelegrin E, Miller R, Wilkins C, Clayton NS. Manual action expectation and biomechanical ability in three species of New World monkey. Curr Biol 33: 1803–1808, 2023. doi:10.1016/j.cub.2023.03.023. 37019106

[18] Tanaka I. Social diffusion of modified louse egg-handling techniques during grooming in free-ranging Japanese macaques. Anim Behav 56: 1229–1236, 1998. doi:10.1006/anbe.1998.0891. 9819340

[19] Fragaszy D, Crast J. Functions of the hand in primates. In: *Developments in Primatology: Progress and Prospects. The Evolution of the Primate Hand*, edited by Kivell T, Lemelin P, Richmond B, Schmitt D. New York, NY: Springer, 2016, p. 313–344.

[20] Yan Y, Sobinov AR, Bensmaia SJ. Prehension kinematics in humans and macaques. J Neurophysiol 127: 1669–1678, 2022. doi:10.1152/jn.00522.2021. 35642848 PMC9208440

[21] Macfarlane NB, Graziano MS. Diversity of grip in Macaca mulatta. Exp Brain Res 197: 255–268, 2009. doi:10.1007/s00221-009-1909-z. 19565227

[22] Schieber MH. Individuated finger movements of rhesus monkeys: a means of quantifying the independence of the digits. J Neurophysiol 65: 1381–1391, 1991. doi:10.1152/jn.1991.65.6.1381. 1875247

[23] Hager-Ross CK, Schieber MH. Quantifying the independence of human finger movements: comparisons of digits, hands and movement frequencies. J Neurosci 20: 8542–8550, 2000. doi:10.1523/JNEUROSCI.20-22-08542.2000. 11069962 PMC6773164

[24] Kamara G, Rajchert O, Solomonow-Avnon D, Mawase F. Generalization indicates asymmetric and interactive control networks for multi-finger dexterous movements. Cell Rep 42: 112214, 2023. doi:10.1016/j.celrep.2023.112214. 36924500

[25] Lang CE, Schieber MH. Human finger independence: limitations due to passive mechanical coupling versus active neuromuscular control. J Neurophysiol 92: 2802–2810, 2004. doi:10.1152/jn.00480.2004. 15212429

[26] Johansson AM, Grip H, Ronnqvist L, Selling J, Boraxbekk CJ, Strong A, Hager CK. Influence of visual feedback, hand dominance and sex on individuated finger movements. Exp Brain Res 239: 1911–1928, 2021. doi:10.1007/s00221-021-06100-0. 33871660 PMC8277644

[27] Lang CE, Schieber MH. Reduced muscle selectivity during individuated finger movements in humans after damage to the motor cortex or corticospinal tract. J Neurophysiol 91: 1722–1733, 2004. doi:10.1152/jn.00805.2003. 14668295

[28] Venkadesan M, Valero-Cuevas FJ. Neural control of motion-to-force transitions with the fingertip. J Neurosci 28: 1366–1373, 2008. doi:10.1523/JNEUROSCI.4993-07.2008. 18256256 PMC2840633

[29] Abolins V, Latash ML. The nature of finger enslaving: new results and their implications. Motor Control 25: 680–703, 2021. doi:10.1123/mc.2021-0044. 34530403

[30] Zatsiorsky VM, Li ZM, Latash ML. Coordinated force production in multi-finger tasks: finger interaction and neural network modeling. Biol Cybern 79: 139–150, 1998. doi:10.1007/s004220050466. 9791934

[31] Zatsiorsky VM, Li ZM, Latash ML. Enslaving effects in multi-finger force production. Exp Brain Res 131: 187–195, 2000. doi:10.1007/s002219900261. 10766271

[32] Sanei K, Keir PJ. Independence and control of the fingers depend on direction and contraction mode. Hum Mov Sci 32: 457–471, 2013. doi:10.1016/j.humov.2013.01.004. 23643494

[33] Slobounov S, Johnston J, Chiang H, Ray W. The role of sub-maximal force production in the enslaving phenomenon. Brain Res 954: 212–219, 2002. doi:10.1016/s0006-8993(02)03288-2. 12414104

[34] Yu WS, van Duinen H, Gandevia SC. Limits to the control of the human thumb and fingers in flexion and extension. J Neurophysiol 103: 278–289, 2010. doi:10.1152/jn.00797.2009. 19889847

[35] Cuadra C, Bartsch A, Tiemann P, Reschechtko S, Latash ML. Multi-finger synergies and the muscular apparatus of the hand. Exp Brain Res 236: 1383–1393, 2018. doi:10.1007/s00221-018-5231-5. 29532100 PMC5936471

[36] Li ZM, Zatsiorsky VM, Latash ML. Contribution of the extrinsic and intrinsic hand muscles to the moments in finger joints. Clin Biomech (Bristol, Avon) 15: 203–211, 2000. doi:10.1016/s0268-0033(99)00058-3. 10656982

[37] Reilly KT, Hammond GR. Intrinsic hand muscles and digit independence on the preferred and non-preferred hands of humans. Exp Brain Res 173: 564–571, 2006. doi:10.1007/s00221-006-0397-7. 16505998

[38] Reilly KT, Hammond GR. Human handedness: is there a difference in the independence of the digits on the preferred and non-preferred hands? Exp Brain Res 156: 255–262, 2004. doi:10.1007/s00221-003-1783-z. 14712333

[39] Xu J, Ma T, Kumar S, Olds K, Brown J, Carducci J, Forrence A, Krakauer J. Loss of finger control complexity and intrusion of flexor biases are dissociable in finger individuation impairment after stroke. eLife 12: RP91495, 2023. doi:10.7554/eLife.91495.1.

[40] Gordon T, Thomas CK, Munson JB, Stein RB. The resilience of the size principle in the organization of motor unit properties in normal and reinnervated adult skeletal muscles. Can J Physiol Pharmacol 82: 645–661, 2004. doi:10.1139/y04-081. 15523522

[41] English AW, Wolf SL, Segal RL. Compartmentalization of muscles and their motor nuclei: the partitioning hypothesis. Phys Ther 73: 857–867, 1993. doi:10.1093/ptj/73.12.857. 8248294

[42] von Schroeder HP, Botte MJ. The functional significance of the long extensors and juncturae tendinum in finger extension. J Hand Surg Am 18: 641–647, 1993.doi:10.1016/0363-5023(93)90309-Q. 8349973

[43] Schuenke M, Schulte E, Schumacher U. Atlas of Anatomy. New York: Thieme Medical Publishers, Inc., 2016.

[44] Keen DA, Fuglevand AJ. Role of intertendinous connections in distribution of force in the human extensor digitorum muscle. Muscle Nerve 28: 614–622, 2003. doi:10.1002/mus.10481. 14571465

[45] von Schroeder HP, Botte MJ, Gellman H. Anatomy of the juncturae tendinum of the hand. J Hand Surg Am 15: 595–602, 1990.doi:10.1016/s0363-5023(09)90021-1. 2380523

[46] Leijnse JN, Walbeehm ET, Sonneveld GJ, Hovius SE, Kauer JM. Connections between the tendons of the musculus flexor digitorum profundus involving the synovial sheaths in the carpal tunnel. Acta Anat (Basel) 160: 112–122, 1997. doi:10.1159/000148003. 9673709

[47] Malerich MM, Baird RA, McMaster W, Erickson JM. Permissible limits of flexor digitorum profundus tendon advancement–an anatomic study. J Hand Surg Am 12: 30–33, 1987.doi:10.1016/s0363-5023(87)80156-9. 3805640

[48] Leijnse JN. Measuring force transfers in the deep flexors of the musician’s hand: theoretical analysis, clinical examples. J Biomech 30: 873–882, 1997. doi:10.1016/s0021-9290(97)00045-6. 9302609

[49] Hartman CG, Straus WL. The Anatomy of the Rhesus Monkey (Macaca mulatta). Baltimore, MD: The Williams and Wilkins Company, 1933.

[50] Serlin DM, Schieber MH. Morphologic regions of the multitendoned extrinsic finger muscles in the monkey forearm. Acta Anat (Basel) 146: 255–266, 1993. doi:10.1159/000147465. 8317203

[51] Marzke MW. Evolutionary development of the human thumb. Hand Clin 8: 1–8, 1992. 1572915

[52] Linburg RM, Comstock BE. Anomalous tendon slips from the flexor pollicis longus to the flexor digitorum profundus. J Hand Surg Am 4: 79–83, 1979.doi:10.1016/s0363-5023(79)80110-0. 759509

[53] Bernstein N. The Coordination and Regulation of Movements. Oxford, UK: Permagon, 1967.

[54] Beevor CE. (1) The Croonian Lectures on Muscular Movements and (2) Remarks on Paralysis of the Movements of the Trunk in Hemiplegia. New York: The MacMillan Company, 1903, p. 1–79.

[55] Schieber MH. Muscular production of individuated finger movements: the roles of extrinsic finger muscles. J Neurosci 15: 284–297, 1995. doi:10.1523/JNEUROSCI.15-01-00284.1995. 7823134 PMC6578320

[56] Reilly KT, Schieber MH. Incomplete functional subdivision of the human multitendoned finger muscle flexor digitorum profundus: an electromyographic study. J Neurophysiol 90: 2560–2570, 2003. doi:10.1152/jn.00287.2003. 12815024

[57] Birdwell JA, Hargrove LJ, Kuiken TA, Weir RF. Activation of individual extrinsic thumb muscles and compartments of extrinsic finger muscles. J Neurophysiol 110: 1385–1392, 2013. doi:10.1152/jn.00748.2012. 23803329 PMC3763151

[58] Schieber MH, Chua M, Petit J, Hunt CC. Tension distribution of single motor units in multitendoned muscles: comparison of a homologous digit muscle in cats and monkeys. J Neurosci 17: 1734–1747, 1997. doi:10.1523/JNEUROSCI.17-05-01734.1997. 9030632 PMC6573362

[59] Keen DA, Fuglevand AJ. Distribution of motor unit force in human extensor digitorum assessed by spike-triggered averaging and intraneural microstimulation. J Neurophysiol 91: 2515–2523, 2004. doi:10.1152/jn.01178.2003. 14724266

[60] Schieber MH, Gardinier J, Liu J. Tension distribution to the five digits of the hand by neuromuscular compartments in the macaque flexor digitorum profundus. J Neurosci 21: 2150–2158, 2001. doi:10.1523/JNEUROSCI.21-06-02150.2001. 11245699 PMC6762629

[61] Schieber MH. Electromyographic evidence of two functional subdivisions in the rhesus monkey’s flexor digitorum profundus. Exp Brain Res 95: 251–260, 1993. doi:10.1007/BF00229783. 8224050

[62] Kilbreath SL, Gorman RB, Raymond J, Gandevia SC. Distribution of the forces produced by motor unit activity in the human flexor digitorum profundus. J Physiol 543: 289–296, 2002. doi:10.1113/jphysiol.2002.023861. 12181299 PMC2290486

[63] Takei T, Confais J, Tomatsu S, Oya T, Seki K. Neural basis for hand muscle synergies in the primate spinal cord. Proc Natl Acad Sci USA 114: 8643–8648, 2017. doi:10.1073/pnas.1704328114. 28739958 PMC5559022

[64] Bremner FD, Baker JR, Stephens JA. Correlation between the discharges of motor units recorded from the same and from different finger muscles in man. J Physiol 432: 355–380, 1991. doi:10.1113/jphysiol.1991.sp018389. 1886059 PMC1181330

[65] Fetz EE, Cheney PD. Postspike facilitation of forelimb muscle activity by primate corticomotoneuronal cells. J Neurophysiol 44: 751–772, 1980. doi:10.1152/jn.1980.44.4.751. 6253604

[66] Fetz EE, Cheney PD, German DC. Corticomotoneuronal connections of precentral cells detected by postspike averages of EMG activity in behaving monkeys. Brain Res 114: 505–510, 1976. doi:10.1016/0006-8993(76)90973-2. 821592

[67] Lemon RN, Mantel GW, Muir RB. Corticospinal facilitation of hand muscles during voluntary movement in the conscious monkey. J Physiol 381: 497–527, 1986. doi:10.1113/jphysiol.1986.sp016341. 3625543 PMC1182993

[68] Baker SN, Lemon RN. Computer simulation of post-spike facilitation in spike-triggered averages of rectified EMG. J Neurophysiol 80: 1391–1406, 1998. doi:10.1152/jn.1998.80.3.1391. 9744948

[69] McKiernan BJ, Marcario JK, Karrer JH, Cheney PD. Corticomotoneuronal postspike effects in shoulder, elbow, wrist, digit, and intrinsic hand muscles during a reach and prehension task. J Neurophysiol 80: 1961–1980, 1998. doi:10.1152/jn.1998.80.4.1961. 9772253

[70] Schieber MH, Rivlis G. A spectrum from pure post-spike effects to synchrony effects in spike-triggered averages of electromyographic activity during skilled finger movements. J Neurophysiol 94: 3325–3341, 2005. doi:10.1152/jn.00007.2005. 16014801

[71] Perlmutter SI, Maier MA, Fetz EE. Activity of spinal interneurons and their effects on forearm muscles during voluntary wrist movements in the monkey. J Neurophysiol 80: 2475–2494, 1998. doi:10.1152/jn.1998.80.5.2475. 9819257

[72] Takei T, Seki K. Spinal interneurons facilitate coactivation of hand muscles during a precision grip task in monkeys. J Neurosci 30: 17041–17050, 2010. doi:10.1523/JNEUROSCI.4297-10.2010. 21159974 PMC6634901

[73] Davidson AG, Schieber MH, Buford JA. Bilateral spike-triggered average effects in arm and shoulder muscles from the monkey pontomedullary reticular formation. J Neurosci 27: 8053–8058, 2007. doi:10.1523/JNEUROSCI.0040-07.2007. 17652596 PMC6672715

[74] Mewes K, Cheney PD. Facilitation and suppression of wrist and digit muscles from single rubromotoneuronal cells in the awake monkey. J Neurophysiol 66: 1965–1977, 1991. doi:10.1152/jn.1991.66.6.1965. 1812229

[75] Datta AK, Stephens JA. Synchronization of motor unit activity during voluntary contraction in man. J Physiol 422: 397–419, 1990. doi:10.1113/jphysiol.1990.sp017991. 2352185 PMC1190139

[76] Nordstrom MA, Fuglevand AJ, Enoka RM. Estimating the strength of common input to human motoneurons from the cross-correlogram. J Physiol 453: 547–574, 1992. doi:10.1113/jphysiol.1992.sp019244. 1464844 PMC1175573

[77] Farmer SF, Swash M, Ingram DA, Stephens JA. Changes in motor unit synchronization following central nervous lesions in man. J Physiol 463: 83–105, 1993. doi:10.1113/jphysiol.1993.sp019585. 8246205 PMC1175334

[78] Bremner FD, Baker JR, Stephens JA. Variation in the degree of synchronization exhibited by motor units lying in different finger muscles in man. J Physiol 432: 381–399, 1991. doi:10.1113/jphysiol.1991.sp018390. 1886060 PMC1181331

[79] Keen DA, Fuglevand AJ. Common input to motor neurons innervating the same and different compartments of the human extensor digitorum muscle. J Neurophysiol 91: 57–62, 2004. doi:10.1152/jn.00650.2003. 12968013

[80] Reilly KT, Nordstrom MA, Schieber MH. Short-term synchronization between motor units in different functional subdivisions of the human flexor digitorum profundus muscle. J Neurophysiol 92: 734–742, 2004. doi:10.1152/jn.00027.2004. 15056692

[81] Winges SA, Santello M. Common input to motor units of digit flexors during multi-digit grasping. J Neurophysiol 92: 3210–3220, 2004. doi:10.1152/jn.00516.2004. 15240764

[82] McIsaac TL, Fuglevand AJ. Motor-unit synchrony within and across compartments of the human flexor digitorum superficialis. J Neurophysiol 97: 550–556, 2007. doi:10.1152/jn.01071.2006. 17093112

[83] McIsaac TL, Fuglevand AJ. Common synaptic input across motor nuclei supplying intrinsic muscles involved in the precision grip. Exp Brain Res 188: 159–164, 2008. doi:10.1007/s00221-008-1432-7. 18506431 PMC5792194

[84] Butler TJ, Kilbreath SL, Gorman RB, Gandevia SC. Selective recruitment of single motor units in human flexor digitorum superficialis muscle during flexion of individual fingers. J Physiol 567: 301–309, 2005. doi:10.1113/jphysiol.2005.089201. 15946972 PMC1474175

[85] Kilbreath SL, Gandevia SC. Limited independent flexion of the thumb and fingers in human subjects. J Physiol 479: 487–497, 1994. doi:10.1113/jphysiol.1994.sp020312. 7837104 PMC1155766

[86] van Duinen H, Yu WS, Gandevia SC. Limited ability to extend the digits of the human hand independently with extensor digitorum. J Physiol 587: 4799–4810, 2009. doi:10.1113/jphysiol.2009.177964. 19703966 PMC2770148

[87] Schieber MH, Reilly KT, Lang CE. Motor cortex control of a complex peripheral apparatus: the neuromuscular evolution of individuated finger movements. In: *Frontiers in Neuroscience. Motor Cortex in Voluntary Movements: a Distributed System for Distributed Functions*, edited by Riehle A, Vaadia E. Boca Raton, FL: CRC Press, 2005, p. 87–107.

[88] Kuypers HG. A new look at the organization of the motor system. Progr Brain Res 57: 381–403, 1982. doi:10.1016/S0079-6123(08)64138-2. . 6818612

[89] Kuypers HG. Some aspects of the organization of the output of the motor cortex. In: *Ciba Foundation. Motor Areas of the Cerebral Cortex*, edited by Bock GR, O’Connor M, Marsh J. Sussex, UK: John Wiley & Sons, 1987, p. 63–82. 10.1002/9780470513545.ch5 3322721

[90] Nathan PW, Smith MC. The rubrospinal and central tegmental tracts in man. Brain 105: 223–269, 1982. doi:10.1093/brain/105.2.223. 7082990

[91] Shapovalov AV, Gokin AP, Piliavskiĭ AI, Enin LD, Zaval’naia NI. [Rubrofugal influences on the spike activity of neurons of the medial reticulo-spinal tract]. Neirofiziologiia 7: 533–540, 1975. 1207836

[92] Illert M, Lundberg A, Tanaka R. Integration in descending motor pathways controlling the forelimb in the cat. 3. Convergence on propriospinal neurones transmitting disynaptic excitation from the corticospinal tract and other descending tracts. Exp Brain Res 29: 323–346, 1977. doi:10.1007/BF00236174. 913521

[93] Illert M, Jankowska E, Lundberg A, Odutola A. Integration in descending motor pathways controlling the forelimb in the cat. 7. Effects from the reticular formation on C3-C4 propriospinal neurones. Exp Brain Res 42: 269–281, 1981. doi:10.1007/BF00237494. 7238671

[94] Bortoff GA, Strick PL. Corticospinal terminations in two new-world primates: further evidence that corticomotoneuronal connections provide part of the neural substrate for manual dexterity. J Neurosci 13: 5105–5118, 1993. doi:10.1523/JNEUROSCI.13-12-05105.1993. 7504721 PMC6576412

[95] Ivanco TL, Pellis SM, Whishaw IQ. Skilled forelimb movements in prey catching and in reaching by rats (Rattus norvegicus) and opossums (Monodelphis domestica): relations to anatomical differences in motor systems. Behav Brain Res 79: 163–181, 1996. doi:10.1016/0166-4328(96)00011-3. 8883828

[96] Kondo T, Yoshihara Y, Yoshino-Saito K, Sekiguchi T, Kosugi A, Miyazaki Y, Nishimura Y, Okano HJ, Nakamura M, Okano H, Isa T, Ushiba J. Histological and electrophysiological analysis of the corticospinal pathway to forelimb motoneurons in common marmosets. Neurosci Res 98: 35–44, 2015. doi:10.1016/j.neures.2015.05.001. 26093181

[97] Nudo RJ, Jenkins WM, Merzenich MM, Prejean T, Grenda R. Neurophysiological correlates of hand preference in primary motor cortex of adult squirrel monkeys. J Neurosci 12: 2918–2947, 1992. doi:10.1523/JNEUROSCI.12-08-02918.1992. 1494940 PMC6575643

[98] Sybirska E, Gorska T. Effects of red nucleus lesions on forelimb movements in the cat. Acta Neurobiol Exp (Wars) 40: 821–841, 1980. 7234514

[99] Song J, Pallucchi I, Ausborn J, Ampatzis K, Bertuzzi M, Fontanel P, Picton LD, El Manira A. Multiple rhythm-generating circuits act in tandem with pacemaker properties to control the start and speed of locomotion. Neuron 105: 1048–1061, 2020. doi:10.1016/j.neuron.2019.12.030. 31982322

[100] Stein PS. Neuronal control of turtle hindlimb motor rhythms. J Comp Physiol A Neuroethol Sens Neural Behav Physiol 191: 213–229, 2005. doi:10.1007/s00359-004-0568-6. 15452660

[101] Riddle CN, Baker SN. Convergence of pyramidal and medial brain stem descending pathways onto macaque cervical spinal interneurons. J Neurophysiol 103: 2821–2832, 2010. doi:10.1152/jn.00491.2009. 20457863 PMC2867561

[102] Takei T, Seki K. Spinal premotor interneurons mediate dynamic and static motor commands for precision grip in monkeys. J Neurosci 33: 8850–8860, 2013. doi:10.1523/JNEUROSCI.4032-12.2013. 23678127 PMC6618822

[103] Alstermark B, Pettersson LG, Nishimura Y, Yoshino-Saito K, Tsuboi F, Takahashi M, Isa T. Motor command for precision grip in the macaque monkey can be mediated by spinal interneurons. J Neurophysiol 106: 122–126, 2011. doi:10.1152/jn.00089.2011. 21511706

[104] Sasaki S, Isa T, Pettersson LG, Alstermark B, Naito K, Yoshimura K, Seki K, Ohki Y. Dexterous finger movements in primate without monosynaptic corticomotoneuronal excitation. J Neurophysiol 92: 3142–3147, 2004. doi:10.1152/jn.00342.2004. 15175371

[105] Sugiyama Y, Higo N, Yoshino-Saito K, Murata Y, Nishimura Y, Oishi T, Isa T. Effects of early versus late rehabilitative training on manual dexterity after corticospinal tract lesion in macaque monkeys. J Neurophysiol 109: 2853–2865, 2013. doi:10.1152/jn.00814.2012. 23515793

[106] Kinoshita M, Matsui R, Kato S, Hasegawa T, Kasahara H, Isa K, Watakabe A, Yamamori T, Nishimura Y, Alstermark B, Watanabe D, Kobayashi K, Isa T. Genetic dissection of the circuit for hand dexterity in primates. Nature 487: 235–238, 2012. doi:10.1038/nature11206. 22722837

[107] Pierrot-Deseilligny E. Propriospinal transmission of part of the corticospinal excitation in humans. Muscle Nerve 26: 155–172, 2002. doi:10.1002/mus.1240. 12210379

[108] Pierrot-Deseilligny E. Transmission of the cortical command for human voluntary movement through cervical propriospinal premotoneurons. Prog Neurobiol 48: 489–517, 1996. doi:10.1016/0301-0082(96)00002-0. 8804118

[109] Brownstone RM, Chopek JW. Reticulospinal systems for tuning motor commands. Front Neural Circuits 12: 30, 2018. doi:10.3389/fncir.2018.00030. 29720934 PMC5915564

[110] Fregosi M, Contestabile A, Hamadjida A, Rouiller EM. Corticobulbar projections from distinct motor cortical areas to the reticular formation in macaque monkeys. Eur J Neurosci 45: 1379–1395, 2017. doi:10.1111/ejn.13576. 28394483

[111] Keizer K, Kuypers HG. Distribution of corticospinal neurons with collaterals to the lower brain stem reticular formation in monkey (Macaca fascicularis). Exp Brain Res 74: 311–318, 1989. doi:10.1007/BF00248864. 2924851

[112] Kuypers HG, Fleming WR, Farinholt JW. Subcorticospinal projections in the rhesus monkey. J Comp Neurol 118: 107–137, 1962. doi:10.1002/cne.901180109. 14461005

[113] Sakai ST, Davidson AG, Buford JA. Reticulospinal neurons in the pontomedullary reticular formation of the monkey (Macaca fascicularis). Neuroscience 163: 1158–1170, 2009. doi:10.1016/j.neuroscience.2009.07.036. 19631726 PMC2760615

[114] Riddle CN, Edgley SA, Baker SN. Direct and indirect connections with upper limb motoneurons from the primate reticulospinal tract. J Neurosci 29: 4993–4999, 2009. doi:10.1523/JNEUROSCI.3720-08.2009. 19369568 PMC2690979

[115] Buford JA, Davidson AG. Movement-related and preparatory activity in the reticulospinal system of the monkey. Exp Brain Res 159: 284–300, 2004. doi:10.1007/s00221-004-1956-4. 15221165

[116] Soteropoulos DS, Williams ER, Baker SN. Cells in the monkey ponto-medullary reticular formation modulate their activity with slow finger movements. J Physiol 590: 4011–4027, 2012. doi:10.1113/jphysiol.2011.225169. 22641776 PMC3476645

[117] Matsuyama K, Mori F, Kuze B, Mori S. Morphology of single pontine reticulospinal axons in the lumbar enlargement of the cat: a study using the anterograde tracer PHA-L. J Comp Neurol 410: 413–430, 1999. [CrossR] doi:10.1002/(SICI)1096-9861(19990802)410:3<413::AID-CNE5>3.0.CO;2-Q. 10404409

[118] Matsuyama K, Takakusaki K, Nakajima K, Mori S. Multi-segmental innervation of single pontine reticulospinal axons in the cervico-thoracic region of the cat: anterograde PHA-L tracing study. J Comp Neurol 377: 234–250, 1997. doi:10.1002/(SICI)1096-9861(19970113)377:2<234::AID-CNE6>3.0.CO;2-4. 8986883

[119] Nathan PW, Smith M, Deacon P. Vestibulospinal, reticulospinal and descending propriospinal nerve fibres in man. Brain 119: 1809–1833, 1996. doi:10.1093/brain/119.6.1809. 9009990

[120] Davidson AG, Buford JA. Motor outputs from the primate reticular formation to shoulder muscles as revealed by stimulus-triggered averaging. J Neurophysiol 92: 83–95, 2004. doi:10.1152/jn.00083.2003. 15014106 PMC2740726

[121] Fisher KM, Zaaimi B, Edgley SA, Baker SN. Extensive cortical convergence to primate reticulospinal pathways. J Neurosci 41: 1005–1018, 2021. doi:10.1523/JNEUROSCI.1379-20.2020. 33268548 PMC7880280

[122] Zaaimi B, Dean LR, Baker SN. Different contributions of primary motor cortex, reticular formation, and spinal cord to fractionated muscle activation. J Neurophysiol 119: 235–250, 2018. doi:10.1152/jn.00672.2017. 29046427 PMC5866475

[123] Rothwell JC. The startle reflex, voluntary movement, and the reticulospinal tract. Suppl Clin Neurophysiol 58: 223–231, 2006. doi:10.1016/s1567-424x(09)70071-6. 16623334

[124] Honeycutt CF, Kharouta M, Perreault EJ. Evidence for reticulospinal contributions to coordinated finger movements in humans. J Neurophysiol 110: 1476–1483, 2013. doi:10.1152/jn.00866.2012. 23825395 PMC4042417

[125] Tazoe T, Perez MA. Cortical and reticular contributions to human precision and power grip. J Physiol 595: 2715–2730, 2017. doi:10.1113/JP273679. 27891607 PMC5390869

[126] ten Donkelaar HJ. Evolution of the red nucleus and rubrospinal tract. Behav Brain Res 28: 9–20, 1988. doi:10.1016/0166-4328(88)90072-1. 3289562

[127] Humphrey DR, Gold R, Reed DJ. Sizes, laminar and topographic origins of cortical projections to the major divisions of the red nucleus in the monkey. J Comp Neurol 225: 75–94, 1984. doi:10.1002/cne.902250109. 6725640

[128] Sinopoulou E, Rosenzweig ES, Conner JM, Gibbs D, Weinholtz CA, Weber JL, Brock JH, Nout-Lomas YS, Ovruchesky E, Takashima Y, Biane JS, Kumamaru H, Havton LA, Beattie MS, Bresnahan JC, Tuszynski MH. Rhesus macaque versus rat divergence in the corticospinal projectome. Neuron 110: 2970–2983, 2022. doi:10.1016/j.neuron.2022.07.002. 35917818 PMC9509478

[129] Steward O, Yee KM, Metcalfe M, Willenberg R, Luo J, Azevedo R, Martin-Thompson JH, Gandhi SP. Rostro-Caudal Specificity of Corticospinal Tract Projections in Mice. Cereb Cortex 31: 2322–2344, 2021. doi:10.1093/cercor/bhaa338. 33350438 PMC8023844

[130] Lassek AM. The Pyramidal Tract. Its Status in Medicine. Springfield, IL: Thomas, 1954.

[131] Heffner R, Masterton B. Variation in form of the pyramidal tract and its relationship to digital dexterity. Brain Behav Evol 12: 161–200, 1975. doi:10.1159/000124401. 1212616

[132] Dum RP, Strick PL. The origin of corticospinal projections from the premotor areas in the frontal lobe. J Neurosci 11: 667–689, 1991. doi:10.1523/JNEUROSCI.11-03-00667.1991. 1705965 PMC6575356

[133] Galea MP, Darian-Smith I. Multiple corticospinal neuron populations in the macaque monkey are specified by their unique cortical origins, spinal terminations, and connections. Cereb Cortex 4: 166–194, 1994. doi:10.1093/cercor/4.2.166. 8038567

[134] He SQ, Dum RP, Strick PL. Topographic organization of corticospinal projections from the frontal lobe: motor areas on the lateral surface of the hemisphere. J Neurosci 13: 952–980, 1993. doi:10.1523/JNEUROSCI.13-03-00952.1993. 7680069 PMC6576595

[135] He SQ, Dum RP, Strick PL. Topographic organization of corticospinal projections from the frontal lobe: motor areas on the medial surface of the hemisphere. J Neurosci 15: 3284–3306, 1995. doi:10.1523/JNEUROSCI.15-05-03284.1995. 7538558 PMC6578253

[136] Morecraft RJ, Ge J, Stilwell-Morecraft KS, McNeal DW, Pizzimenti MA, Darling WG. Terminal distribution of the corticospinal projection from the hand/arm region of the primary motor cortex to the cervical enlargement in rhesus monkey. J Comp Neurol 521: 4205–4235, 2013. doi:10.1002/cne.23410. 23840034 PMC3894926

[137] Morecraft RJ, Ge J, Stilwell-Morecraft KS, Rotella DL, Pizzimenti MA, Darling WG. Terminal organization of the corticospinal projection from the lateral premotor cortex to the cervical enlargement (C5-T1) in rhesus monkey. J Comp Neurol 527: 2761–2789, 2019. doi:10.1002/cne.24706. 31032921 PMC6721988

[138] Nudo RJ, Masterton RB. Descending pathways to the spinal cord, III: Sites of origin of the corticospinal tract. J Comp Neurol 296: 559–583, 1990. doi:10.1002/cne.902960405. 2113540

[139] Cheney PD, Fetz EE, Palmer SS. Patterns of facilitation and suppression of antagonist forelimb muscles from motor cortex sites in the awake monkey. J Neurophysiol 53: 805–820, 1985. doi:10.1152/jn.1985.53.3.805. 2984355

[140] Yousry TA, Schmid UD, Alkadhi H, Schmidt D, Peraud A, Buettner A, Winkler P. Localization of the motor hand area to a knob on the precentral gyrus - a new landmark. Brain 120 (Pt 1): 141–157, 1997. doi:10.1093/brain/120.1.141. 9055804

[141] White LE, Andrews TJ, Hulette C, Richards A, Groelle M, Paydarfar J, Purves D. Structure of the human sensorimotor system. I: Morphology and cytoarchitecture of the central sulcus.Cereb Cortex 7: 18–30, 1997. doi:10.1093/cercor/7.1.18. . 9023429

[142] Lemon R. Recent advances in our understanding of the primate corticospinal system. F1000Res 8: F1000, 2019. doi:10.12688/f1000research.17445.1. 30906528 PMC6415323

[143] de Noordhout AM, Rapisarda G, Bogacz D, Gerard P, De Pasqua V, Pennisi G, Delwaide PJ. Corticomotoneuronal synaptic connections in normal man: an electrophysiological study. Brain 122: 1327–1340, 1999. doi:10.1093/brain/122.7.1327. 10388798

[144] Palmer E, Ashby P. Corticospinal projections to upper limb motoneurones in humans. J Physiol 448: 397–412, 1992. doi:10.1113/jphysiol.1992.sp019048. 1593472 PMC1176206

[145] Rathelot JA, Strick PL. Subdivisions of primary motor cortex based on cortico-motoneuronal cells. Proc Natl Acad Sci U S A 106: 918–923, 2009. doi:10.1073/pnas.0808362106. 19139417 PMC2621250

[146] Witham CL, Fisher KM, Edgley SA, Baker SN. Corticospinal inputs to primate motoneurons innervating the forelimb from two divisions of primary motor cortex and area 3a. J Neurosci 36: 2605–2616, 2016. doi:10.1523/JNEUROSCI.4055-15.2016. 26937002 PMC4879208

[147] Geyer S, Ledberg A, Schleicher A, Kinomura S, Schormann T, Burgel U, Klingberg T, Larsson J, Zilles K, Roland PE. Two different areas within the primary motor cortex of man. Nature 382: 805–807, 1996. doi:10.1038/382805a0. 8752272

[148] Griffin DM, Strick PL. The motor cortex uses active suppression to sculpt movement. Sci Adv 6: eabb8395, 2020. doi:10.1126/sciadv.abb8395. 32937371 PMC7442473

[149] Schieber MH. How might the motor cortex individuate movements? Trends Neurosci 13: 440–445, 1990. doi:10.1016/0166-2236(90)90093-p. 1701575

[150] Andersen P, Hagan PJ, Phillips CG, Powell TP. Mapping by microstimulation of overlapping projections from area 4 to motor units of the baboon’s hand. Proc R Soc Lond B Biol Sci 188: 31–36, 1975. doi:10.1098/rspb.1975.0002. 234617

[151] Rathelot JA, Strick PL. Muscle representation in the macaque motor cortex: an anatomical perspective. Proc Natl Acad Sci U S A 103: 8257–8262, 2006. doi:10.1073/pnas.0602933103. 16702556 PMC1461407

[152] Huntley GW, Jones EG. Relationship of intrinsic connections to forelimb movement representations in monkey motor cortex: a correlative anatomic and physiological study. J Neurophysiol 66: 390–413, 1991. doi:10.1152/jn.1991.66.2.390. 1723093

[153] Shinoda Y, Zarzecki P, Asanuma H. Spinal branching of pyramidal tract neurons in the monkey. Exp Brain Res 34: 59–72, 1979. doi:10.1007/BF00238341. 103741

[154] Buys EJ, Lemon RN, Mantel GW, Muir RB. Selective facilitation of different hand muscles by single corticospinal neurones in the conscious monkey. J Physiol 381: 529–549, 1986. doi:10.1113/jphysiol.1986.sp016342. 3625544 PMC1182994

[155] Griffin DM, Hoffman DS, Strick PL. Corticomotoneuronal cells are “functionally tuned.” Science 350: 667–670, 2015. doi:10.1126/science.aaa8035. 26542568 PMC4829105

[156] Schieber MH, Hibbard LS. How somatotopic is the motor cortex hand area? Science 261: 489–492, 1993. doi:10.1126/science.8332915. 8332915

[157] Arbuckle SA, Weiler J, Kirk EA, Rice CL, Schieber M, Pruszynski JA, Ejaz N, Diedrichsen J. Structure of population activity in primary motor cortex for single finger flexion and extension. J Neurosci 40: 9210–9223, 2020. doi:10.1523/JNEUROSCI.0999-20.2020. 33087474 PMC7687056

[158] Ejaz N, Hamada M, Diedrichsen J. Hand use predicts the structure of representations in sensorimotor cortex. Nat Neurosci 18: 1034–1040, 2015. doi:10.1038/nn.4038. 26030847

[159] Huber L, Finn ES, Handwerker DA, Bonstrup M, Glen DR, Kashyap S, Ivanov D, Petridou N, Marrett S, Goense J, Poser BA, Bandettini PA. Sub-millimeter fMRI reveals multiple topographical digit representations that form action maps in human motor cortex. Neuroimage 208: 116463, 2020. doi:10.1016/j.neuroimage.2019.116463. 31862526 PMC11829252

[160] Schellekens W, Petridou N, Ramsey NF. Detailed somatotopy in primary motor and somatosensory cortex revealed by Gaussian population receptive fields. Neuroimage 179: 337–347, 2018. doi:10.1016/j.neuroimage.2018.06.062. 29940282 PMC6413921

[161] Poliakov AV, Schieber MH. Limited functional grouping of neurons in the motor cortex hand area during individuated finger movements: a cluster analysis. J Neurophysiol 82: 3488–3505, 1999. doi:10.1152/jn.1999.82.6.3488. 10601477

[162] Overduin SA, d’Avella A, Roh J, Carmena JM, Bizzi E. Representation of muscle synergies in the primate brain. J Neurosci 35: 12615–12624, 2015. 26377453 10.1523/JNEUROSCI.4302-14.2015 PMC4571600

[163] Jackson A, Gee VJ, Baker SN, Lemon RN. Synchrony between neurons with similar muscle fields in monkey motor cortex. Neuron 38: 115–125, 2003. doi:10.1016/s0896-6273(03)00162-4. 12691669

[164] Smith WS, Fetz EE. Synaptic interactions between forelimb-related motor cortex neurons in behaving primates. J Neurophysiol 102: 1026–1039, 2009. doi:10.1152/jn.91051.2008. 19439672 PMC2724363

[165] Smith WS, Fetz EE. Synaptic linkages between corticomotoneuronal cells affecting forelimb muscles in behaving primates. J Neurophysiol 102: 1040–1048, 2009. doi:10.1152/jn.91052.2008. 19515946 PMC2724364

[166] Griffin DM, Hudson HM, Belhaj-Saif A, McKiernan BJ, Cheney PD. Do corticomotoneuronal cells predict target muscle EMG activity? J Neurophysiol 99: 1169–1986, 2008. doi:10.1152/jn.00906.2007. 18160426

[167] Schieber MH, Rivlis G. Partial reconstruction of muscle activity from a pruned network of diverse motor cortex neurons. J Neurophysiol 97: 70–82, 2007. doi:10.1152/jn.00544.2006. 17035361

[168] Schieber MH. Dissociating motor cortex from the motor. J Physiol 589: 5613–5624, 2011. doi:10.1113/jphysiol.2011.215814. 22005673 PMC3249037

[169] Ulfhake B, Cullheim S. Postnatal development of cat hind limb motoneurons. III: Changes in size of motoneurons supplying the triceps surae muscle. J Comp Neurol 278: 103–120, 1988. doi:10.1002/cne.902780107. 3209749

[170] Davidson AG, Chan V, O’Dell R, Schieber MH. Rapid changes in throughput from single motor cortex neurons to muscle activity. Science 318: 1934–1937, 2007. doi:10.1126/science.1149774. 18096808

[171] Sohn YH, Hallett M. Surround inhibition in human motor system. Exp Brain Res 158: 397–404, 2004. doi:10.1007/s00221-004-1909-y. 15146307

[172] Stinear CM, Byblow WD. Role of intracortical inhibition in selective hand muscle activation. J Neurophysiol 89: 2014–2020, 2003. doi:10.1152/jn.00925.2002. 12611950

[173] Beck S, Hallett M. Surround inhibition is modulated by task difficulty. Clin Neurophysiol 121: 98–103, 2010. doi:10.1016/j.clinph.2009.09.010. 19906559 PMC2818458

[174] Shin HW, Sohn YH, Hallett M. Hemispheric asymmetry of surround inhibition in the human motor system. Clin Neurophysiol 120: 816–819, 2009. doi:10.1016/j.clinph.2009.02.004. 19299196

[175] Leodori G, Thirugnanasambandam N, Conn H, Popa T, Berardelli A, Hallett M. Intracortical inhibition and surround inhibition in the motor cortex: a TMS-EEG study. Front Neurosci 13: 612, 2019. doi:10.3389/fnins.2019.00612. 31249507 PMC6582275

[176] Jenny AB, Inukai J. Principles of motor organization of the monkey cervical spinal cord. J Neurosci 3: 567–575, 1983. doi:10.1523/JNEUROSCI.03-03-00567.1983. 6827309 PMC6564558

[177] Dum RP, Strick PL. Frontal lobe inputs to the digit representations of the motor areas on the lateral surface of the hemisphere. J Neurosci 25: 1375–1386, 2005. doi:10.1523/JNEUROSCI.3902-04.2005. 15703391 PMC6726000

[178] Bostan AC, Strick PL. The basal ganglia and the cerebellum: nodes in an integrated network. Nat Rev Neurosci 19: 338–350, 2018. doi:10.1038/s41583-018-0002-7. 29643480 PMC6503669

[179] Holsapple JW, Preston JB, Strick PL. The origin of thalamic inputs to the “hand” representation in the primary motor cortex. J Neurosci 11: 2644–2654, 1991. doi:10.1523/JNEUROSCI.11-09-02644.1991. 1715388 PMC6575258

[180] Kim JS. Predominant involvement of a particular group of fingers due to small, cortical infarction. Neurology 56: 1677–1682, 2001. doi:10.1212/wnl.56.12.1677. 11425933

[181] Phan TG, Evans BA, Huston J. Pseudoulnar palsy from a small infarct of the precentral knob. Neurology 54: 2185, 2000. doi:10.1212/wnl.54.11.2185. 10851393

[182] Schieber MH. Somatotopic gradients in the distributed organization of the human primary motor cortex hand area: evidence from small infarcts. Exp Brain Res 128: 139–148, 1999. doi:10.1007/s002210050829. 10473752

[183] Takahashi N, Kawamura M, Araki S. Isolated hand palsy due to cortical infarction: Localization of the motor hand area. Neurology 58: 1412–1414, 2002. doi:10.1212/wnl.58.9.1412. 12011293

[184] Gordon EM, Chauvin RJ, Van AN, Rajesh A, Nielsen A, Newbold DJ, , et al. A somato-cognitive action network alternates with effector regions in motor cortex. Nature 617: 351–359, 2023., doi:10.1038/s41586-023-05964-2. 37076628 PMC10172144

[185] Schieber MH. Constraints on somatotopic organization in the primary motor cortex. J Neurophysiol 86: 2125–2143, 2001. doi:10.1152/jn.2001.86.5.2125. 11698506

[186] Angelaki DE, Soechting JF. Non-uniform temporal scaling of hand and finger kinematics during typing. Exp Brain Res 95: 319–329, 1993. doi:10.1007/BF00229789. 8224056

[187] Engel KC, Flanders M, Soechting JF. Anticipatory and sequential motor control in piano playing. Exp Brain Res 113: 189–199, 1997. doi:10.1007/BF02450317. 9063705

[188] Furuya S, Flanders M, Soechting JF. Hand kinematics of piano playing. J Neurophysiol 106: 2849–2864, 2011. doi:10.1152/jn.00378.2011. 21880938 PMC3234081

[189] Walshe FM. On the mode of representation of movements in the motor cortex, with special reference to “convulsions beginning unilaterally’’ (Jackson). Brain 66: 104–139, 1943. doi:10.1093/brain/66.2.104.

[190] Morasso P. Spatial control of arm movements. Exp Brain Res 42: 223–227, 1981. doi:10.1007/BF00236911. 7262217

[191] Shadmehr R, Wise SP. The Computational Neurobiology of Reaching and Pointing: a Foundation for Motor Learning. Cambridge, MA: The MIT Press, 2004.

[192] Charles S, Williams R. Measurement of hand dynamics in a microsurgery environment: Preliminary data in the design of a bimanual telemicro-operation test bed. In: *Proceedings of the NASA Conference on Space Telerobotics*. Pasadena, CA: Jet Propulsion Laboratory, 1989, p. 109–118.

[193] Sabatini AM, Bergamasco M, Dario P. Force feedback-based telemicromanipulation for robot surgery on soft tissues. In: *Proceedings of the Annual International Engineering in Medicine and Biology Society*. New York: IEEE, 1989, p. 890–891.

[194] Tan HZ, Durlach NI, Rabinowitz WM, Reed CM, Santos JR. Reception of Morse code through motional, vibrotactile, and auditory stimulation. Percept Psychophys 59: 1004–1017, 1997. doi:10.3758/bf03205516. 9360474

[195] Aoki T, Furuya S, Kinoshita H. Finger-tapping ability in male and female pianists and nonmusician controls. Motor Control 9: 23–39, 2005. doi:10.1123/mcj.9.1.23. 15784948

[196] Slobounov S, Chiang H, Johnston J, Ray W. Modulated cortical control of individual fingers in experienced musicians: an EEG study. Electroencephalographic study. Clin Neurophysiol 113: 2013–2024, 2002. doi:10.1016/s1388-2457(02)00298-5. 12464342

[197] Gaser C, Schlaug G. Brain structures differ between musicians and non-musicians. J Neurosci 23: 9240–9245, 2003. doi:10.1523/JNEUROSCI.23-27-09240.2003. 14534258 PMC6740845

[198] Furuya S, Altenmuller E. Flexibility of movement organization in piano performance. Front Hum Neurosci 7: 173, 2013. doi:10.3389/fnhum.2013.00173. 23882199 PMC3712142

[199] Furuya S, Nakamura A, Nagata N. Acquisition of individuated finger movements through musical practice. Neuroscience 275: 444–454, 2014. doi:10.1016/j.neuroscience.2014.06.031. 24973654

[200] Oku T, Furuya S. Skilful force control in expert pianists. Exp Brain Res 235: 1603–1615, 2017. doi:10.1007/s00221-017-4926-3. 28260157

[201] Furuya S, Soechting JF. Speed invariance of independent control of finger movements in pianists. J Neurophysiol 108: 2060–2068, 2012. doi:10.1152/jn.00378.2012. 22815403 PMC3545004

[202] Tominaga K, Lee A, Altenmuller E, Miyazaki F, Furuya S. Kinematic origins of motor inconsistency in expert pianists. PLoS One 11: e0161324, 2016. doi:10.1371/journal.pone.0161324. 27537686 PMC4990412

[203] Li ZM, Latash ML, Zatsiorsky VM. Force sharing among fingers as a model of the redundancy problem. Exp Brain Res 119: 276–286, 1998. doi:10.1007/s002210050343. 9551828

[204] Vaillancourt DE, Slifkin AB, Newell KM. Inter-digit individuation and force variability in the precision grip of young, elderly, and Parkinson’s disease participants. Motor Control 6: 113–128, 2002. doi:10.1123/mcj.6.2.113. 12122222

[205] Kim SW, Shim JK, Zatsiorsky VM, Latash ML. Anticipatory adjustments of multi-finger synergies in preparation for self-triggered perturbations. Exp Brain Res 174: 604–612, 2006. doi:10.1007/s00221-006-0505-8. 16724179

[206] Olafsdottir H, Yoshida N, Zatsiorsky VM, Latash ML. Anticipatory covariation of finger forces during self-paced and reaction time force production. Neurosci Lett 381: 92–96, 2005. doi:10.1016/j.neulet.2005.02.003. 15882796 PMC2827154

[207] Zhang W, Sainburg RL, Zatsiorsky VM, Latash ML. Hand dominance and multi-finger synergies. Neurosci Lett 409: 200–204, 2006. doi:10.1016/j.neulet.2006.09.048. 17018249 PMC1752208

[208] Latash ML, Scholz JF, Danion F, Schoner G. Finger coordination during discrete and oscillatory force production tasks. Exp Brain Res 146: 419–432, 2002. doi:10.1007/s00221-002-1196-4. 12355270

[209] Latash ML, Scholz JF, Danion F, Schoner G. Structure of motor variability in marginally redundant multifinger force production tasks. Exp Brain Res 141: 153–165, 2001. doi:10.1007/s002210100861. 11713627

[210] Rearick MP, Santello M. Force synergies for multifingered grasping: effect of predictability in object center of mass and handedness. Exp Brain Res 144: 38–49, 2002. doi:10.1007/s00221-002-1024-x. 11976758

[211] Karakostis FA, Haeufle D, Anastopoulou I, Moraitis K, Hotz G, Tourloukis V, Harvati K. Biomechanics of the human thumb and the evolution of dexterity. Curr Biol 31: 1317–1325, 2021. doi:10.1016/j.cub.2020.12.041. 33513351 PMC7987722

[212] Napier JR. Prehensility and opposability in the hands of primates. Symp Zool Soc Lond 5: 115–132, 1961.

[213] Napier JR. Studies of the hands of living primates. Proc Zool Soc Lond 134: 647–657, 1960. doi:10.1111/j.1469-7998.1960.tb05606.x.

[214] Cole KJ, Abbs JH. Coordination of three-joint digit movements for rapid finger-thumb grasp. J Neurophysiol 55: 1407–1423, 1986. doi:10.1152/jn.1986.55.6.1407. 3734863

[215] Mason CR, Gomez JE, Ebner TJ. Hand synergies during reach-to-grasp. J Neurophysiol 86: 2896–2910, 2001. doi:10.1152/jn.2001.86.6.2896. 11731546

[216] Santello M, Soechting JF. Matching object size by controlling finger span and hand shape. Somatosens Mot Res 14: 203–212, 1997. doi:10.1080/08990229771060. 9402650

[217] Zatsiorsky VM, Latash ML. Multifinger prehension: an overview. J Mot Behav 40: 446–476, 2008. doi:10.3200/JMBR.40.5.446-476. 18782719 PMC2659677

[218] Fu Q, Zhang W, Santello M. Anticipatory planning and control of grasp positions and forces for dexterous two-digit manipulation. J Neurosci 30: 9117–9126, 2010. doi:10.1523/JNEUROSCI.4159-09.2010. 20610745 PMC2917583

[219] Lukos J, Ansuini C, Santello M. Choice of contact points during multidigit grasping: effect of predictability of object center of mass location. J Neurosci 27: 3894–3903, 2007. doi:10.1523/JNEUROSCI.4693-06.2007. 17409254 PMC6672423

[220] Parikh PJ, Fine JM, Santello M. Dexterous object manipulation requires context-dependent sensorimotor cortical interactions in humans. Cereb Cortex 30: 3087–3101, 2020. doi:10.1093/cercor/bhz296. 31845726 PMC7197080

[221] Racz K, Brown D, Valero-Cuevas FJ. An involuntary stereotypical grasp tendency pervades voluntary dynamic multifinger manipulation. J Neurophysiol 108: 2896–2911, 2012. doi:10.1152/jn.00297.2012. 22956798 PMC3544870

[222] Winges SA, Soechting JF, Flanders M. Multidigit control of contact forces during transport of handheld objects. J Neurophysiol 98: 851–860, 2007. doi:10.1152/jn.00267.2007. 17553950

[223] Winges SA, Eonta SE, Soechting JF, Flanders M. Multi-digit control of contact forces during rotation of a hand-held object. J Neurophysiol 99: 1846–1856, 2008. doi:10.1152/jn.01238.2007. 18234979

[224] Liu X, Mosier KM, Mussa-Ivaldi FA, Casadio M, Scheidt RA. Reorganization of finger coordination patterns during adaptation to rotation and scaling of a newly learned sensorimotor transformation. J Neurophysiol 105: 454–473, 2011. doi:10.1152/jn.00247.2010. 20980541 PMC3023375

[225] Liu XL, Scheidt RA. Contributions of online visual feedback to the learning and generalization of novel finger coordination patterns. J Neurophysiol 99: 2546–2557, 2008. doi:10.1152/jn.01044.2007. 18353914

[226] Mosier KM, Scheidt RA, Acosta S, Mussa-Ivaldi FA. Remapping hand movements in a novel geometrical environment. J Neurophysiol 94: 4362–4372, 2005. doi:10.1152/jn.00380.2005. 16148276

[227] van Beek N, Stegeman DF, van den Noort JC, Hejv D, Maas H. Activity patterns of extrinsic finger flexors and extensors during movements of instructed and non-instructed fingers. J Electromyogr Kinesiol 38: 187–196, 2018. doi:10.1016/j.jelekin.2017.02.006. 28279574

[228] van den Noort JC, van Beek N, van der Kraan T, Veeger DH, Stegeman DF, Veltink PH, Maas H. Variable and asymmetric range of enslaving: fingers can act independently over small range of flexion. PLoS One 11: e0168636, 2016. doi:10.1371/journal.pone.0168636. 27992598 PMC5167409

[229] Baader AP, Kazennikov O, Wiesendanger M. Coordination of bowing and fingering in violin playing. Brain Res Cogn Brain Res 23: 436–443, 2005. doi:10.1016/j.cogbrainres.2004.11.008. 15820650

[230] Hore J, Watts S, Martin J, Miller B. Timing of finger opening and ball release in fast and accurate overarm throws. Exp Brain Res 103: 277–286, 1995. doi:10.1007/BF00231714. 7789435

[231] Hore J, Watts S, Tweed D. Errors in the control of joint rotations associated with inaccuracies in overarm throws. J Neurophysiol 75: 1013–1025, 1996. doi:10.1152/jn.1996.75.3.1013. 8867114

[232] Hore J, Watts S. Skilled throwers use physics to time ball release to the nearest millisecond. J Neurophysiol 106: 2024–2033, 2011. doi:10.1152/jn.00059.2011. 21775713

[233] Hore J, Watts S, Tweed D. Prediction and compensation by an internal model for back forces during finger opening in an overarm throw. J Neurophysiol 82: 1187–1197, 1999. doi:10.1152/jn.1999.82.3.1187. 10482738

[234] Ostry DJ, Gribble PL, Gracco VL. Coarticulation of jaw movements in speech production: Is context sensitivity in speech kinematics centrally planned? J Neurosci 16: 1570–1579, 1996. doi:10.1523/JNEUROSCI.16-04-01570.1996. 8778306 PMC6578564

[235] Winges SA, Furuya S, Faber NJ, Flanders M. Patterns of muscle activity for digital coarticulation. J Neurophysiol 110: 230–242, 2013. doi:10.1152/jn.00973.2012. 23596338 PMC3727042

[236] Jerde TE, Soechting JF, Flanders M. Coarticulation in fluent fingerspelling. J Neurosci 23: 2383–2393, 2003. doi:10.1523/JNEUROSCI.23-06-02383.2003. 12657698 PMC6742053

[237] Rajchert O, Ofir-Geva S, Melul Y, Khoury-Mireb M, Wonderman Bar-Sela O, Granot G, Caspi T, Frenkel Toledo S, Soroker N, Mawase F. Direction-dependent neural control of finger dexterity in humans. bioRxiv 04.25.538234, 2023. Doi:10.1101/2023.1104.1125.538234

[238] Twitchell TE. The restoration of motor function following hemiplegia in man. Brain 74: 443–480, 1951. doi:10.1093/brain/74.4.443. 14895765

[239] Tower SS. Pyramidal lesion in the monkey. Brain 63: 36–90, 1940. doi:10.1093/brain/63.1.36.

[240] Travis AM. Neurological deficiencies after ablation of the precentral motor area in macaca mulatta. Brain 78: 155–173, 1955. doi:10.1093/brain/78.2.155. 13239906

[241] Lawrence DG, Kuypers HG. The functional organization of the motor system of the monkey. I. The effects of bilateral pyramidal lesions. Brain 91: 1–14, 1968. doi:10.1093/brain/91.1.1. 4966862

[242] Hepp-Reymond MC, Wiesendanger M. Unilateral pyramidotomy in monkeys: effect on force and speed of a conditioned precision grip. Brain Res 36: 117–131, 1972. doi:10.1016/0006-8993(72)90770-6. 4621473

[243] Brochier T, Boudreau MJ, Par‚ M, Smith AM. The effects of muscimol inactivation of small regions of motor and somatosensory cortex on independent finger movements and force control in the precision grip. Exp Brain Res 128: 31–40, 1999. doi:10.1007/s002210050814. 10473737

[244] Matsumura M, Sawaguchi T, Oishi T, Ueki K, Kubota K. Behavioral deficits induced by local injection of bicuculline and muscimol into the primate motor and premotor cortex. J Neurophysiol 65: 1542–1553, 1991. doi:10.1152/jn.1991.65.6.1542. 1875261

[245] Schieber MH, Poliakov AV. Partial inactivation of the primary motor cortex hand area: effects on individuated finger movements. J Neurosci 18: 9038–9054, 1998. doi:10.1523/JNEUROSCI.18-21-09038.1998. 9787008 PMC6793546

[246] Xu J, Ejaz N, Hertler B, Branscheidt M, Widmer M, Faria AV, Harran MD, Cortes JC, Kim N, Celnik PA, Kitago T, Luft AR, Krakauer JW, Diedrichsen J. Separable systems for recovery of finger strength and control after stroke. J Neurophysiol 118: 1151–1163, 2017. doi:10.1152/jn.00123.2017. 28566461 PMC5547267

[247] Lang CE, Schieber MH. Differential impairment of individuated finger movements in humans after damage to the motor cortex or the corticospinal tract. J Neurophysiol 90: 1160–1170, 2003. doi:10.1152/jn.00130.2003. 12660350

[248] Li S, Latash ML, Yue GH, Siemionow V, Sahgal V. The effects of stroke and age on finger interaction in multi-finger force production tasks. Clin Neurophysiol 114: 1646–1655, 2003. doi:10.1016/s1388-2457(03)00164-0. 12948793

[249] Kamper DG, Fischer HC, Cruz EG, Rymer WZ. Weakness is the primary contributor to finger impairment in chronic stroke. Arch Phys Med Rehabil 87: 1262–1269, 2006. doi:10.1016/j.apmr.2006.05.013. 16935065

[250] Wolbrecht ET, Rowe JB, Chan V, Ingemanson ML, Cramer SC, Reinkensmeyer DJ. Finger strength, individuation, and their interaction: relationship to hand function and corticospinal tract injury after stroke. Clin Neurophysiol 129: 797–808, 2018. doi:10.1016/j.clinph.2018.01.057. 29453171 PMC5856636

[251] Colebatch JG, Gandevia SC. The distribution of muscular weakness in upper motor neuron lesions affecting the arm. Brain 112: 749–763, 1989. doi:10.1093/brain/112.3.749. 2731028

[252] Kamper DG, Harvey RL, Suresh S, Rymer WZ. Relative contributions of neural mechanisms versus muscle mechanics in promoting finger extension deficits following stroke. Muscle Nerve 28: 309–318, 2003. doi:10.1002/mus.10443. 12929190

[253] Lang CE, DeJong SL, Beebe JA. Recovery of thumb and finger extension and its relation to grasp performance after stroke. J Neurophysiol 102: 451–459, 2009. doi:10.1152/jn.91310.2008. 19458140 PMC2712280

[254] McPherson LM, Dewald JP. Differences between flexion and extension synergy-driven coupling at the elbow, wrist, and fingers of individuals with chronic hemiparetic stroke. Clin Neurophysiol 130: 454–468, 2019. doi:10.1016/j.clinph.2019.01.010. 30771722 PMC7856836

[255] Baker SN, Zaaimi B, Fisher KM, Edgley SA, Soteropoulos DS. Pathways mediating functional recovery. Prog Brain Res 218: 389–412, 2015. doi:10.1016/bs.pbr.2014.12.010. 25890147

[256] Clough JF, Kernell D, Phillips CG. The distribution of monosynaptic excitation from the pyramidal tract and from primary muscle spindle afferents to motoneurones of the baboon’s hand and forearm. J Physiol 198: 145–166, 1968. doi:10.1113/jphysiol.1968.sp008598. 16992310 PMC1365314

[257] Belhaj-Saif A, Cheney PD. Plasticity in the distribution of the red nucleus output to forearm muscles after unilateral lesions of the pyramidal tract. J Neurophysiol 83: 3147–3153, 2000. doi:10.1152/jn.2000.83.5.3147. 10805709

[258] Zaaimi B, Edgley SA, Soteropoulos DS, Baker SN. Changes in descending motor pathway connectivity after corticospinal tract lesion in macaque monkey. Brain 135: 2277–2289, 2012. doi:10.1093/brain/aws115. 22581799 PMC3381720

[259] Chapman CE, Wiesendanger M. Recovery of function following unilateral lesions of the bulbar pyramid in the monkey. Electroencephalogr Clin Neurophysiol 53: 374–387, 1982. doi:10.1016/0013-4694(82)90003-7. 6175500

[260] Gilman S, Marco LA. Effects of medullary pyramidotomy in the monkey. I. Clinical and electromyographic abnormalities. Brain 94: 495–514, 1971. doi:10.1093/brain/94.3.495. 5000052

[261] Schwartzman RJ. A behavioral analysis of complete unilateral section of the pyramidal tract at the medullary level in macaca mulatta. Ann Neurol 4: 234–244, 1978. doi:10.1002/ana.410040308. 102241

[262] Murata Y, Higo N, Oishi T, Yamashita A, Matsuda K, Hayashi M, Yamane S. Effects of motor training on the recovery of manual dexterity after primary motor cortex lesion in macaque monkeys. J Neurophysiol 99: 773–786, 2008. doi:10.1152/jn.01001.2007. 18094104

[263] Nudo RJ, Wise BM, Sifuentes F, Milliken GW. Neural substrates for the effects of rehabilitative training on motor recovery after ischemic infarct. Science 272: 1791–1794, 1996. doi:10.1126/science.272.5269.1791. 8650578

[264] Isa T. Dexterous hand movements and their recovery after central nervous system injury. Annu Rev Neurosci 42 42: 315–335, 2019. doi:10.1146/annurev-neuro-070918-050436. 30939102

[265] Isa T, Mitsuhashi M, Yamaguchi R. Alternative routes for recovery of hand functions after corticospinal tract injury in primates and rodents. Curr Opin Neurol 32: 836–843, 2019. doi:10.1097/WCO.0000000000000749. 31688166

[266] Nishimura Y, Morichika Y, Isa T. A subcortical oscillatory network contributes to recovery of hand dexterity after spinal cord injury. Brain 132: 709–721, 2009. doi:10.1093/brain/awn338. 19155271 PMC2664448

[267] Nishimura Y, Onoe H, Morichika Y, Perfiliev S, Tsukada H, Isa T. Time-dependent central compensatory mechanisms of finger dexterity after spinal cord injury. Science 318: 1150–1155, 2007. doi:10.1126/science.1147243. 18006750

[268] Fluet GG, Merians AS, Qiu Q, Davidow A, Adamovich SV. Comparing integrated training of the hand and arm with isolated training of the same effectors in persons with stroke using haptically rendered virtual environments, a randomized clinical trial. J Neuroeng Rehabil 11: 126, 2014. doi:10.1186/1743-0003-11-126. 25148846 PMC4156644

[269] Mawase F, Cherry-Allen K, Xu J, Anaya M, Uehara S, Celnik P. Pushing the rehabilitation boundaries: hand motor impairment can be reduced in chronic stroke. Neurorehabil Neural Repair 34: 733–745, 2020. doi:10.1177/1545968320939563. 32845230 PMC7457456

[270] Susanto EA, Tong RK, Ockenfeld C, Ho NS. Efficacy of robot-assisted fingers training in chronic stroke survivors: a pilot randomized-controlled trial. J Neuroeng Rehabil 12: 42, 2015. doi:10.1186/s12984-015-0033-5. 25906983 PMC4422529

[271] Thielbar KO, Lord TJ, Fischer HC, Lazzaro EC, Barth KC, Stoykov ME, Triandafilou KM, Kamper DG. Training finger individuation with a mechatronic-virtual reality system leads to improved fine motor control post-stroke. J Neuroeng Rehabil 11: 171, 2014. doi:10.1186/1743-0003-11-171. 25542201 PMC4292811

[272] Pandarinath C, Bensmaia SJ. The science and engineering behind sensitized brain-controlled bionic hands. Physiol Rev 102: 551–604, 2022. doi:10.1152/physrev.00034.2020. 34541898 PMC8742729

[273] Farina D, Vujaklija I, Branemark R, Bull AM, Dietl H, Graimann B, Hargrove LJ, Hoffmann KP, Huang HH, Ingvarsson T, Janusson HB, Kristjansson K, Kuiken T, Micera S, Stieglitz T, Sturma A, Tyler D, Weir RF, Aszmann OC. Toward higher-performance bionic limbs for wider clinical use. Nat Biomed Eng 7: 473–485, 2023. doi:10.1038/s41551-021-00732-x. 34059810

[274] Ortiz-Catalan M, Zbinden J, Millenaar J, D’Accolti D, Controzzi M, Clemente F, Cappello L, Earley EJ, Mastinu E, Kolankowska J, Munoz-Novoa M, Jonsson S, Cipriani C, Sassu P, Branemark R. A highly integrated bionic hand with neural control and feedback for use in daily life. Sci Robot 8: eadf7360, 2023. doi:10.1126/scirobotics.adf7360. 37820004

[275] Kutch JJ, Valero-Cuevas FJ. Muscle redundancy does not imply robustness to muscle dysfunction. J Biomech 44: 1264–1270, 2011. doi:10.1016/j.jbiomech.2011.02.014. 21420091 PMC3090003

[276] Valero-Cuevas FJ, Yi JW, Brown D, McNamara RV, III, Paul C, Lipson H. The tendon network of the fingers performs anatomical computation at a macroscopic scale. IEEE Trans Biomed Eng 54: 1161–1166, 2007. doi:10.1109/TBME.2006.889200. 17549909

[277] Long C, Conrad PW, Hall EA, Furler SL. Intrinsic-extrinsic muscle control of the hand in power grip and precision handling. An electromyographic study. J Bone Joint Surg Am 52: 853–867, 1970. doi:10.2106/00004623-197052050-00001. 5479476

[278] Valentin P. The interossei and lumbricals. In: *The Hand*, edited by Tubania R. Philadelphia, PA: W. B. Saunders Company, 1981, p. 244–253.

[279] Capsi-Morales P, Piazza C, Grioli G, Bicchi A, Catalano MG. The SoftHand Pro platform: a flexible prosthesis with a user-centered approach. J Neuroeng Rehabil 20: 20, 2023. doi:10.1186/s12984-023-01130-x. 36755249 PMC9906824

[280] Shafer A, Deshpande AD. Human-like endtip stiffness modulation inspires dexterous manipulation with robotic hands. IEEE Trans Neural Syst Rehabil Eng 30: 1138–1146, 2022. doi:10.1109/TNSRE.2022.3167400. 35420986

[281] Ajiboye AB, Willett FR, Young DR, Memberg WD, Murphy BA, Miller JP, Walter BL, Sweet JA, Hoyen HA, Keith MW, Peckham PH, Simeral JD, Donoghue JP, Hochberg LR, Kirsch RF. Restoration of reaching and grasping movements through brain-controlled muscle stimulation in a person with tetraplegia: a proof-of-concept demonstration. Lancet 389: 1821–1830, 2017. doi:10.1016/S0140-6736(17)30601-3. 28363483 PMC5516547

[282] Ethier C, Oby ER, Bauman MJ, Miller LE. Restoration of grasp following paralysis through brain-controlled stimulation of muscles. Nature 485: 368–371, 2012. doi:10.1038/nature10987. 22522928 PMC3358575

[283] Moritz CT, Perlmutter SI, Fetz EE. Direct control of paralysed muscles by cortical neurons. Nature 456: 639–642, 2008. doi:10.1038/nature07418. 18923392 PMC3159518

[284] Nason SR, Mender MJ, Vaskov AK, Willsey MS, Ganesh Kumar N, Kung TA, Patil PG, Chestek CA. Real-time linear prediction of simultaneous and independent movements of two finger groups using an intracortical brain-machine interface. Neuron 109: 3164–3177, 2021. doi:10.1016/j.neuron.2021.08.009. 34499856 PMC8549035

[285] Rouse AG. A four-dimensional virtual hand brain-machine interface using active dimension selection. J Neural Eng 13: 036021, 2016. doi:10.1088/1741-2560/13/3/036021. 27171896 PMC5776037

[286] Shah NP, Willsey MS, Hahn N, Kamdar F, Avansino DT, Hochberg LR, Shenoy KV, Henderson JM. A brain-computer typing interface using finger movements. Int IEEE EMBS Conf Neural Eng 2023: 10.1109/ner52421.2023.10123912, 2023. doi:10.1109/ner52421.2023.10123912. PMC10353344 37465143

[287] Wodlinger B, Downey JE, Tyler-Kabara EC, Schwartz AB, Boninger ML, Collinger JL. Ten-dimensional anthropomorphic arm control in a human brain-machine interface: difficulties, solutions, and limitations. J Neural Eng 12: 016011, 2015. doi:10.1088/1741-2560/12/1/016011. 25514320

[288] Ingram JN, Kording KP, Howard IS, Wolpert DM. The statistics of natural hand movements. Exp Brain Res 188: 223–236, 2008. doi:10.1007/s00221-008-1355-3. 18369608 PMC2636901

[289] Santello M, Bianchi M, Gabiccini M, Ricciardi E, Salvietti G, Prattichizzo D, Ernst M, Moscatelli A, Jorntell H, Kappers AM, Kyriakopoulos K, Albu-Schaffer A, Castellini C, Bicchi A. Hand synergies: Integration of robotics and neuroscience for understanding the control of biological and artificial hands. Phys Life Rev 17: 1–23, 2016. doi:10.1016/j.plrev.2016.02.001. 26923030 PMC5839666

[290] Davis TS, Wark HA, Hutchinson DT, Warren DJ, O’Neill K, Scheinblum T, Clark GA, Normann RA, Greger B. Restoring motor control and sensory feedback in people with upper extremity amputations using arrays of 96 microelectrodes implanted in the median and ulnar nerves. J Neural Eng 13: 036001, 2016. doi:10.1088/1741-2560/13/3/036001. 27001946

[291] Fan J, Vargas L, Kamper DG, Hu X. Robust neural decoding for dexterous control of robotic hand kinematics. Comput Biol Med 162: 107139, 2023. doi:10.1016/j.compbiomed.2023.107139. 37301095

[292] Luu DK, Nguyen AT, Jiang M, Drealan MW, Xu J, Wu T, Tam WK, Zhao W, Lim BZ, Overstreet CK, Zhao Q, Cheng J, Keefer EW, Yang Z. Artificial intelligence enables real-time and intuitive control of prostheses via nerve interface. IEEE Trans Biomed Eng 69: 3051–3063, 2022. doi:10.1109/TBME.2022.3160618. 35302937

[293] Guan C, Aflalo T, Kadlec K, Gamez de Leon J, Rosario ER, Bari A, Pouratian N, Andersen RA. Decoding and geometry of ten finger movements in human posterior parietal cortex and motor cortex. J Neural Eng 20: 036020, 2023. doi:10.1088/1741-2552/acd3b1. PMC10209510 37160127

[294] Hotson G, McMullen DP, Fifer MS, Johannes MS, Katyal KD, Para MP, Armiger R, Anderson WS, Thakor NV, Wester BA, Crone NE. Individual finger control of a modular prosthetic limb using high-density electrocorticography in a human subject. J Neural Eng 13: 026017, 2016. doi:10.1088/1741-2560/13/2/026017. 26863276 PMC4875758

[295] Jorge A, Royston DA, Tyler-Kabara EC, Boninger ML, Collinger JL. Classification of individual finger movements using intracortical recordings in human motor Cortex. Neurosurgery 87: 630–638, 2020. doi:10.1093/neuros/nyaa026. 32140722

[296] Overstreet CK, Cheng J, Keefer EW. Fascicle specific targeting for selective peripheral nerve stimulation. J Neural Eng 16: 066040, 2019. doi:10.1088/1741-2552/ab4370. 31509815

[297] Flesher SN, Collinger JL, Foldes ST, Weiss JM, Downey JE, Tyler-Kabara EC, Bensmaia SJ, Schwartz AB, Boninger ML, Gaunt RA. Intracortical microstimulation of human somatosensory cortex. Sci Transl Med 8: 361ra141, 2016. doi:10.1126/scitranslmed.aaf8083. 27738096

[298] Shelchkova ND, Downey JE, Greenspon CM, Okorokova EV, Sobinov AR, Verbaarschot C, He Q, Sponheim C, Tortolani AF, Moore DD, Kaufman MT, Lee RC, Satzer D, Gonzalez-Martinez J, Warnke PC, Miller LE, Boninger ML, Gaunt RA, Collinger JL, Hatsopoulos NG, Bensmaia SJ. Microstimulation of human somatosensory cortex evokes task-dependent, spatially patterned responses in motor cortex. Nat Commun 14: 7270, 2023. doi:10.1038/s41467-023-43140-2. 37949923 PMC10638421

[299] Greenspon CM, Valle G, Hobbs TG, Verbaarschot C, Callier T, Okorokova EV, Shelchkova ND, Sobinov AR, Jordan PM, Gonzalez-Martinez J, Warnke PC, Miller LE, Boninger ML, Collinger JL, Gaunt RA, Downey JE, Hatsopoulos NG, Bensmaia SJ. Biomimetic multi-channel microstimulation of somatosensory cortex conveys high resolution force feedback for bionic hands (Preprint). bioRxiv 2023.02.18.528972, 2023. doi:10.1101/2023.02.18.528972. 36824713 PMC9949113

[300] Mazurek KA, Schieber MH. Injecting information into the mammalian cortex: progress, challenges, and promise. Neuroscientist 27: 129–142, 2021. doi:10.1177/1073858420936253. 32648527 PMC7995349

[301] Quallo MM, Kraskov A, Lemon RN. The activity of primary motor cortex corticospinal neurons during tool use by macaque monkeys. J Neurosci 32: 17351–17364, 2012. doi:10.1523/JNEUROSCI.1009-12.2012. 23197726 PMC3678117

[302] Cerkevich CM, Rathelot JA, Strick PL. Cortical basis for skilled vocalization. Proc Natl Acad Sci U S A 119: e2122345119, 2022. doi:10.1073/pnas.2122345119. 35507879 PMC9171651

[303] Strick PL, Dum RP, Rathelot JA. The cortical motor areas and the emergence of motor skills: a neuroanatomical perspective. Annu Rev Neurosci 44: 425–447, 2021. doi:10.1146/annurev-neuro-070918-050216. 33863253

[304] Laurence-Chasen JD, Ross CF, Arce-McShane FI, Hatsopoulos NG. Robust cortical encoding of 3D tongue shape during feeding in macaques. Nat Commun 14: 2991, 2023. doi:10.1038/s41467-023-38586-3. 37225708 PMC10209084

[305] Simonyan K. The laryngeal motor cortex: its organization and connectivity. Curr Opin Neurobiol 28: 15–21, 2014. doi:10.1016/j.conb.2014.05.006. 24929930 PMC4177508

[306] Simonyan K, Cho H, Hamzehei Sichani A, Rubien-Thomas E, Hallett M. The direct basal ganglia pathway is hyperfunctional in focal dystonia. Brain 140: 3179–3190, 2017. doi:10.1093/brain/awx263. 29087445 PMC5841143

[307] Brandauer B, Hermsdorfer J, Geissendorfer T, Schoch B, Gizewski ER, Timmann D. Impaired and preserved aspects of independent finger control in patients with cerebellar damage. J Neurophysiol 107: 1080–1093, 2012. doi:10.1152/jn.00142.2011. 22114161

